# Cheliceral chelal design in free-living astigmatid mites

**DOI:** 10.1007/s10493-021-00625-3

**Published:** 2021-05-14

**Authors:** Clive E. Bowman

**Affiliations:** Mathematical Institute, University of Oxford, Oxford, OX2 6GG UK

**Keywords:** Actinotrichida, Ecomorphology, Feeding, Geometric morphometrics, Individualised divergences, Mechanical advantage, Ordination, Shape, Size

## Abstract

Cheliceral chelal design in free-living astigmatid mites (Arthropoda: Acari) is reviewed within a mechanical model. Trophic access (body size and cheliceral reach) and food morsel handling (chelal gape and estimated static adductive crushing force) are morphologically investigated. Forty-seven commonly occurring astigmatid mite species from 20 genera (covering the Acaridae, Aeroglyphidae, Carpoglyphidae, Chortoglyphidae, Glycyphagidae, Lardoglyphidae, Pyroglyphidae, Suidasiidae, and Winterschmidtiidae) are categorised into functional groups using heuristics. Conclusions are confirmed with statistical tests and multivariate morphometrics. Despite these saprophagous acarines in general being simple ‘shrunken/swollen’ versions of each other, clear statistical correlations in the specifics of their mechanical design (cheliceral and chelal scale and general shape) with the type of habitat and food consumed (their ‘biome’) are found. Using multivariate analyses, macro- and microsaprophagous subtypes are delineated. Relative ratios of sizes on their own are not highly informative of adaptive syndromes. Sympatric resource competition is examined. Evidence for a maximum doubling of approximate body volume within nominal taxa is detected but larger mites are not more ‘generalist’ feeding types. Two contrasting types of basic ‘Bauplan’ are found differing in general scale: (i) a large, chunk-crunching, ‘demolition’-feeding omnivore design (comprising 10 macrosaprophagous astigmatid species), and (ii) a small selective picking, squashing/slicing or fragmentary/‘plankton’ feeding design (which may indicate obligate fungivory/microbivory) comprising 20 microsaprophagous acarid-shaped species. Seventeen other species appear to be specialists. Eleven of these are either: small (interstitial/burrowing) omnivores—or a derived form designed for processing large hard food morsels (debris durophagy, typified by the pyroglyphid *Dermatophagoides farinae*), or a specialist sub-type of particular surface gleaning/scraping fragmentary feeding. Six possible other minor specialist gleaning/scraping fragmentary feeders types each comprising one to two species are described. Details of these astigmatid trophic-processing functional groups need field validation and more corroborative comparative enzymology. Chelal velocity ratio in itself is not highly predictive of habitat but with cheliceral aspect ratio (or chelal adductive force) *is* indicative of life-style. Herbivores and pest species are typified by a predicted large chelal adductive force. Pest species may be ‘shredders’ derived from protein-seeking necrophages. *Carpoglyphus lactis* typifies a mite with tweezer-like chelae of very feeble adductive force. It is suggested that possible zoophagy (hypocarnivory) is associated with low chelal adductive force together with a small or large gape depending upon the size of the nematode being consumed. *Kuzinia laevis* typifies an oophagous durophage. Functional form is correlated with taxonomic position within the Astigmata—pyroglyphids and glycyphagids being distinct from acarids. A synthesis with mesostigmatid and oribatid feeding types is offered together with clarification of terminologies. The chelal lyrifissure in the daintiest chelicerae of these astigmatids is located similar to where the action of the chelal moveable digit folds the cheliceral shaft in uropodoids, suggesting mechanical similarities of function. Acarid astigmatids are trophically structured like microphytophagous/fragmentary feeding oribatids. Some larger astigmatids (*Aleuroglyphus ovatus*, *Kuzinia laevis*, *Tyroborus lini*) approximate, and *Neosuidasia* sp. matches, the design of macrophytophagous oribatids. Most astigmatid species reviewed appear to be positioned with other oribatid secondary decomposers. Only *Dermatophagoides microceras* might be a primary decomposer approximating a lichenivorous oribatid (*Austrachipteria* sp.) in trophic form. Astigmatid differences are consilient with the morphological trend from micro- to macrophytophagy in oribatids. The key competency in these actinotrichid mites is a type of ‘gnathosomisation’ through increased chelal and cheliceral height (i.e., a shape change that adjusts the chelal input effort arm and input adductive force) unrestricted by the dorsal constraint of a mesostigmatid-like gnathotectum. A predictive nomogram for ecologists to use on field samples is included. Future work is proposed in detail.

## Introduction

Mites appear to have always been small over geological time (Sidorchuk 2018). Body size can fundamentally shape an organism’s ecological niche, a population’s rate of evolution, and an ecological community’s structure and function (Kaspari 2005). Size matters in mites (Seeman and Nahrung 2018). This is not only true of predators (Bowman 2021) but also herbivorous animals that handle and consume particulate matter (such as most astigmatids; Walter and Proctor 1998). Vertebrate herbivores have various distinct shapes according to their grazing roles (Veitschegger et al. 2018)—acarines ought to be no different. Of course, what mites of different sizes and shapes chew on is not necessarily the same as what mites really ingest, digest and assimilate. However, analysis of the dietary relationships within an assemblage of organisms can provide a variety of information on ecological processes (Rotenberry 1980). Carbohydrates are needed for energy to sustain life, proteins are needed for growth and reproduction. If food is a limiting resource then its supply to metabolism should play a major role in determining community structure, as that ‘niche dimension’ will become prone to direct competitive diet selection.

Niche differentiation can be detected not only from morphology (e.g in birds; Grant 1986, cichlid fish; Bouton et al. 2002, or lizards; Bickel and Losos 2002) but indirectly without actually observing feeding (Schneider et al. 2004). Animal tissues (including soil nematodes) are full of proteinaceous and carbohydrate material suitable as high energy food. Fungal tissues also have a high nutritive potential. Although they are rather similar to plant foliage in overall nutrient makeup, their tissues are very different in terms of constituent chemical structures. All of these trophic sources need trituration and enzymic breakdown. Many acarologists have attempted to link mite form to its function in comparative studies on resource utilisation. They have used indirect information from fatty acids (neutral lipid fatty acids, NLFA), amino acids, enzymes, or physical and molecular gut content analyses of field samples. Detractors have claimed that mite morphology (and laboratory feeding tests) have little to offer in dissecting out trophic roles in mites, yet such are equally also indirect surrogates of what is actually happening as to how mite gnathosomas work in the wild. Recently, Perdomo et al. (2012) using stable radio-isotope signatures (^15^N, ^13^C) in field samples have unequivocally validated the predictive power of mouthpart morphology in oribatids in highlighting their trophic role in soil. This “function-informed” morphometric review (Feilich and López-Fernández 2019) builds upon this together with various investigations of astigmatids carried out by historical acarologists.

### Why astigmatids?

**Fig. 1:**
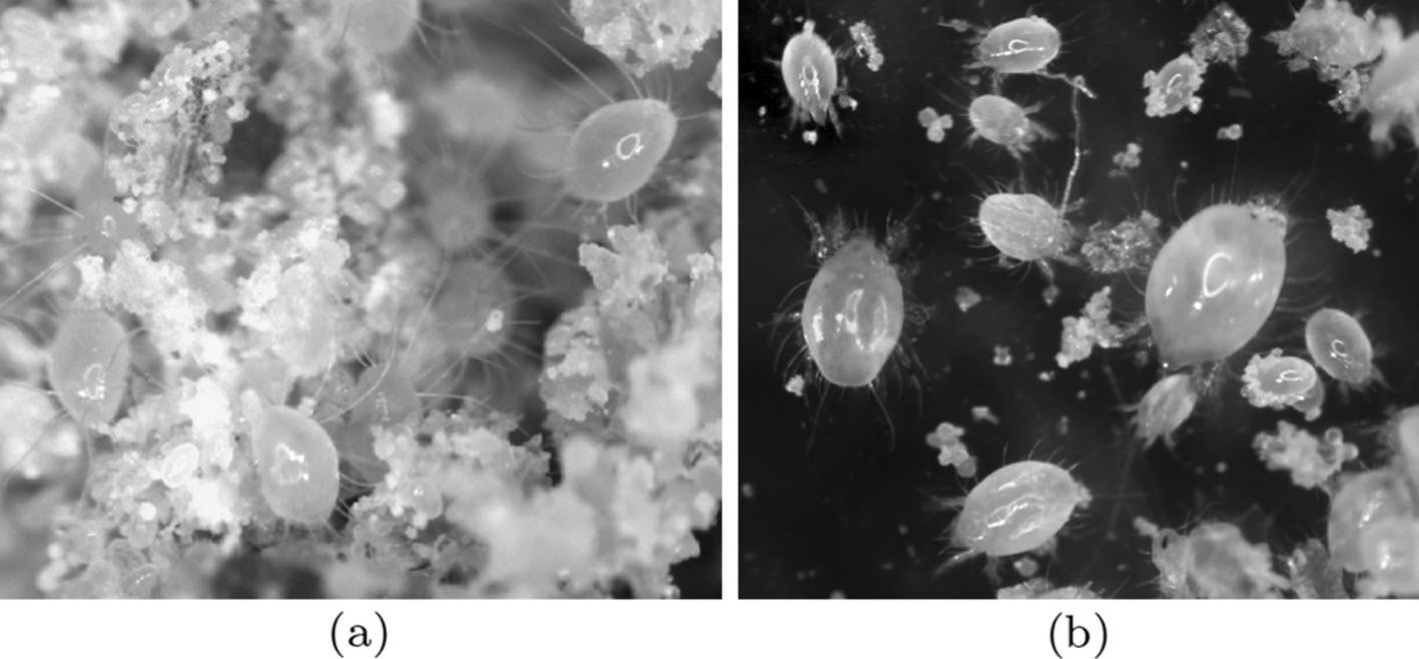
Typical free-living saprophagous astigmatid mites. **a** *Blomia tropicalis*. **b** *Chaetodactylus krombeini*. From colour photographs ex Pavel Klimov with permission

Free-living saprophagous astigmatid mites (or acarines)—small ($<1\,\text {mm}$) arachnids recognised by their lack of stigmata or external breathing pores—include many pest species consuming stored human foodstuffs (Evans 1992; Baker 1999). They are phylogenetically close to oribatids (Krantz and Walter 2008). They are cosmopolitan, eight-legged, often pearl-white, ovate, sometimes hairy arthropods lacking strong segmentation (Fig. 1). Many are *r*-strategists showing explosive population growth in good environmental conditions. Some taxa appear to specialise on one resource within a habitat (e.g., water-filled tree-holes for *Naidacarus* spp.; Fashing and Chua 2002), while others are more catholic (OConnor 1982a, b). Most species are deemed fungivorous and commonly occur as detritivorous saprophages in decaying organic matter within soil, but some species are also facultatively phytophagous (Evans et al. 1961; Krantz and Lindquist 1979 gives a good summary). Others have particular restricted diets or are associates of various insects, or inhabitants of vertebrate nests (see Hughes 1976 for detailed food preferences and habitats related to storage mites). Luxton (1981) gives a good review of their occurrence in soils where *Rhizoglyphus* spp. and surprisingly *Glycyphagus ornatus* are claimed to be common. There is a general tendency for them to select moist conditions and high protein food when it is available (Hughes 1959). Facultative predation is an opportunity for any fungivore (Walter and Proctor 2013). Small defenceless protozoa, rotifers, nematodes and other micro-invertebrates could be accidentally or purposefully consumed during astigmatid feeding. Considered monophyletic, free-living astigmatids have a general uniformity of body structure, however, prediction of their habits from their morphology would be of use to field ecologists.

**Fig. 2:**
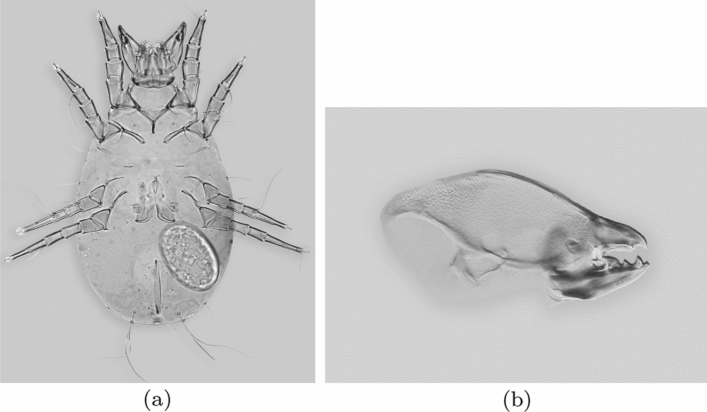
Typical astigmatid mite form, from colour photographs ex Pavel Klimov with permission. **a** *Acarus siro* female. Slide mounted cleared specimen from the ventral side showing squashed-out paired chelicerae protruding anteriorly in its gnathosoma (mouthparts to upper central). **b** Enlarged lateral view of a chelicera of *Chaetodactylus krombeini*. Note dentate chela to right end of cheliceral shaft. Tendons and musculature inside the cheliceral base actuate the (lower) moveable digit against the (upper) fixed digit. The basal part of the moveable digit is extended vertically like a coranoid process or ascending ramus in a vertebrate mandible (Morales-García et al. 2021). The gleaming actinochitinous nature of the digits points to their evolutionary origin from setae/ambulacra (Grandjean 1947)

**Fig. 3:**
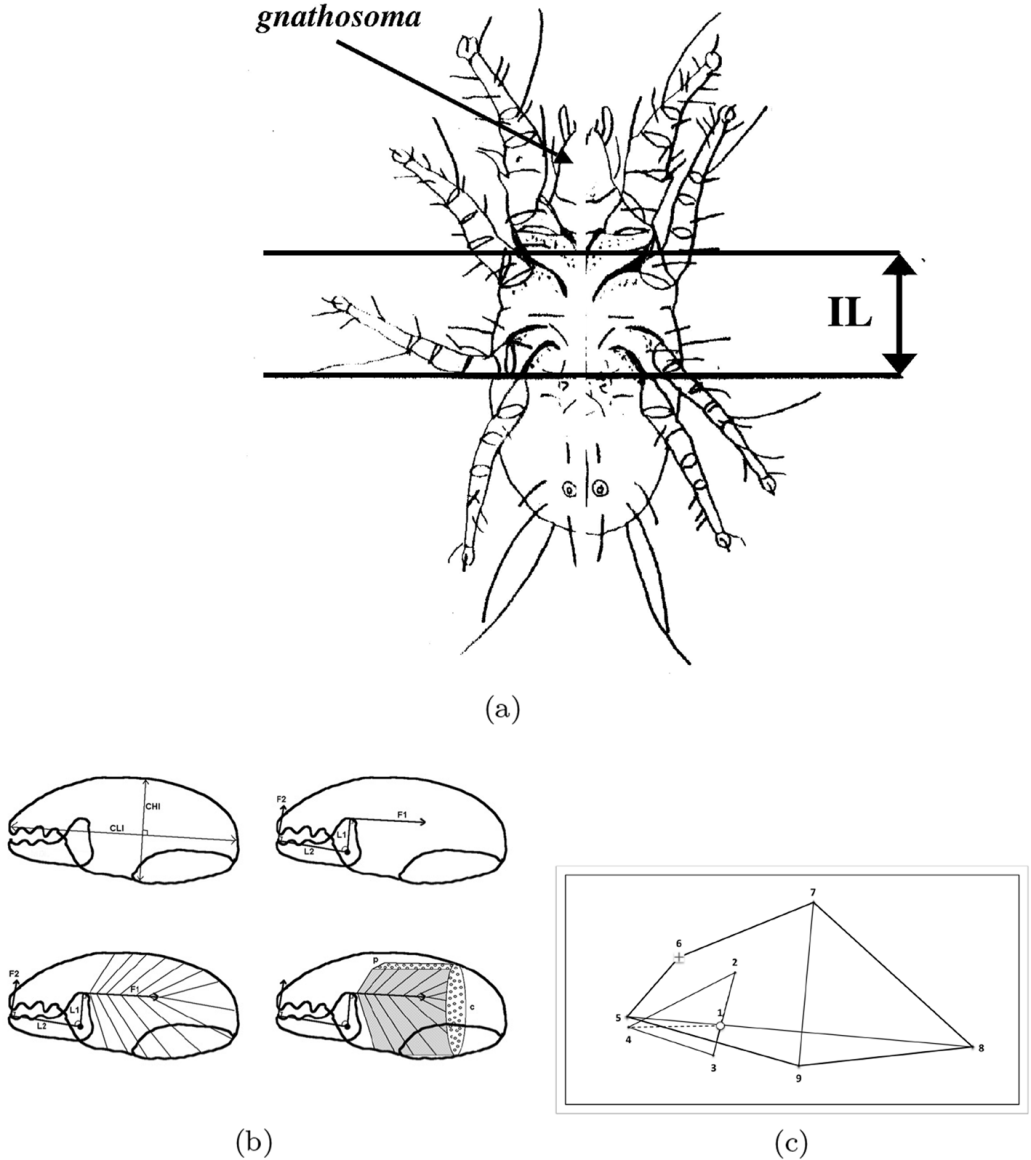
Mite size definition and the mechanical model of static forces based upon rigid levers used in review. **a** Measurement of index of idiosomal length (*IL*)—amended after Griffiths et al. (1990). This intercoxal distance is indicative of overall idiosomal length ($\approx$ size of the mite) and is not prone to distortion on slide mounting. **b** Stylised chelicera after Knülle (1959). *Upper row* showing measurement of: *Left*: Cheliceral parameters (length *CLI* $\equiv$ reach, height *CHI*). *Right* Chelal parameters (fixed digit upper input lever arm L1, moveable lever output arm L2 $\approx$ gape, chelal crunch force *F*2). *F*1 is the estimated force on the adductive tendon due to cheliceral musculature. The rigid moveable digit rotates on chelal closing around a condyle (small circle) inside the chelicera actuated by musculature attached to the tendon. *Lower row* Schema showing two assumptions of closing muscle topology. *Left* Cheliceral base full of fibres. *Right* $\text {p} =$ pennate force $F1P \propto CHI*CLI$, $\text {c} =$ circular force $F1C \propto CHI^2$ (Perdomo et al. 2012), used in calculating the adductor static force *F*1 as dependent upon a nominal cheliceral muscle cross-sectional area. Final crunch forces F2P and F2C are obtained by pre-multiplying with the velocity ratio $\frac{L1}{L2}$. Then $F2AV=\frac{F2P+F2C}{2}$. **c** Wire-frame of an individual *Neosuidasia* sp (LA1) showing nine landmarks used in geometric morphometrics. Condyle at open circle. Moment arms dotted. Moveable digit shape, *CHI* and *CLI* added by joining landmarks. Note dorsal lyrissure in cheliceral shaft above moveable digit indicated by small cross

Astigmatid mouthparts are similar to those of oribatids being comprised of paired chelicerae with usually grasping chelae (Evans et al. 1961) in a gnathosoma (Fig. 2a). Each chela has a fixed (immovable) digit with teeth opposing a moveable dentate digit (Fig. 2b) which rotates in a condyle. As in oribatids (Schuster 1956), changes in turgor pressure within the cheliceral base (together with a small abductor muscle) opens the chelal digits, while large adductor musculature within the broadly tubular cheliceral shaft closes them to crush food (Fig. 3b). The chelae alternately break up foodstuffs (aka ‘comminute’; Evans 1992) and independently convey solid material to the mite’s mouth inside the gnathosoma (Fig. 3a) for ingestion. Gnathosomal muscles connected to the cheliceral base ventrally a little behind the condyle pull the whole food-laden chelicera independently both down and back into the body (Grandjean 1947) shovelling the morsels into the gnathosoma much like a primitive arachnid (Van der Hammen 1971). Fluid pressure within it, or spring-action/elasticity of the cuticle (as in scorpion chelae; Dubale and Vyas 1968), protracts the chelicera. Subcapitular rutella (Alberti 2008) and a labrum mechanically process the food, compacting it or tearing it into small pieces (Akimov 1979) before it enters the oral groove or mouth (Evans 1992). The protracting chelicera takes an upper (dorsal) position relative to that of the retracting chelicera (Evans 1992). Compared to the Anactinotrichida, cheliceral retraction into the idiosoma is modest. There is typically no gnathotectum (i.e., no dorsal restriction to the height of chelicerae) unlike in mesostigmatids.

**Fig. 4:**
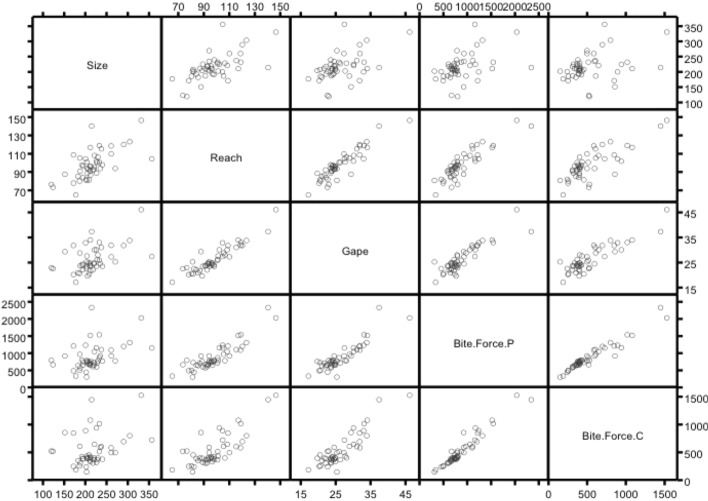
Lattice plot of average measurements across astigmatid species in this review showing general correlation for size (*IL*), reach (*CLI*), gape (*L*2*M*) and separate pennate (P) and circular (C) chelal crunch forces (F2 ‘Bite’ Force.’). Note strong correlation of two alternative ways of calculating crunch forces

Perdomo et al. (2012) points to cheliceral morphology being an inexpensive quick filter for the estimation of dietary preferences in mites. Their Fig. 4 is consilient with the historical conclusions of Schuster (1956) and Kaneko (1988). Decomposing plant material is assumed to take a long time due to its intractability to chemical breakdown compared to say catabolising an animal carcass. Oribatids are divided up into ‘carnivores’, primary decomposers and secondary decomposers by Perdomo et al. (2012). This review examines the comparative morphology of free-living astigmatids to see if they agree. To what extent these astigmatids can be further classified ecomorphologically into guilds (Root 1967) as has been done for soil-dwelling oribatids (Luxton 1972) to be: macrophytophages, microphytophages, panphytophages, zoophages, coprophages, or necrophages is not clear. Similarly what trophic dimensions to deploy in any new study not looking at oribatids is not clear.

Would Böttger (1970)’s approach of: predator, parasite, carrion-feeder (scavenger), plant feeder, omnivore, detritus feeder be better? Or even Evans (1992)’s: zoophagy, phytophagy (herbivory/mycetophagy), omnivory, and saprophagy (detritivory)? Perhaps Fashing (1998)’s categorisation of: ‘shredders’ who ingest leaf material and associated microbes by biting off chunks of leaves, ‘scrapers’ (grazers?) who crop fungal hyphae and/or other microbes and detritus from the substrate surface, and ‘collectors’ who filter microbes and fine particulate matter from aquatic films should be used? In practice all of these overlap, with various trophic sources used as alternatives or supplements by the mites concerned. What is stable, for at least oribatids, even in the face of trophic plasticity (i.e., adjustment of their exact diet in different habitats and ecosystems Maraun et al. 2020) is their *role* (usually detritivore in this instance). So, does one fit species to a pre-existing trophic classification (i.e., a set of roles), or does one let the design of mites with known lifestyles define the appropriate trophic dimensions for any functional groups?^1^ By using heuristics this review first takes the latter ’hypothesis-free’ approach. Then this is underpinned with formal statistical tests around specific hypotheses.

## Aim

The aim of this review is first to display morphological dimensions of potential interest in understanding feeding for a large number of astigmatids in an optimal ordination. Then to understand where species of different feeding types and taxonomic position fit in this analysis using arguments around the mechanics of food processing. Then to test hypotheses statistically, and finally to provide a comparative synthesis of astigmatid trophic designs and roles with those of mesostigmatids and oribatids.

## Rationale for experimental approach

Taking just the free-living Astigmata alone there are $> 400$ genera, and $> 1300$ species (Krantz and Walter 2008). As Futuyma (1979) says: “...ecological specialisation is unquestionably the rule in species-rich groups of organisms...”. This should be true of astigmatids as much as for cichlid fishes (Fryer and Iles 1972). A tacit assumption in much of biology is that form, that is size and shape, is related to function. Whether this is in an optimal way or not has been hotly debated over the years (see for example Rosen 1967). However, the adaptive significance of an organism’s size and shape to its ecology has not been in question for decades (e.g., Barton et al. 2011). For mites, Schuster (1956) in his seminal work classifying vegetarian oribatids into feeding classes established a link of diet with the relative proportions of chelicera to chelae (i.e., fd/md where fd = cheliceral length $\equiv$ reach, and md = moveable digit length $\equiv$ gape). Low [cheliceral reach/moveable digit gape] values for (robust powerful) oribatid chelicerae indicated macrophytophagy, high [reach/gape] values (i.e., elongate delicate cheliceral chelae) indicated microphytophagy. Matters may be more complicated as Smrž (2010) has shown from enzyme assays that even amongst mycophagous oribatids different styles of oral food processing must occur. Other authors have gone down a similar morphological route (e.g., Buryn and Brandl 1992 and Adar et al. 2012 for some mesostigmatids; Akimov and Gaichenko 1976 for a few astigmatids). This latter group of mites comprises distinct well-known families such as Acaridae, Glycyphagidae, etc., which are traditionally described as saprophages (i.e., feeding on or obtaining nourishment from decaying organic matter) rather than phytophages (i.e., feeding on plants). Some are well known pests of stored human foodstuffs (e.g., cheese, fish, grain, meat, etc.,).

The underlying adaptionist concept (Manton 1958) of this review is that astigmatid chelal morphological form together with that of their chelicerae *should* be correlated with the preferred food type of each species and thus the habitats that they live in. That is, their biological phenotype (*B*) is a function of the mite’s trophic design (*D*), which in turn is a function of its morphology (*M*). The niche each species occupies within its community is those ranges and combinations of environmental conditions that permit persistent existence (Root 1967). Being able to predict an unknown mite’s likely biology from its morphology is useful to ecologists on collecting a new species ($u_{i}$) in the field (i.e., being able to estimate ${\hat{B}}[D[M(u_{i})]]$ where $\hat{\,}$ indicates ‘estimated’). This is what Perdomo et al. (2012) has vindicated. Then one can investigate how mite communities assemble allopatrically or sympatrically (like that done in other animals; Johnson 1966; Schoener 1970) and build upon the pioneering work of Akimov and Oksentyuk (2018).

Principal component analyses often indicate suites of morphological components (*M*) related to ecology (e.g., Wiens and Rotenberry 1980). However, these empirical analyses do not necessarily take on-board any mechanical consequences of any feeding system. A simple engineering model widely used to analyse evolutionary adaptation for handling different foodstuffs in animals is to assume a model of static forces based upon rigid levers (Alexander 1983). Previously applied to larger animals (Smith and Savage 1959), like in Perdomo et al. (2012) it is used herein (Fig. 3b). In particular, mechanics is used to try to explain why some astigmatid species are pests and others are not. The experimental concept of this review is that any trophic correlation (*B*) is informed by estimates of chelal crushing force (${\hat{D}}$) as well as by morphology (*M*). Furthermore, whether taxonomic position is important in astigmatid trophic adaptation will also be examined.

A mite’s moveable digit within its chela rotates in a condyle (Fig. 3b) within the cheliceral base as the chela opens and closes. This is much like the general action of a vertebrate jaw. Ecomorphological studies of animal jaws have been remarkably insightful (for a recent entry-point to that literature see Morales-García et al. 2021). In the static model of jaws (or crustacean/arachnid chelae; Bowman 2021), the velocity ratio (*VR*) is defined by the ratio of the two lever or moment arms: the in-lever (or effort arm; Perdomo et al. 2012) *L*1, and the out-lever effort arm *L*2 (irrespective of the angle between them); Warner and Jones (1976). This velocity ratio determines the ideal mechanical advantage of the system of orthogonal static forces at equilibrium assuming a frictionless system (Brown et al. 1979).

Muscular force in animals is usually a function of the cross-sectional area of myocyte fibres. The cheliceral base in mites is packed full of pennate muscles attached at two points to the chela by short tendons. One (the lower point) is for the opening abduction (moveable digit depression) muscles, the other (the upper point) is for the adductive chelal closing muscles. So any chelal closing force should scale with some function of cheliceral base cross-section (Fig. 3b). As the chela rotates in the condyle, the resultant crunching force (*F*2) on foodstuffs between the cheliceral digits is tangential—i.e., any difference from $\frac{\pi }{2}$ radians in the tendon angle effectively rectifies the initial adductive force on the tendon (*F*1). Mite holding forces are hard to measure (Heethoff and Koerner 2007) but can be much higher than expected for organisms of their size, only being exceeded by those of crustacea. Perdomo et al. (2012) use a very simple formula for such a force estimate (i.e., $PHI^2$). Herein, two other estimates of nominal static closure force will be also used in this study depending upon different micro-anatomical assumptions to derive a consensus estimate comparable across different animals (Bowman 2021).

Whilst digestive specialisation is well-known in astigmatids (Bowman 1981; Childs and Bowman 1981; Bowman and Childs 1982; Bowman 1984; Bowman and Lessiter 1985; Erban and Hubert 2008, 2009), and many astigmatid mouthparts have been well described (Akimov 1973, 1975, 1977, 1979), the relationship between trophic function and the mechanical form of their chelicerae has only been examined once before (Akimov and Gaichenko 1976) and never rigorously over many different species. In order to examine the between-species morphological variation (*M*), this review uses a radial ordination method based upon the information for each species that each cheliceral character contains for the distinction which that mite design (*D*) has to a notional central common ‘Bauplan’ (body plan). This is an extension of the methods from Bowman (2015a). Ecological and taxonomic correlates (*B*) are laid over an SVD (singular value decomposition) ordination of the correlation space of comparative morphological (*M*) and design (*D*) information in order to draw conclusions. This approach is favoured because the niche for at least some of the mites is expected to be highly multidimensional (Root 1967). It is a methodology that allows the mixing of data types without needing algorithmic adjustment.

## Expected results

Putting aside the variety of ways that the sub-capitular structure as a whole is designed for the ingestion of different types of food (Akimov 1985), at a functional level the food an animal like a mite can access is determined by:

- the size of the animal (i.e., its scale), and
- its mouthparts’ reach.

Similarly, the food an animal’s mouthparts are able to handle when foraging depends upon:

- the gape of its food gripping apparatus, and
- the force which it can apply to break up foodstuff.

Should an animal change size (*s*) but retain the same general shape, structures of the same dimensionality should scale linearly. Lack of proportionality between sub-structures on changes in size indicates a change in shape. For instance a mite may show a disproportionately large or small gnathosoma in some way given its size compared to a basal standard form. In practice the constituent parts of animals often show allometry. For a critical introduction to allometry (and geometric similarity) see Gould (1971). Healthy adult individuals of different species are generally not geometrically similar if:

(i) they are adapted to different ways of life,
(ii) they are descended from different ancestors,
(iii) or, such similarity is not consistent with the optimal design of animals of different sizes (Alexander et al. 1981).

At modest scale differences, allometry often does not apply and animals can be just different sized linear versions (‘shrinkings/swellings’) of each other. Nature exhibits concerted evolution over all aspects of an organism’s phenotypic form and its function. Ecology ‘sees’ the real *instantiated* size and shape of animals, not the underlying mechanism of their fundamental growth form. Evolution on the developmental mechanism selects that latter instantiation.

Cheliceral structure and feeding ecology do not need to be strongly correlated of course. What a mite ingests and what it chews on is not necessarily the same as what it really digests and assimilates. Accordingly, the first two topics, (i) and (ii) above, are explored via a simultaneous analysis of the mites’ actual size, reach, gape (*M*) and crunch force (${\hat{D}}$) without any allometric adjustment initially (i.e., the power exponent is set to one). SVD ordinations allow one to explore what structures are linearly correlated in magnitude and which are not. This review deploys a recent entropy-based multivariate method (Delrieu and Bowman 2005, 2006a, b, 2007; Bowman et al. 2006; Charalambous et al. 2008; Bowman and Delrieu 2009; Bowman 2009, 2015b) derived from the taxonomic work of Jardine and Sibson (1971). Based upon an explicit reference group (like in cladistic-style systematics; Watrous and Wheeler 1981) and related to canonical correlation, this mathematics has the advantage of explicitly folding the research question posed (i.e., the contrast of interest) into the *directions* of the singular value decomposition (SVD) ordination of the data correlations (Bowman 2015a). As well as being probabilistically rigorous, this allows adjunct variables which facilitate the direct interpretation of between-species phenotypic displays and heat-maps (Bowman 2013). No attempt in this review is made to investigate intra-populational variation (Johnston and Selander 1971) or to use such to either understand (Herrel et al. 2001) or predict feeding behaviour (Smartt and Lemen 1981).

Taking an optimally adaptationist stance then, all other matters being equal, large mites are assumed to be physically restricted to inhabiting the surfaces of food, while small mites might burrow and exploit interstitial cavities too. Mites with a long reach could dig in material or utilise food in crevices, while acarines with a short reach would be forced to rely upon gleaning and browsing easily accessible food. Irrespective of whether they graze and digest fragments of fungi including their chitinous wall or simply cut and ingest hyphae, only digesting their cellular content (Smrž 2010), astigmatids with a large chelal gape should be able to grasp objects of a larger size (i.e., be macrosaprophagous species) than those objects grasped by animals with only a small gape (i.e., the latter being only microsaprophagous species). In particular, those with an especially small gape may be restricted to essentially gleaning ‘planktonic’ morsels or microbes. Those with a proportionately large gape might stab food with a piercing closed chela like a spear and lap up exuding fluids or suck out its contents (as Smrž 2010 recounts). For sure, bigger species with bigger mouthparts should be expected to eat bigger and more variable size food items (Wiens and Rotenberry 1980 and other references therein). Any mite capable of a large static crunch force between its chelal digits clearly has the possibility of demolishing and cracking harder foodstuff than those acarines exhibiting lower forces who may be restricted to gently squashing or slicing soft food. This argument is exactly that behind Perdomo et al. (2012)’s Fig. 4 delimiting ‘carnivores’, primary decomposers and secondary decomposers in oribatids. Indeed, some mites may ‘pack a punch’ much bigger than one would expect for that size.

Microbivore–detritivore animals are especially difficult to assign to a simple trophic category (Walter and Proctor 2013), so a specific hypothesis will be examined:

Could a pair of simple heuristic ‘four-box models’ of trophic design (over *M* and *D*) explain astigmatid life-histories (*B*)?

To whit:

1. One ‘four-box model’, based upon *food access* (i.e., size and reach), contrasting a surface or interstitial habit versus crevice/excavation or browse/glean feeding.
2. The other ‘four-box model’, based upon *morsel handling* (i.e., gape and crunch force), contrasting a macro- or microsaprophagous choice versus soft or hard food consumption.

Then, within these two binary splits, topics such as herbivory/phytophagy, fungivory, copro-/necrophagy, zoophagy, and other foraging specialisms will be critically examined. Functional groups (not ‘guilds’; Walter and Proctor 2013) of species without regard to taxonomic position (Root 1967), will be posed based upon each species sharing the same ‘adaptive syndromes’. Adaptive syndromes are co-ordinated sets of characteristics including the specific manner of likely resource utilisation and array of related adaptations (Eckhardt 1979). Derived measures over *M* and *D* will be calculated and further ‘four-box models’ formed as required. Statistical tests of specific hypotheses will be made. This will lead finally to a nomogram (i.e., a first pass filter) suitable for field ecological predictions and an exemplified synthesis of astigmatid feeding types in the context of mesostigmatids and oribatids. The features that mark out astigmatid species as pests will be delineated.

Perfection in design is not expected, nor will strong conclusions be made between one particular species versus another nor any claims made for sub-structuring into small communities, as even in Perdomo et al. (2012) only broad groupings are delineated. As Futuyma (1979) says: “Biological thought is so permeated by the recognition that many, perhaps most, features have functions that we often forget that organisms are not perfect. In many ways they are suboptimally constructed compared with the ideal forms that an engineer might design”.

## Materials and methods

### Materials

Twenty female adults each of forty-seven species of free-living saprophagous astigmatid mites were examined (originated from the wild or from live cultures kept at the now defunct Pest Infestation Control Laboratory, Slough, UK: see Table 1). These had been collected and nurtured for many years by the late Dr Donald Griffiths and his team investigating principally their taxonomy. Their identification was originally carried out almost 50 years ago and astigmatid taxonomy has moved on several times since, courtesy of efforts by a variety of acarologists and in particular those of Barry OConnor at University of Michigan. This review attempts to use modern names in its write-up but still refers to the cultures by their original ‘short-hand’ code so as to allow traceability back to source and comparison to existing publications featuring them. The *Tyrophagus* spp. breeding groups follow Griffiths (1979).

**Table 1:** Details of astigmatid species reviewed

Species identification on collection in [...]	Culture number	Origin	Where found	Date
*Acarus chaetoxysilos *Griffiths	AC204	Hartington, Derbyshire, GB	Cheese store	1973
*Acarus farris* (Oudemans)	A17	GB	Haystack	1975
*Acarus gracilis* Hughes	A4	*Recorded as*: ’Unknown’	*Recorded as*: ’Unknown’	*Recorded as*: ’Unknown’
*Acarus immobilis* Griffiths	A1	Slough, Berkshire, GB	Robin’s nest	1966
*Acarus siro* L. [’H’]	A10b	GB	Pigmeal	1940’s
*Acarus siro* L. [SW sp.]	A15	GB	Ex Survey W.D./2	1973
*Aleuroglyphus ovatus* (Troupeau)	AL2	Bristol, Avon, GB	Mill	July 1956
*Sancassania berlesei* (Michael) [was *Caloglyphus berlesei* (Michael)]	C3	Karachi, Pakistan	Fishmeal	*Recorded as*: ’Unknown’
*Cosmoglyphus oudemansi* Zachvatkin [was *Caloglyphus oudemansi* (Zachvatkin)]	C10	Paraiba, NE Brazil	Dried Cactus	19/01/80
*Cosmoglyphus hughesae* Samsinak [was *Caloglyphus redikozevi* (Zachvatkin)]	C5	*Recorded as*: ’Unknown’	*Recorded as*: ’Unknown’	1968
*Kuzinia laevis* (Dujardin)	KL or $	*Recorded as*: ’Not known’	*Recorded as*: ’Not known’	*Recorded as*: ’Not known’
*Lardoglyphus konoi* (Sasa and Asanuma)	L1	Greshik, E Java	Goat skins	10/10/72
*Lardoglyphus zacheri* Oudemans	L3	Manchester, Cheshire, GB	Mexican Daphnia Meal	5/10/79
*Neosuidasia* sp. [was *Lackerbaueria* sp.]	LA1	Mauritius	Poultry House Sweepings	4/11/80
*Madaglyphus legendrei* (Fain) [was *Comerinia chaetolamina* Ferguson]	T34	Mexico	Dried Daphnia	19/1/71
*Rhizoglyphus echinopus* (Fumouze & Robin) [was *Rhizoglyphus callae* Oudemans]	R2	Holland	Tulip Bulbs	13/11/73
*Rhizoglyphus echinopus* (Fumouze & Robin) [was *Rhizoglyphus robini* Claparede]	R1	Spalding, Lincolnshire, GB	Freesia Corms	16/8/71
*Suidasia pontifica* Oudemans [was *Suidasia medanensis* Oudemans]	S5	New Zealand	House Dust	May 1971
*Thyreophagus* sp. [was *Thyreophagus corticalis* (Michael)]	TH4	Climping, Littlehampton, West Sussex, GB	Sea bindweed (*Calyshigea soldsanella*)	25/9/79
*Thyreophagus entomophagus* (Laboulbene)	TH3	Micheldever, Hampshire, GB	Warehouse Dust	16/10/79
*Tyroborus lini* Oudemans	T66	GB	Poultry Farm	5/10/77
*Tyrolichus casei* Oudemans	T62	Slough, Berkshire, GB	Cheese	August 1951
*Tyrophagus brevicrinatus* Robertson	T89 or TB	Sierra Leone	Mouldy Palm Kernels	29/1/82
*Tyrophagus longior* (Gervais)	T40	Curly Hill, Aldershot, Hampshire	Mouse Nest	21/2/72
*Tyrophagus nieswanderi* Johnston and Bruce	T6	Wye, Kent, GB	Strawberries	1967
*Tyrophagus palmarum* Oudemans [’A’]	T17	Halford, Warwickshire, GB	Soil From Orchard	18/7/68
*Tyrophagus palmarum* Oudemans [B]	T32	Whitchurch, Shropshire, GB	Cheese Store	20/11/69
*Tyrophagus vanheurni* Oudemans [was *Tyrophagus palmarum* Oudemans [’C’]]	T7	Windsor Great Park, Berkshire, GB	Thrush Nest (*Turdus philomelus* or *Turdus viscivorus*)	1965
*Tyrophagus perniciosus* Zachvatkin [’A’]	T8	GB	Chaff Pile	*Recorded as*: ’Unknown’
*Tyrophagus perniciosus* Zachvatkin [B]	T38	Greenmore Hill Farm, Woodcote, Oxfordshire, GB	Barley Residue	31/3/71
*Tyrophagus putrescentiae* (Schrank) [’A’]	T13	South Africa	Groundnuts	23/7/68
*Tyrophagus putrescentiae* (Schrank) [B]	T9	Beaconsfield, Buckinghamshire, GB	Blue Tit’s Nest (*Parus caeruleus*)	1966
*Tyrophagus robertsonae* Lynch	T87	Lascelles Park, Slough, Berkshire	Bark	7/12/79
*Tyrophagus similis* Volgin [’A’]	T44	Lincolnshire, GB	Tomato Plant	4/7/72
*Tyrophagus similis* Volgin [B]	T21	Burnham Beeches, Buckinghamshire, GB	Beech Litter	May 1968
*Tyrophagus savasi* Lynch	T11	Old Trafford, Manchester, Cheshire, GB	Biscuits	1967
*Tyrophagus tropicus* Robertson	T90	Whampoa, China	Mouldy Manioc Slices	23/6/82
*Carpoglyphus lactis* (L.)	Ca4	Slough, Berkshire, GB	Sultanas	November 1955
*Chortoglyphus arcuatus* (Troupeau)	CH1	Burnham, Buckinghamshire, GB	Farm Residues	June 1974
*Glycycometus hughesae* (Fain) [was *Austroglycyphagus geniculatus* (Vitzthum)]	G3	Burnham, Buckinghamshire, GB	*Recorded as*: ’Unknown - maybe Disused Farm Buildings’	1972
*Lepidoglyphus destructor* (Schrank)	G6	Port Sunlight, Cheshire, GB	Residues	September 1951
*Glycyphagus domesticus* (De Geer)	G5	Barton-on-Humber, Humberside, GB	Maltings	November 1956
*Dermatophagoides farinae* Hughes	D4	Manchester, Cheshire	*Recorded as*: ’Unknown’	1962
*Dermatophagoides microceras* Griffiths and Cunnington	D5	Coppull Moor Farm, Coppull Moor, Lancashire	Residues and Poultry Feeding-stuffs	22/10/81
*Dermatophagoides pteronyssinus* (Trouessart)	D3	*Recorded as*: ’Unknown’	*Recorded as*: ’Unknown’	*Recorded as*: ’Unknown’
“Winterschmidtiidae sp.” [was *Calvolia* sp.]	CV1(66)	Silwood Park, Ascot, Berkshire, GB	Nesting Box	1966
*Forcellinia galleriella* Womersley	F1	Slough, Berkshire, GB	*Apis mellifera* Hive Debris	28/6/82

Capital letters in square brackets [...] represent isolated breeding groups (Griffiths 1979). Updated names from original collection identification [...] by Barry OConnor (pers.comm.)

Specimens in alcohol of the following samples were deposited with their origin data in the Museum of Zoology, University of Michigan, Ann Arbor, MI USA under accession number ITR-UMMZ-I-2020-018: *Acarus chaetoxysilos* AC204, *Cosmoglyphus* (was *Caloglyphus*) *oudemansi* C10, *Dermatophagoides microceras* D5, *Forcellinia galleriella* F1, *Glycyphagus domesticus* G8, *Thyreophagus entomophagus* TH3, *Tyrophagus robertsonae* T87, *Tyrophagus savasi* T11, and *Tyrophagus tropicus* T90.

Specimens in alcohol of the following samples were deposited with their origin data in the British Museum (Natural History), London UK under accession number AQ ZOO 2020-78: *Acarus chaetoxysilos* AC204, *Acarus gracilis* A4, *Acarus siro* [SW sp.] A15, *Aleuroglyphus ovatus* AL2, *Chortoglyphus arcuatus* CH1, *Cosmoglyphus* (was *Caloglyphus*) *oudemansi* C9, *Cosmoglyphus* (was *Caloglyphus*) *oudemansi* C10, *Dermatophagoides farinae* D4, *Dermatophagoides microceras* D5, *Dermatophagoides pteronyssinus* D3, *Forcellinia galleriella* F1, *Glycometus hughesae* (was *Austroglycyphagus geniculatus*) G3, *Glycyphagus domesticus* G5, *Glycyphagus domesticus* G8, *Lardoglyphus konoi* L1, *Lardoglyphus zacheri* L3, *Lepidoglyphus destructor* G6, *Lepidoglyphus destructor* G7, *Neosuidasia* sp. (was *Lackerbaueria* sp.) LA1, *Madaglyphus legendrei* (was *Comerinia chaetolamina*) T34, *Neocotyledon rhizoglyphoides* (was *Caloglyphus redikozevi*) C5, *Sancassania* (was *Caloglyphus*) *berlesei* C3, *Suidasia pontifica* (was *Suidasia medanensis*) S5, *Thyreophagus entomophagus* TH3, *Thyreophagus* sp. (was *Thyreophagus corticalis*) TH4, *Tyroborus lini* T66, *Tyrophagus perniciosus* [‘A’] T8, *Tyrophagus robertsonae* T87, *Tyrophagus savasi* T11, *Tyrophagus similis* [‘B’] T21, *Tyrophagus tropicus* T90, and ‘Winterschmidtiidae sp.’ (was *Calvolia* sp.).

Astigmatid habitats (grassland, storage, fruit, meat, cheese, dust, mattress, feathers, mammals, birds nests, bats, broiler) were taken from Hughes (1976). Other dimensions could have been chosen (e.g., Akimov and Oksentyuk 2018). Eleven mite species had ‘Unclassified’ habitats (*Acarus chaetoxysilos*, *Cosmoglyphus hugheseae*, “Winterschmidtiidae sp.”, *Forcellinia galleriella*, *Kuzinia laevis*, *Lackerbaueria* sp., *Neosuidasia* sp. *Tyrophagus savasi*, *Comerinia chaetolamina*, *Tyrophagus robertsonae*, and *Thyreophagus corticalis*). Each species was subjectively scored as: ‘soft food eater/not soft food eater’, ‘hard food eater/not hard food eater’, ‘generalist’/’specialist’ (mindful of Hughes 1976’s views on certain taxa), ‘clear habitat attribution/not clear habitat attribution’. Fifteen mite species had unclear habitat attribution (Winterschmidtiidae sp., *Forcellinia galleriella*, *Glycycometus hugheseae*, *Kuzinia laevis*, *Neosidasia* sp., *Tyrophagus savasi*, *Madaglyphus legendrei*, *Tyroborus lini*, *Tyrophagus robertsonae*, *Thyreophagus entomophagus*, and *Thyreophagus* sp.). The habitat for each mite species was also subjectively scored as: ‘houses/not houses’, ‘nidicolous/not nidicolous’. A positive score was given for any clear trophic attribution in Hughes (1976), a negative for the lack of such.

### Methods

Five morphological (*M*) attributes $x_{i,j,k}$ $(k=1\ldots 5)$ for the *i*th individual and *j*th species were digitised using a Summagraphics system from line drawings of mites (prepared by hand using Nomarski interference phase-contrast microscopy of specimens cleared in lactic acid and mounted in Heinz’s modified PVA). Bespoke computer programs converted those co-ordinates to $\mu \text {m}$ and synthesised block diagrams of the standardised designs for plotting. The *k* morphological (*M*) attributes were (Fig. 3):

- idiosomal index *IL* [$\rightarrow$ ‘size’ i.e., *s* herein],
- chelal lever height *L*1 (*L*1*U* adductive input moment arm herein),
- chelal moveable digit lever length *L*2 (*L*2*M* adductive output moment arm herein) [$\rightarrow$ ‘gape’],
- cheliceral height *CHI* ($\equiv PHI$ in Perdomo et al. 2012), and
- cheliceral length *CLI* [$\rightarrow$ ‘reach’].

The terms in the square brackets [...] reflects the mapping of *M* into ${\hat{D}}$. The number of chelal teeth was not examined, nor evidence of distinct occlusal regions (Brown et al. 1979) gathered. Fixed digit lengths and any of their contribution to possible stabbing adaptations (Adar et al. 2012) were not investigated. Measurements were initially not log transformed following Bookstein et al. (1985). Any relative sizing was done by simple arithmetic division of one measurement by another at the level of each individual specimen, scale-free standardisation of individuals (Stoddard 1979) was not used. The idiosomal index is the distance between the V-shaped part of the sternum *sensu* Hughes (1976) and the central point of a line drawn between the posterior margins of the last pair of mite trochanters (Fig. 3a). It is taken to be the ‘reference dimension’ (Brown and Davies 1972). The idiosomal index is less likely to be distorted during slide preparation than the (full) idiosomal length (Lynch 1989). It avoids the vagaries of trying to estimate body size by SEM photography. Taking *L*2*M* as gape (i.e., the maximum diameter of a food morsel that can be gripped) assumes a maximum opening angle of around $60{^{\circ }}$ for the chela in practice.

Each $k=1\ldots 5$ measure (*M*) was summarised for each of the $j=1\ldots 47$ species by a mean ($\mu _{j,k}$) for later heuristic modelling. The corresponding measures for $i=1$ to 20 individual ‘typical’ saprophagous astigmatid mites were simulated to form a central reference ‘anchor’ data set as: $x_{i,48,k}=\frac{1}{47}*\sum _{j=1}^{47}x_{i,j,k}$ $(i=1\ldots 20)$. This synthetic data was summarised in itself for each $k=1\ldots 5$ morphological measures and its 20 individuals by its mean ($\mu _{48,k}$) and its sample variance ($\sigma _{48,k}$). For this study $\mu _{48,k}\ :\ IL = 216.42, L1U = 12.66, L2M = 26.45, CHI = 51.59, CLI = 98.17$ $\mu$m and $\sigma _{48,k}\ :\ IL = 2.995, L1U = 0.238$, $\text {L2M} = 0.239, CHI = 0.621, CLI = 0.689\,\mu \text {m}$. This represents an arbitrary in silico sample of the overall ‘average acarine’ design as a notional reference group. This forms a compact basal form as the variance for this group over such averages forming each synthetic individual is smaller than that for each species on its own. It is acknowledged that the correlation pattern within this synthetic group does not quite exactly replicate (but is very close to) the average correlation pattern of each species. However, any bias in any SVD (singular value decomposition) including it is very small as it only contributes at most $100*\frac{1}{48} = 2.08\%$ of the overall standardised MCSSCP (mean corrected sums of squares and cross-products) matrix or generalised variance.

Two ways of estimating the potential closure static force (*F*1, Fig. 3b) for the chela of each individual were used (Bowman 2021). One assumes a pennate muscle morphology inside the cheliceral shaft, the other a radial one. That is: a pennate assumption $F1P=\frac{CHI}{2}*(CLI-(1.1*L2M))$ ignoring the angle of muscle fibres to the adductive tendon, or a radial assumption $F1C=\pi *(\frac{CHI}{2})^2$. The 1.1 inflation factor in *F*1*P* was a measured average in preliminary investigations (*not shown*) of where the condyle location with respect to total length of the moveable digit inside the cheliceral base shortens the effective space for the muscle mass. The division by two in *F*1*C* is to allow room in the cheliceral base for the chelal opening abductor muscle below the chelal closing adductor muscle (unlike anactinotrichid mites there is no extra basal cheliceral segment in astigmatids). The *F*1 approximations were well correlated between each other ($R^2=0.8825$). *F*1*P* correlated almost exactly with *L*2*M*. *F*1*C* correlated almost exactly with *CHI*. The potential final nominal biting or ‘crunch force’ on any foodstuff was estimated by multiplication of the *F*1 estimates with the velocity ratio estimate (= mechanical advantage of a frictionless chelal lever Alexander (1983), where $VR = L1U/L2M$), to yield *F*2*P* and *F*2*C*, respectively, at the individual mite specimen level. These were even more well correlated with each other ($R^2=0.9105$; Fig. 4). So finally, simply averaging over the two topological assumptions yielded the consensus estimate *F*2*AV*. The consensus estimate was an attempt at being hypothesis-free regarding muscular origin, so as to allow wide comparability to other animals, rather than only accepting Perdomo et al. (2012)’s assumptions. Evidence to support this approach is given in Bowman (2021). Note that $\mu _{48,k}=926.53\, \mu \text {m}^2$ and $\sigma _{48,k}=33.228\,\mu \text {m}^2$ for the *F*2*AV* of the central reference ’anchor’ mite set. Relative crunch force measures were calculated by simple division before summary where necessary.

**Table 2:** Mean and standard deviation ($\mu _{j}$ : $\sigma _{j}$) values by astigmatid species reviewed ($j=1\ldots 48$)

Species	Culture number	Size ($$\mu \text {m}$$)	Reach ($$\mu \text {m}$$)	Gape ($$\mu \text {m}$$)	Velocity ratio ($$\mu \text {m}$$)	Crunch force $$(\mu m^{2})$$	*CHI* ($$\mu \text {m}$$)	*L*1*U* ($$\mu \text {m}$$)
*Acarus chaetoxysilos*	AC204	185.87 : 10.93	80.25 : 5.02	20.61 : 1.52	0.463 : 0.051	507.15 : 85.08	37.50 : 2.37	9.51 : 1.03
*Acarus farris*	A17	200.05 : 20.21	82.22 : 3.36	22.68 : 1.61	0.400 : 0.042	465.01 : 94.00	38.95 : 3.94	9.07 : 1.12
*Acarus gracilis*	A4	192.04 : 16.49	94.88 : 13.16	24.29 : 1.69	0.476 : 0.065	800.60 : 207.22	46.80 : 5.06	11.52 : 1.44
*Acarus immobilis*	A1	225.81 : 17.24	89.05 : 3.53	23.52 : 1.54	0.444 : 0.060	621.88 : 135.49	42.67 : 3.29	10.39 : 1.23
*Acarus siro* [’H’]	A10b	221.44 : 13.56	108.33 : 2.82	30.34 : 2.01	0.476 : 0.053	1167.48 : 194.78	58.48 : 3.32	14.40 : 1.34
*Acarus siro* [SW sp.]	A15	203.20 : 13.57	101.07 : 4.97	27.68 : 2.28	0.456 : 0.032	828.98 : 135.99	49.00 : 3.82	12.62 : 1.38
*Aleuroglyphus ovatus*	AL2	211.17 : 10.46	116.94 : 5.84	34.00 : 1.98	0.538 : 0.048	1839.18 : 327.53	71.03 : 5.74	18.24 : 1.51
*Sancassania berlesei*	C3	260.65 : 25.89	110.01 : 7.62	27.32 : 2.38	0.429 : 0.048	959.95 : 167.19	54.03 : 4.13	11.68 : 1.31
*Cosmoglyphus oudemansi*	C10	219.75 : 21.98	92.90 : 4.70	24.45 : 1.01	0.426 : 0.061	700.86 : 171.41	46.66 : 4.04	10.38 : 1.32
*Cosmoglyphus hughesae*	C5	205.87 : 15.44	93.14 : 5.37	24.60 : 2.36	0.427 : 0.047	735.12 : 140.19	48.26 : 4.20	10.45 : 1.16
*Kuzinia laevis*	KL or $	331.13 : 62.25	146.53 : 7.00	46.13 : 3.00	0.465 : 0.036	2544.79 : 484.11	90.90 : 7.66	21.45 : 2.18
*Lardoglyphus konoi*	L1	204.83 : 31.44	88.62 : 6.18	19.68 : 2.45	0.523 : 0.089	775.59 : 153.56	43.69 : 3.23	10.11 : 0.90
*Lardoglyphus zacheri*	L3	239.59 : 16.39	98.20 : 5.12	23.49 : 1.84	0.476 : 0.078	763.77 : 213.33	44.34 : 3.66	11.12 : 1.61
*Neosuidasia* sp.	LA1	214.34 : 24.10	140.36 : 8.46	37.38 : 3.00	0.610 : 0.079	2615.28 : 695.19	76.67 : 8.63	22.73 : 2.68
*Madaglyphus legendrei*	T34	182.49 : 10.25	85.44 : 3.65	20.99 : 1.43	0.475 : 0.047	674.33 : 118.13	43.29 : 3.20	9.96 : 1.17
*Rhizoglyphus echinopus*	R2	287.59 : 30.97	119.82 : 4.88	31.82 : 2.40	0.448 : 0.052	1273.05 : 233.60	61.96 : 4.25	14.26 : 1.80
*Rhizoglyphus echinopus*	R1	304.24 : 17.56	123.29 : 5.86	33.84 : 2.12	0.461 : 0.041	1457.52 : 235.49	66.15 : 4.50	15.62 : 1.75
*Suidasia pontifica*	S5	208.38 : 25.59	81.55 : 2.76	20.46 : 1.84	0.534 : 0.061	822.56 : 157.10	46.35 : 2.67	10.85 : 0.96
*Thyreophagus* sp.	TH4	356.20 : 18.91	104.65 : 4.29	27.45 : 1.84	0.525 : 0.059	1300.03 : 224.42	58.99 : 4.36	14.33 : 1.00
*Thyreophagus entomophagus*	TH3	234.65 : 14.58	94.01 : 4.10	24.50 : 1.71	0.509 : 0.064	1056.24 : 232.25	54.07 : 4.32	12.41 : 1.38
*Tyroborus lini*	T66	231.93 : 17.37	118.98 : 4.43	33.20 : 1.90	0.539 : 0.040	1797.54 : 352.10	69.17 : 6.56	17.90 : 1.75
*Tyrolichus casei*	T62	260.31 : 14.70	118.75 : 5.18	31.88 : 1.76	0.461 : 0.042	1130.65 : 144.81	56.70 : 2.70	14.66 : 1.25
*Tyrophagus brevicrinatus*	T89 or TB	270.09 : 14.60	94.01 : 4.24	25.31 : 1.73	0.442 : 0.042	886.84 : 186.19	53.04 : 4.47	11.19 : 1.32
*Tyrophagus longior*	T40	232.52 : 12.68	100.73 : 4.51	25.93 : 1.78	0.449 : 0.043	950.30 : 129.78	53.86 : 2.93	11.61 : 1.00
*Tyrophagus nieswanderi*	T6	211.78 : 18.14	95.84 : 3.71	23.97 : 1.04	0.473 : 0.054	803.17 : 155.17	47.00 : 3.58	11.31 : 1.16
*Tyrophagus palmarum* [’A’]	T17	216.86 : 27.40	92.41 : 3.74	22.16 : 1.16	0.416 : 0.054	610.45 : 146.94	42.78 : 4.32	9.23 : 1.30
*Tyrophagus palmarum* [’B’]	T32	203.79 : 12.60	94.29 : 2.64	23.56 : 1.30	0.460 : 0.058	761.44 : 182.31	46.34 : 3.77	10.81 : 1.30
*Tyrophagus vanheurni*	T7	227.36 : 14.03	100.62 : 4.43	26.26 : 1.75	0.431 : 0.052	727.12 : 127.71	46.40 : 2.54	11.32 : 1.63
*Tyrophagus perniciosus* [’A’]	T8	228.90 : 18.89	106.47 : 5.26	28.03 : 1.33	0.426 : 0.067	785.51 : 170.74	48.22 : 3.44	11.94 : 1.92
*Tyrophagus perniciosus* [’B’]	T38	237.55 : 15.43	115.38 : 4.87	31.02 : 2.12	0.487 : 0.057	1160.39 : 199.89	56.08 : 3.34	15.05 : 1.66
*Tyrophagus putrescentiae* [’A’]	T13	188.49 : 35.24	91.42 : 3.82	23.80 : 1.37	0.411 : 0.060	594.26 : 123.85	43.16 : 3.65	9.78 : 1.57
*Tyrophagus putrescentiae* [’B’]	T9	210.92 : 16.11	91.41 : 5.60	23.95 : 1.38	0.473 : 0.062	696.27 : 179.11	43.58 : 3.64	11.33 : 1.62
*Tyrophagus robertsonae*	T87	172.11 : 12.31	77.51 : 5.45	20.08 : 1.91	0.496 : 0.052	503.33 : 58.94	36.21 : 2.31	9.88 : 0.55
*Tyrophagus similis* [’A’]	T44	211.12 : 16.16	96.58 : 5.06	24.88 : 1.45	0.420 : 0.040	660.23 : 89.11	44.92 : 2.98	10.44 : 1.02
*Tyrophagus similis* [’B’]	T21	219.84 : 16.11	95.70 : 3.90	24.81 : 1.29	0.451 : 0.040	765.52 : 125.16	47.33 : 3.14	11.17 : 1.01
*Tyrophagus savasi*	T11	194.72 : 16.04	96.72 : 5.01	24.24 : 1.45	0.466 : 0.040	751.60 : 121.20	45.40 : 3.18	11.29 : 0.99
*Tyrophagus tropicus*	T90	211.80 : 12.71	86.82 : 3.78	21.70 : 1.64	0.509 : 0.053	755.77 : 139.38	44.49 : 4.47	11.01 : 1.09
*Carpoglyphus lactis*	Ca4	202.63 : 15.11	81.05 : 2.71	25.30 : 1.67	0.355 : 0.047	302.25 : 57.23	32.51 : 2.47	8.94 : 1.03
*Chortoglyphus arcuatus*	CH1	200.36 : 7.29	104.55 : 4.84	32.94 : 2.97	0.582 : 0.063	1508.53 : 214.41	62.25 : 2.58	19.06 : 1.63
*Glycycometus hughesae*	G3	171.77 : 24.43	108.90 : 4.33	31.82 : 1.94	0.503 : 0.062	1452.69 : 201.87	65.34 : 2.97	15.96 : 1.71
*Lepidoglyphus destructor*	G6	225.53 : 55.54	102.03 : 3.46	30.10 : 1.49	0.484 : 0.064	1522.37 : 372.91	69.45 : 7.13	14.52 : 1.78
*Glycyphagus domesticus*	G5	186.67 : 35.58	105.44 : 6.23	28.88 : 2.31	0.456 : 0.048	1094.29 : 213.23	57.86 : 5.13	13.18 : 1.70
*Dermatophagoides farinae*	D4	151.58 : 7.57	87.66 : 5.92	29.36 : 3.01	0.514 : 0.115	1317.21 : 452.97	64.21 : 6.20	14.94 : 2.78
*Dermatophagoides microceras*	D5	119.60 : 6.67	76.63 : 2.89	22.99 : 1.96	0.715 : 0.118	923.62 : 205.77	43.11 : 3.11	16.27 : 1.81
*Dermatophagoides pteronyssinus*	D3	123.52 : 8.52	73.49 : 4.18	22.59 : 3.20	0.568 : 0.114	852.17 : 270.30	47.72 : 3.69	12.61 : 1.59
“Winterschmidtiidae sp.”	CV1(66)	177.16 : 11.57	65.16 : 3.70	17.14 : 1.18	0.442 : 0.052	354.13 : 56.38	32.73 : 2.00	7.55 : 0.88
*Forcellinia galleriella*	F1	193.56 : 22.07	84.08 : 2.64	22.18 : 1.25	0.486 : 0.052	717.30 : 97.11	45.12 : 2.30	10.74 : 0.89
Typical	–	216.42 : 2.99	98.17 : 0.69	26.45 : 0.24	0.478 : 0.009	926.53 : 33.23	51.59 : 0.62	12.66 : 0.24

Capital letters in square brackets [...] represent isolated breeding groups (Griffiths 1979)

The resultant design (*D*) attributes thus were

- Size = *s*
- Gape = *L*2*M*
- Reach = *CLI*
- Crunch force = *F*2*AV*

All data collected, generated and analysed during this study and all new data generated or analysed, plus any model specifications are included in this published article or in compliance with EPSRC’s open access initiative are available from https://doi.org/10.5287/bodleian:9RxgYr4Jm.

### Ordinations

Ordinations in the radial observed information space each individual of each species gives to the morphological or design distinction of their form from the typical mite form or design was carried out using the methods of Bowman (2015a). This is an optimal display of the data radially from the origin in the space of the information that each individual has for the question of interest. The question of interest was:

What does each mite individual of each of these species contribute to the difference between themselves as a species and the typical mite (in terms of their morphological design)?

This is probed by, firstly, calculating a mean and sample variance for each of the $j=1 \ldots 47$ species as a set over the six trophic morphology (*M*) and design (*D*) measures (*IL*, *L*1*U*, *L*2*M* , *CHI*, *CLI*, the crunch force *F*2*AV*) as

$$\begin{aligned} \mu _{j,k}= & {} \frac{1}{20}*\sum _{i=1}^{20}x_{i,j,k} \quad (k=1 \ldots 6)\\ \sigma _{j,k}^{2}= & {} \frac{1}{(20)-1}*\sum _{i=1}^{20}(x_{i,j,k}-\mu _{j,k})^2 \quad (k=1 \ldots 6) \end{aligned}$$

then taking the synthetic data

$$\begin{aligned} x_{i,48,k}=\frac{1}{47}*\sum _{j=1}^{47}x_{i,j,k}\quad (i=1 \ldots 20,\ k=1 \ldots 6) \end{aligned}$$

forming its mean

$$\begin{aligned} \mu _{48,k}=\frac{1}{20}*\sum _{i=1}^{20}x_{i,48,k} \quad (k=1 \ldots 6)\quad \equiv \frac{1}{47}\sum _{j=1}^{47}\mu _{j,k} \end{aligned}$$

and its sample variance

$$\begin{aligned} \sigma _{48,k}^{2}=\frac{1}{(20)-1}*\sum _{i=1}^{20}(x_{i,48,k}-\mu _{48,k})^2 \quad (k=1 \ldots 6) \end{aligned}$$

Then, for each individual $(i=1 \ldots 20)$ of each species $(j=1 \ldots 48)$ and each morphological or design measure $x_{i,j,k}$ $(k=1 \ldots 6)$ separately, the individualised log likelihood ratio (‘observed divergence’) was calculated using the quadratic discriminant equation

1 $$\begin{aligned} lbf_{i,j,k}=ln(\sigma _{j,k})+\frac{(x_{i,j,k}-\mu _{j,k})^2}{2*\sigma ^{2}_{j,k}} -ln(\sigma _{48,k})-\frac{(x_{i,j,k}-\mu _{48,k})^2}{2*\sigma ^{2}_{48,k}} \end{aligned}$$

where the 48th set is the central synthetic typical mite set of individuals. This log Bayes factor $(lbf_{i,j,k})$ measures (as a ‘weight of evidence’), the observed *directed* difference (or divergence) that an individual measured $x_{i,j,k}$ instance gives to the distinction between the likelihood space of that individual’s species’ morphology *M* (and design *D*) versus the likelihood space of assuming the reference typical mite morphology *M* (and design *D*). It evaluates as zero on average for the simulated reference data set of ‘typical’ individuals. This process forms an objective contrast of multivariate position versus an overall average reference, or in other words a comparison of individual mite morphological models to a central ‘yardstick’ in *directed* multidimensional space.

These marginal values of $lbf_{i,j,k}$ then replace the data values $x_{i,j,k}$. This transforms the morphological or design data-matrix into an information space matrix of the evidence that the *i*th instantiation of the *j*th species and *k*th measure gives to the distinction that each individual mite’s morphology or trophic design as a species shows compared to that of a typical astigmatid mite. Replacement of the raw data with the corresponding *lbf* value preserves but rescales the original morphological or design co-occurrence (i.e., the covariation structure; Bowman 2009). In this way, observed information of distinction values (or ‘weight of evidence’ values; Sharma 2011) were calculated for each observation of each measure for each mite as above and used as data replacements. Each new data column was then standardised, dummy indicator [0, 1] species variables added if appropriate as new columns and the observed MCSSCP matrix over all the new data calculated. Standardisation ensures each variable contributes an equal amount of variance to the result (i.e., it is an a priori equipoise assumption). This augmented correlation matrix was decomposed to its eigenvalues snd eigenvectors using R.

Singular value decomposition of the correlation matrix of these *lbf* measures yields the important orthogonal sets of latent self-correlated structures, ‘components’ here defining mite morphology or trophic design in general. So the whole process is a non-linear data transformation (data$\rightarrow$individualised divergence), plus three affine procedures: a rotation, a shear and a compression/dilation of the original space. Adding dummy [0,1] variables for each species ensures that the simulated typical mite morphology or design is located *centrally* in a positive space display of individuals with the *direction* of other species arranged optimally radially around it as a stellation. The decomposition of evidence is thus borrowed across all of the species. The direction of the morphological or design characters on biplots indicates how changes in these are spread over all of the species. Each taxon can be summarised by a distance and an angle (‘North’ vertically up the page, ‘East’ is to the right of the page, etc.) from the origin ($\equiv$ location of the typical reference mite). Angle is thus a circular measure (see Cremers and Klugkist 2018). Hypothesis tests concerning groupings of species uses: for distances Welch’s *t*-test, and for angles the Large-sample Mardia–Watson–Wheeler test for a common distribution (two samples) confirmed with the Randomization version of Mardia–Watson–Wheeler test, in R version 4.0.2 (2020-06-22) (see Pewsey et al. 2014). The individualised divergences ordination method used avoids the arbitrary nature of the metric and comparisons in a typical PCA. It also avoids the explicit minimisation of between and within species variation within any canonical correlation analysis deforming the display.

The six terms in the final morphology (*M*) and design (*D*) ordination were the primary morphological and design measures, and the species assignation indicators (0, 1). This supervised method is ‘blind’ to what the species actually are and their biology but acarines of similar morphology or trophic design will be found close to each other in such displays, characters grading across this optimal geometric arrangement. The indicator variables give the direction and location of each species’ average position in the morphology or design distinction space. Each specimen of each mite was plotted on the first two principal components of the ordinations and GRAPHIS 2.9 used to overlay taxonomic or ecological contours over the ordination as observed heat-maps. PLS (partial least squares) used least squares multiple regression in Excel or R to fit gradients to these observed contours if required. For heat-map displays the compass directions are also used, ‘North’ is taken to be up the page, ‘South’ down the page, ‘East’ is to the right of the page, and ‘West’ to the left.

### Heuristics

Heuristics is a first-step methodology for devising a taxonomy. They segment phenomena into groupings at first subjectively into a possible ‘story’, but then when confirmed by statistical examination generate objective testable hypotheses. ‘Four-box’ heuristic models are a dichotomous graphical display of measures cross-classified as ‘high’ or ‘low’ for each of two axes. They are an exploratory or descriptive tool used to understand ‘landscapes’ irrespective of their homogeneity. These are widely used in applied research and commerce, for instance recursive partitioning (CART) is a similar type of repeated binary division model. The division for each axis or ‘cut boundary’ herein was defined as the value of a parameter measured above or below that of the ‘typical’ average astigmatid mite ($\mu _{48,k}$) to yield four quadrants. Into each of these heuristic boxes, the species were placed appropriately depending upon their average parameter value ($\mu _{j,k}$) $(j=1 \ldots 47)$. In this way each mite’s ecological adaptations and functions are categorised into a discrete set of ecomorphologies that eschews needing to find intermediates (Tseng and Stynder 2011). Models over the design space (*D*) were chosen combining the $k=1\ldots 5$ morphological measures with the $k=6$ crunch force in a biologically rational way rather than just all possible blind combinations. The advantage of this approach is that departures of trophic design in any direction can be clearly seen and related back to the multivariate ordination. No probabilistic conclusion is involved initially so statistical test multiplicities are avoided.

### Statistics

Body size ratios were tested using EcoSimR in R v 4.0.2 (2020-06-22) following Simberloff and Boecklen (1981). Four metrics are deployed for statistical analysis. Prior to calculation of these metrics, the body sizes (or trait values) are ordered from smallest to largest. The default algorithm of simulating a uniform distribution of body sizes within the limits defined by the largest and smallest species in the assemblage was used. ‘Variance ratio’ calculates the variance in the size ratios of consecutively ordered body sizes. Ratios are always calculated as (larger/next larger), so they must be $\ge 1$. If this variance is unusually small, there is evidence of constancy in size ratios for the assemblage. In the extreme case, if the size ratio between adjacent species is a constant, the variance in these ratios will be zero. ‘Variance difference’ calculates the variance of the absolute size differences between adjacent species. A small variance difference indicates a regular spacing of observations. In the extreme case, if the spacing between adjacent species is a constant, the variance in these differences will be zero. Note that if variance difference is very small, the variance ratio will not be, and vice-versa. This metric was introduced by Poole and Rathcke (1979) to test for regular spacing of flowering phenologies. ‘Minimum ratio’ calculates the minimum size ratio between adjacent pairs of species. If there are ties in the data, then minimum ratio will equal one. ‘Minimum difference’ calculates the absolute minimum size difference between adjacent pairs of species. If there are ties in the data, then minimum difference will equal 0.0.

### Geometric morphometrics

Geometric morphometrics followed Bookstein (2018) using MorphoJ version 1.06d software (Klingenberg 2011). Nine fixed cheliceral and chelal landmarks (Fig. 3c) were used ($1 =$ moveable digit condyle, $2 =$ adductive tendon junction with upper moment lever arm *L*1*U*, $3 =$ depressive tendon junction with lower moment lever arm *L*1*L*, $4 =$ distal tip of moveable digit, $5 =$ distal tip of fixed digit, $6 =$ lyrifissure approximately dorsal of condyle, $7 =$ dorsal extent of cheliceral shaft at posterior of distal segment (used for *CHI*), $8 =$ furthest proximal extent of cheliceral shaft (used for *CLI*), $9 =$ ventral extent of cheliceral shaft at posterior of distal segment (used for *CHI*). Procrustes fits within each species (with a full set of landmarks) were calculated. Procrustes co-ordinates reflect scale-free shape. The average (over individual mites) Procrustes coordinates were calculated for each taxon and these combined into a consensus dataset. This in turn underwent a Procrustes fit, then transformation vectors and transformation grids were estimated. Each taxon was then plotted within a principal component analysis of the covariance matrix of these final Procrustes co-ordinates. Comparisons of within species transformation grid vectors were made as appropriate.

## Results

The mean and sample SD for each species and measurement are shown in Table 2. In this review, large size (*s*) is taken to indicate a likely surface habit, small size the opportunity to be interstitial in behaviour. Similarly, large gape is taken to indicate the ability to deal with large food morsels and small gape to being restricted to grasping small food items. Ratios of body sizes are shown in Tables 3 and 4. All chelae appeared to be chelate suitable for grasping food, no clear special stabbing or holding adaptations like those in Nematalycidae (Bolton et al. 2015) were seen.

**Table 3:**
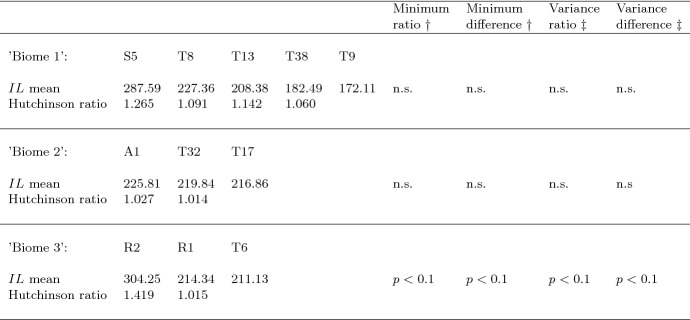
Hutchinson ratio tests of size as an approximate first-pass filter of adaptive syndromes († = ‘Test 1, ‡ = ‘Test 2’. See text) Species codes as in Table 1. Ordered mean idiosomal index (*IL*) for each species in 3 common ‘biomes’ as defined by a cross-classification of habitats in Hughes (1976). Increase on stepping-up the linear body sizes for each species sometimes agrees with expected ratio of 1.1-1.4 for sympatric congeneric species (i.e., in 6 out of $9 = 66\%$ of comparisons). Biome 1 is ‘Cheese & Storage habitat, Not-nidicolous Not-Houses Soft food Specialists’ biome. Biome 2 is ‘Grassland & Cheese, Birds-nest Nidicolous (but) Not-Houses Soft food Specialists’ biome. Biome 3 is ‘Grassland, Not-Houses Not-Nidicolous Soft food Specialists’ biome. For details of statistical tests ex Simberloff and Boecklen (1981) and Poole and Rathcke (1979) see Materials and Methods. Only for Biome 3 is a random allocation of body sizes rejected

**Table 4:**
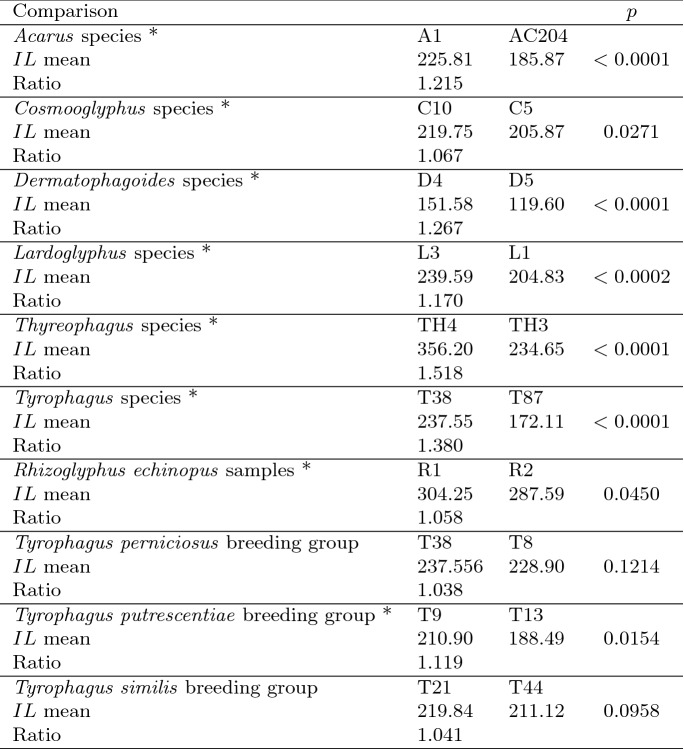
Calculation of ‘Hutchinson ratios of size’ is illuminating taxonomically. Statistical comparison uses Welch’s *t*-test. Species codes as in Table 1. Ordered mean idiosomal index (*IL*) for each largest and smallest ‘species in genera’, ’breeding group in species’ defined by Tables 1 and ‘samples within species’ sorted alphabetically. *Result of interest. Increase on stepping-up the linear body sizes (*s*) from smallest to largest within each genus or species frequently agrees with ratio of 1.1–1.4, i.e., approximate doubling of body weight from smallest taxon to largest taxon

### What does trophic morphology (*M*) say about astigmatid mechanical design (${\hat{D}}$)?

Mite measurements (excepting idiosomal length) are broadly correlated with each other (Fig. 4). The largest idiosomal index seen was for *Thyreophagus* sp. (TH4), the smallest for *Dermatophagoides farinae* (D4). The largest reach seen was for *Kuzinia laevis* (KL), the smallest reach for *Dermatophagoides pteronyssinus* (D3). The largest gape was seen for *Kuzinia laevis* (KL), however, there is no suggestion that it feeds by a closed chela stabbing into foodstuff. Rather *Kuzinia laevis* naturally feeds on pollen grains in *Bombus* spp. nests (OConnor *pers. comm.*), so a large gape might be expected. The smallest gape was seen for “Winterschmidtiidae sp.”. The highest velocity ratio (*VR*) was seen for *Dermatophagoides microceras* (D5), the lowest for *Carpoglyphus lactis* (Ca4). The largest consensus crunch force (*F*2*AV*) seen was for *Neosuidasia* sp. (LA1), the smallest crunch force for *Carpoglyphus lactis* (Ca4). The crunch-force (*F*2*AV*) is a little skew i.e., dominated by the larger mite species in the whole sample of 47 species reviewed. Akimov and Gaichenko (1976) found that the estimated force at the tip of the moveable digit between astigmatids ranked them as: *Chortoglyphus arcuatus* > *Acarus siro* > *Kuzinia laevis* > *Carpoglyphus lactis* by a different method. If *Kuzinia laevis* is omitted, this review finds the same relative ranking.

**Fig. 5:**
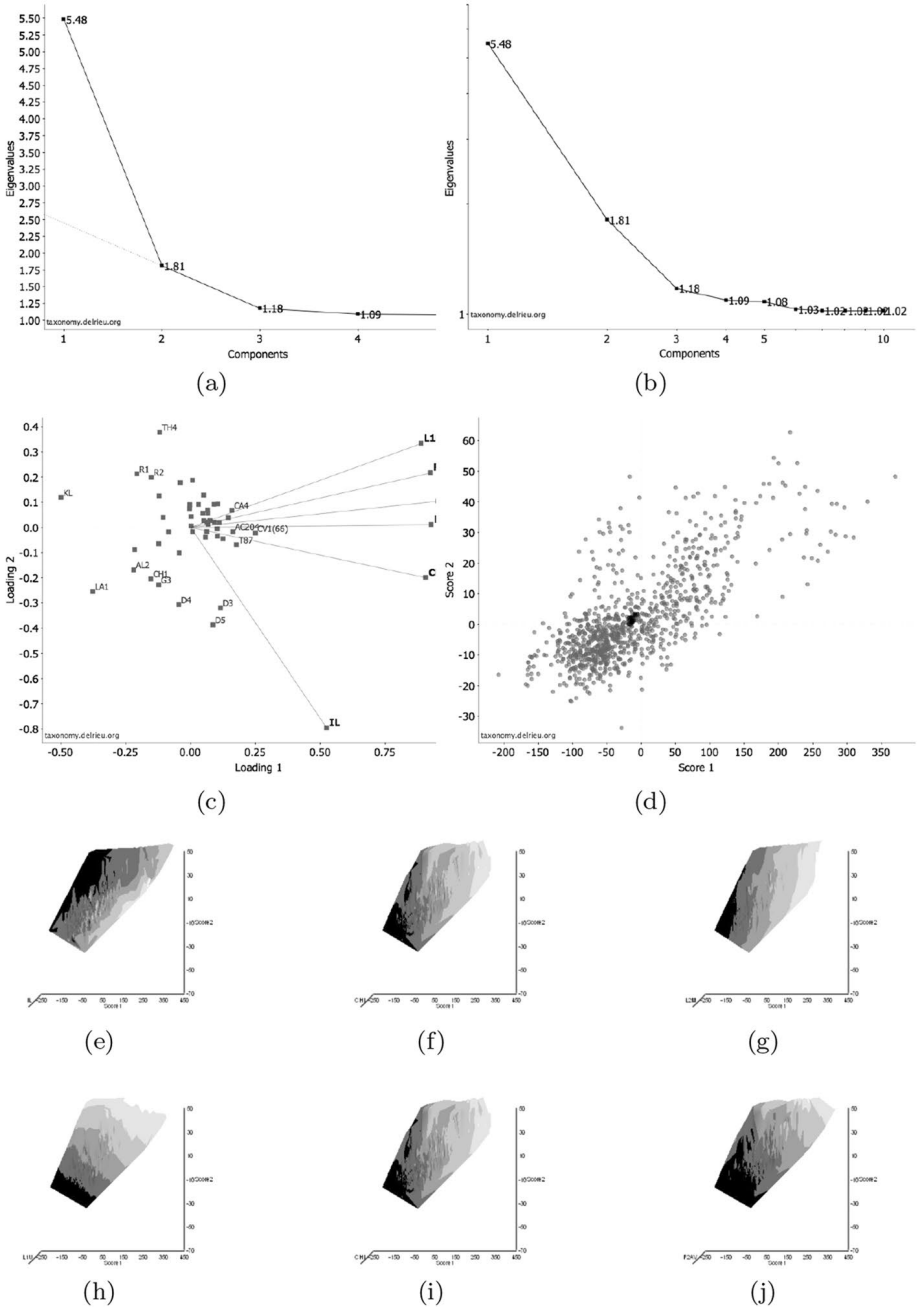
Ordination in the radial information space of size (*IL*), reach (*CLI*), gape (*L*2*M*), *L*1*U*, *CHI* and crunch force (*F*2*AV*) over reviewed astigmatid species versus nominal average trophic design. Some species labels have been omitted for clarity. Loadings plot directions match the heat-maps gradient directions. Permutation *p*-values for ordination: *IL* (0.345), *L*1*U* (0.367), *L*2*M* (0.351), *CHI* (0.302), *CLI* (0.285), *F*2*AV* (0.299). All n.s. indicating compact generality of astigmatid design. Length measures, height measures and crunch force heat-maps over same ordination scores (darker $=$ lower, paler $=$ higher, ‘North’ vertical on page). **a** Eigenvalue scree-plot of *lbf* correlation matrix. **b** Log–log eigenvalue scree-plot of *lbf* correlation matrix. Note only two latent components or important self-correlated sets of variables. **c** Loadings plot including dummy variables for each species. Note one dominant vector broadly equi-weighted on all measures (i.e., scale) and in particular equi-weighted on cheliceral measures (i.e., cheliceral scale), the other orthogonal subsidiary vector contrasting length measures (*IL*, *CLI*, *L*2*M*) with height measures (*L*1*U*, *CHI*) + crunch force (*F*2*AV*) $\equiv$ aspect ratio change. **d** Scores plot for each individual mite—orientation as in heat-maps. Simulated ‘typical’ mite design individuals in black (centrally). Note approximate quadrilateral envelope for ‘cloud’ of individuals sampled as per heatmaps. **e** Idiosomal length *IL* (‘NorthWest-SouthEast’ gradient). **f** Reach *CLI* (‘NorthWest-SouthEast’ + ‘West-East’ gradient). **g** Gape *L*2*M* (‘West-East’ gradient). **h** *L*1*U* (‘SouthWest-NorthEast’ gradient). **i** *CHI* (‘West-East’ gradient). **j** Crunch force *F*2*AV* (‘SouthWest-NorthEast’ gradient)

An ordination of species in terms of absolute size (*IL*), reach (*CLI*), gape (*L*2*M*), *L*1*U*, *CHI* and crunch force (*F*2*AV*) is shown in Fig. 5. From this (in the sub-figure (d)) a good coverage of each morphological measure over all species and specimens, and a clear central base point for the average typical mite is clear. This set of 47 species can be summarised by just two dimensions (scree plots in Fig. 5a–b). The first two principal components explain the bulk of the variation in information (cf. scree-plot for the ordination). Component 1 is dominated by chelal and cheliceral measures, component 2 is dominated by the idiosomal index (Fig. 5c). Heat-maps of each measurement over this are also shown (Fig. 5e–j).

Astigmatid mites in general thus appear to be ‘shrinkings/swellings’ of each other in size (*s*) together with some cheliceral differentiation. Examining the two important components in Fig 5c in detail shows that: one dominant vector broadly equi-weights on all measures (i.e., general mite scale) and in particular equi-weights on cheliceral measures (i.e., cheliceral scale), the other subsidiary vector contrasts (in sign) the length measures (*IL*, *CLI*, *L*2*M*) with the height measures (*L*1*U*, *CHI*) together with the chelal adductive crunch force (*F*2*AV*) indicating a change in aspect ratio. In the heat-maps (sub-figures (e)–(j)), size grades differently to the other measures. Reach, gape, cheliceral height and crunch force *F*2*AV* all grade broadly ‘West-East’, input moment arm *L*1*U* grades broadly ‘SouthWest-Northeast’ (Fig. 5; darker = lower, paler = higher). This is all congruent with the general correlation of measurements across species (Fig. 4) confirmed by the approximate right angle between the *IL* vector in Fig. 5c with all other measures. In other words, most astigmatid mites examined are uniform proportionate ‘swellings in size’ of other mites (i.e., they are not markedly allometric). Mite species to the ‘South-East’ have a larger body size and generally a longer reach, those to the ‘North-West’ a smaller body size and generally a smaller reach. The extra gnathosomal differentiation being mite species to the ‘West’ have a smaller gape and less tall chelicerae, mite species to the ‘East’ a larger gape and more tall chelicerae. Mite species to the ‘South-West’ have a weaker crunch force and a less tall chelal input moment lever arm *L*1*U* than those to the ‘North-East’ who have a stronger crunch force and a larger chelal lever arm *L*1*U*. An aspect ratio change is thus present over the 47 species reviewed.

**Fig. 6:**
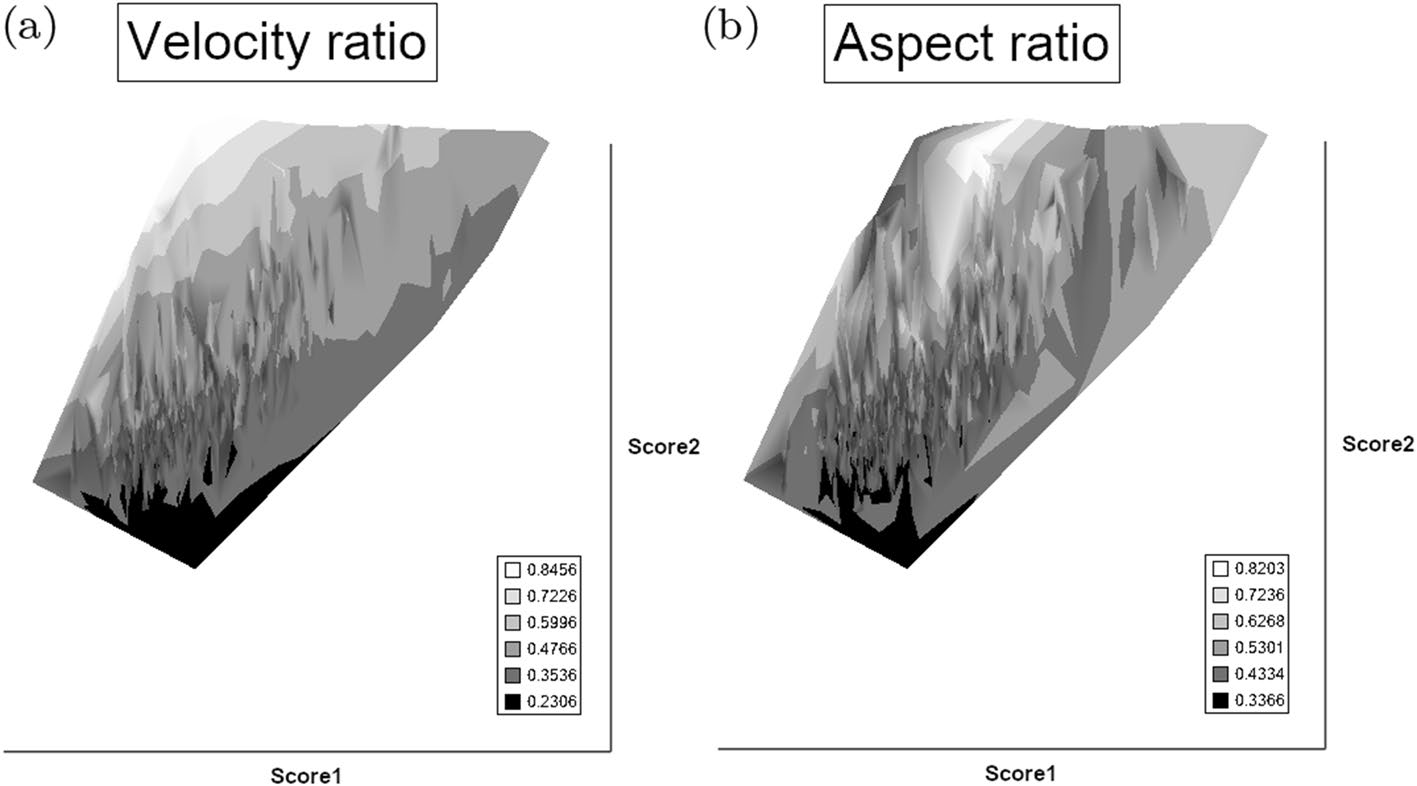
Heat-map over ordination of reviewed astigmatid species (darker $=$ lower, paler $=$ higher, ‘North’ vertical on page) for **a** Velocity ratio ($VR=\frac{L1U}{L2M}=VR$) showing approximately ‘South-NorthWest’ gradient—much like inverse of that for *IL* (Fig. 5). Low values pertain to a fast ‘snap’ closing, cutting (or picking) design like scissors or ‘tweezers’. High values pertain to a slow closing, crushing design like pliers (Alexander 1983). Least square smooth fitted surface ($\equiv$ actual PLS, *not shown*) gives ratio of slope for overlain *VR* data of Score1 to Score2 $= -10.3$. **b** Aspect ratio (*CHI*/*CLI*). No clear pattern of Aspect ratio except at extreme ‘North’ (white $=$ high) and ‘South’ (dark $=$ low)

From the heat maps in Fig. 5e, mites in the ‘North-West’ (where the pyroglyphids sit) are surprisingly small given that most measures increase to the ‘NorthEast’. The ‘North-South’ direction represents a trend for switching from cheliceral and chelal design proportionately more dominated by vertically measured features (*CHI*, *L*1*U*) to a design proportionately more dominated by horizontally measured features (*CLI*, *L*2*M*), i.e., a cheliceral shortening/elongation axis. This in itself is not well correlated with the idiosomal length (*IL*). So, smaller mites usually have more elongate, shallower chelicerae and shallower chelae (excepting *Dermatophagoides* spp.). Larger mites usually have taller chelicerae and taller chelae. However, an overlaying extra change in heights versus lengths is present too (Fig. 6b). This is consilient with the (inverse) pattern for the velocity ratio trend (i.e., the moveable digit adducting lever mechanical advantage in an ideal friction-less right-angled system, Fig. 6a). There appears to be an extra ‘elongation/shortening’ motif overlaying the ‘shrinking/swelling’ general design of astigmatid mites. Equivalently, larger idiosomal index mite species are more elongate mites in general compared to their chelicerae. Smaller idiosomal index species are more squat mites in general compared to their chelicerae. In oribatids, data in the Tables and Figures of Schuster (1956) and Kaneko (1988) show that reach (cheliceral length), gape (moveable digit length), cheliceral height, and *L*1*U* moment arm increase with body size over all feeding types (*plots not shown*). The 47 astigmatid species examined as a set thus do not appear to ordinate exactly like the oribatids investigated to date. Phylogeny might be important.

Despite reasonable agreement between this study (Table 2) and velocity ratio values calculated from the figure on p353 of Akimov and Gaichenko (1976) of: 0.392 for *Carpoglyphus lactis*, 0.500 for *Kuzinia laevis*, 0.510 for *Acarus siro*, but not with the 0.833 for *Chortoglyphus arcuatus*, differences in velocity ratio on its own between mites does not seem important in predicting astigmatid habitat.

**Fig. 7:**
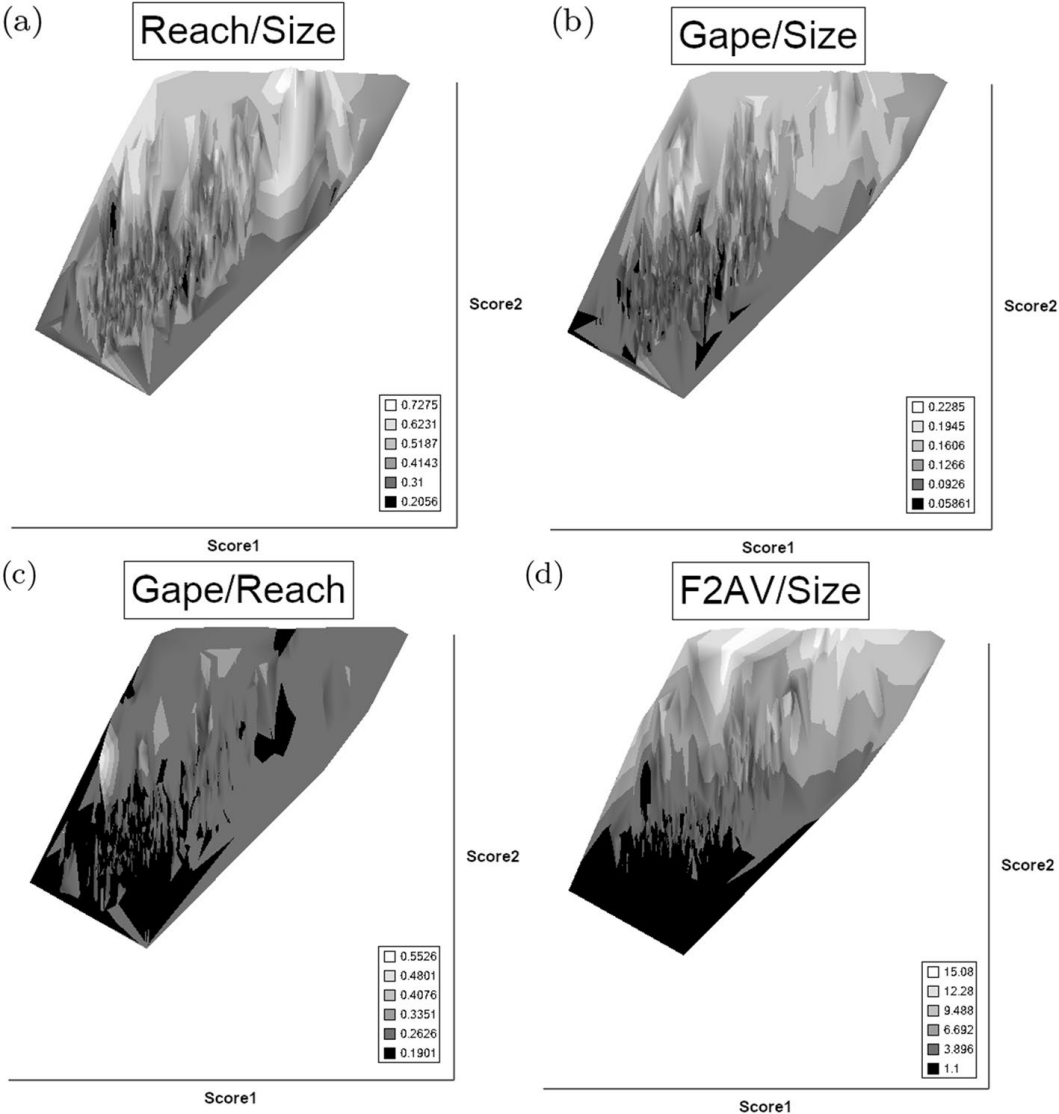
Heat-map of extra derived measures over ordination of reviewed astigmatid species. Legend for heat-map included (dark = low ratio, pale = high ratio). Note all measures agree in compass direction. Note anomalous plateau in relative reach (**a**), relative gape (**b**) and relative crunch force (**d**) to ‘North/NorthWest’, the location of surprisingly small mites for their gnathosomal investment. Such species could ‘pack a punch’ versus hard food morsels for their size. **a** Reach over Size (*CLI*/*IL*). Complicated gradients of disproportionate reach along South-West to North-East trend. **b** Gape over size (*L*2*M*/*IL*). Complicated gradients of disproportionate gape along South-West to North-East trend. Possible evidence of stabbing form? **c** Gape over Reach (*L*2*M*/*CLI*). Complicated gradients of disproportionate gape along gentle roughly South-North trend. Possible evidence of tweezering form? **d** Relative crunch force (*F*2*AV*/*IL*). Convoluted ‘South-West to North-East’ gradient of disproportionate power. Mites in the black zone are weaker in squashing foodstuff than their size would expect (microbial ‘plankton feeders’?)

Furthermore, little extra information is gained by looking at other relative measures as typically used in morphometric analyses (Fig. 7) except that there is a plateau of high values for size-adjusted measures to the ‘North’ (compare this pattern to that for the aspect ratio in Fig. 6b). To the ‘West’ of here sit relatively small mites for their reach and gape who can ‘pack a punch’ with their chelae (i.e., have high *F*2*AV*/*IL* values due in part to high velocity ratios; Fig. 6a). Factoring out general astigmatid scale by division with *IL* showed no clear food or habitat correlate with the general ’South-North’ or ‘SouthEast-NorthWest’ gradients (*results not shown*).

**Fig. 8:**
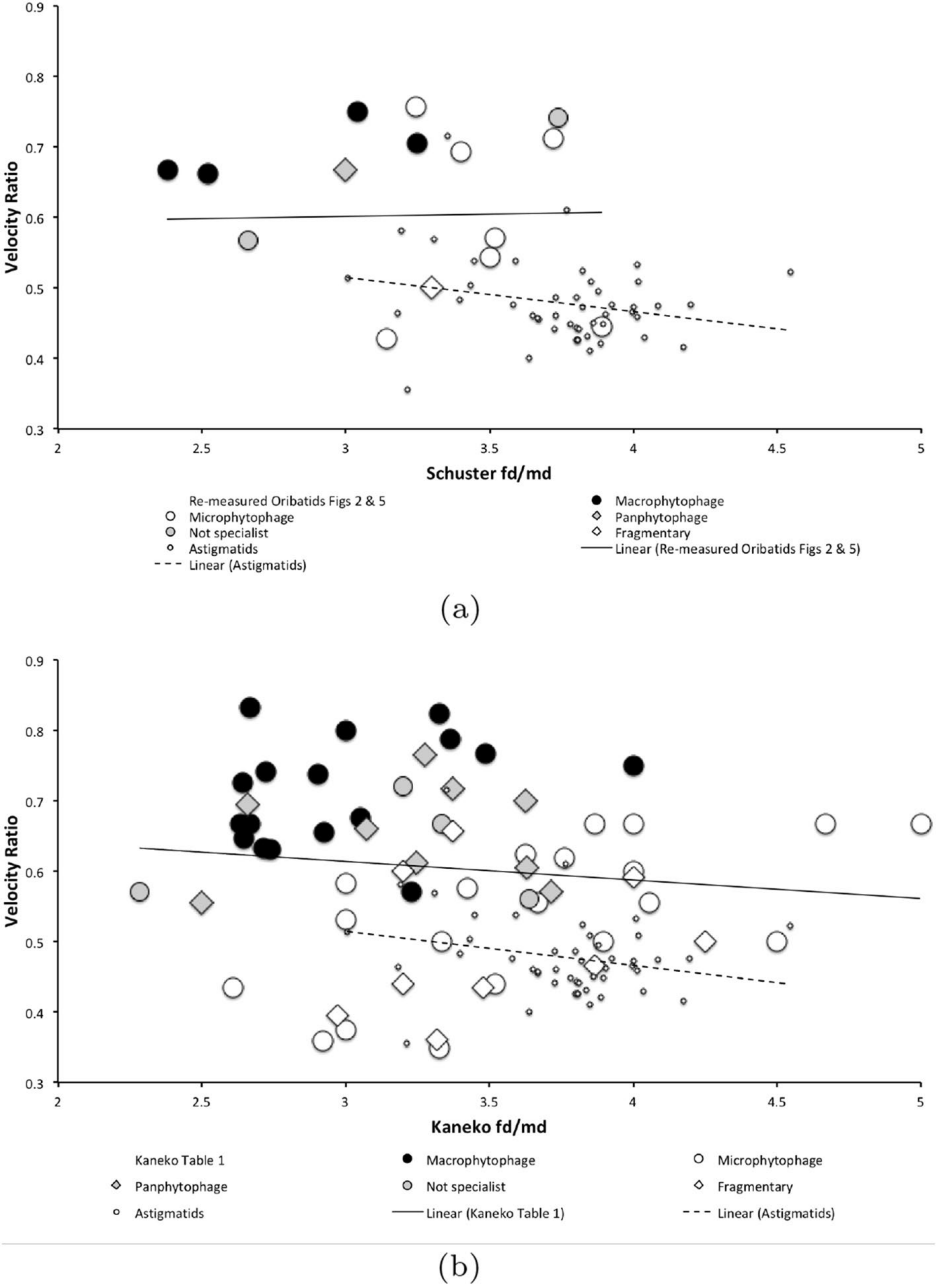
Plot of reviewed astigmatids in space of oribatids used by historical authors. Symbols of different size simply for clarity between astigmatids and oribatids. **a** Remeasured from: Schuster (1956) and, Kaneko (1988). Velocity ratio figures estimated from their Figs. 2 & 5 respectively, feeding classification and $\frac{fd}{md}$ from their Table 3 and Fig. 2. Oribatids re-measured: *Amerus troisii*, *Archoplophora viliosa*, *Belba verticillipes*, *Ceratoppia sexpilosa*, *Eohypochthonius magnus*, *Epilohmannoides esulcatus*, *Gymnodamaeus bicosiatus*, *Hermaniella granulata*, *Heterobelba stellifera*, *Laicarus acutidens*, *Nothrus silvestris*, *Oppiella nova*, *Protoribates lophotricus*, *Rhysotritia ardua*, *Steganacarus* cf. *clavigera*, *Xenillus tegeocranus*. Note that *Gustavia microcephala*—micro-phytophagous, appears to have no moveable digit, *Pelops* cf. *hirtus*—Not specialist, *Eupelops* sp.—micro-phytophagous, is not included due to ambiguity in judging cheliceral length from published Figures. Note markedly different regression lines for each sub-order. **b** Original data from Kaneko (1988) Table 1. All species included—now can include *Eupelops = Pelops* sp. as given in the original Table. Note now high degree of overlap between astigmatids and oribatids plus similar slope regression lines (three species off plot to the right: *Operculoppia restata* at $(\frac{fd}{md}, VR) = (6.00, 0.600)$, *Eupelops* sp. ‘B’ at (8.33, 0.304), and, *Eupelops* sp. ‘R’ at (10.0, 0.500)

Due to a difference in how body size was measured in earlier works, it was not possible to compare the Reach/Size values with those of oribatids (e.g., the Table in Kaneko 1988), but for sure, values for astigmatid Gape/Reach are not similar to those of detritophagous oribatids (Schuster 1956 and Fig. 8 (a); note his Table 3 of $\frac{fd}{md}$ is broadly equivalent to $\frac{Gape}{Reach}$ in Table 2 herein). His $\frac{fd}{md}$ range was 2.50–3.82 for oribatids equivalent to 0.262–0.400 in *L*2*M*/*CLI*. Amongst the astigmatids: the lowest $\frac{fd}{md}$ measure is 3.00 for *Dermatophagoides farinae* (D4) with a *L*2*M*/$CLI \equiv 0.333$, the highest $\frac{fd}{md}$ measure is 4.55 for *Lardoglyphus konoi* (L1) with a *L*2*M*/$CLI \equiv 0.220$. Schuster (1956) appears to have classified his mites: with a $\frac{fd}{md}$ value less than about 3.08 (*L*2*M*/*CLI* equivalent to 0.325) as “macrophytophagous”, those with a $\frac{fd}{md}$ value greater than about 3.29 (*L*2*M*/*CLI* equivalent of 0.304) as “microphytophagous”, and those in between as not particularly specialised. Given this, 43 out of the 47 species of astigmatids would be classed as “$\equiv$ macrophytophagous oribatids”, only *Dermatophagoides farinae* (D4) being as “$\equiv$ microphytophagous oribatids”, and *Carpoglyphus lactis* (Ca4), *Chortoglyphus arcuatus* (CH1) and *Kuzinia laevis* (KL) as not particularly specialised. Nine astigmatid species (*Sancassania berlesei* (C3), *Lardoglyphus konoi* (L1), *Lardoglyphus zacheri* (L3), *Suidasia pontifica* (S5), *Tyrophagus nieswanderi* (T6), *Tyrophagus palmarum* [’A’] (T17), *Tyrophagus palmarum* [‘B’] (T32), *Madaglyphus legendrei* (T34), and *Tyrophagus tropicus* (T90)) have $\frac{fd}{md}$ measures greater than Schuster (1956) found. They would appear using this criterion to be “ultra-macrophytophages”? Care in re-using terminologies across mite groupings is needed. This non-overlapping pattern of evidence between mite groupings (together with the distinct regression lines in Fig. 8a) is surprising and suggests that astigmatids might not be just simply paedomorphic oribatids, with some seeming to be ’super oribatids’ in design! Is using Schuster (1956)’s approach actually valid? Matters become clearer if one compares the astigmatids to Kaneko (1988)’s results (Fig. 8b) who gives detailed data for more taxa. Astigmatids now *do* sit nicely in the range of microphytophagous oribatids with a similar regression relationship. Astigmatids and oribatids *are* congruent! The span of species used in comparisons when redeploying subjective criteria is very important. Historical data needs care.

To summarise. Trophic shape does not appear to change markedly with astigmatid size. Mite species simply grade by scale, the scree plot suggesting most variation is explained by the first component (which is broadly astigmatid size in most of its forms). These 47 astigmatids species are mainly just ‘shrinkings/swellings’ of a common body plan. Allowing for this scaling, then small amounts of extra variation are explained for by: the sizes and relative proportions of the cheliceral height and lengths, and the sizes and relative proportions of the chelal height and lengths. Thus beside any differences in general scale, astigmatid chelicerae mainly vary in their height to length proportion (‘aspect ratio’). Visually crunch force (*F*2*AV*) appears to be a cheliceral height dominated measure (chelal height correlates well with final adductive force crunch force; *F*2*AV* $R^{2}=0.8808$). Adductive force appears to be the key design (*D*) measure and one that can be altered semi-independently of body size (Fig. 7d). A type of ‘gnathosomisation’ by structural height changes is certainly indicated (with possibly a degree of secondary relative shrinkage in body size for certain powerful chelal forms i.e., in pyroglyphids?). Unlike say in mesostigmatids, the open nature of the dorsal part of the gnathosoma in free-living astigmatids gives the opportunity for such differential growth at any one general mite size if there is the evolutionary pressure.

### What can be said about biological relations (*B*) from astigmatid mechanical design (${\hat{D}}$)?

**Fig. 9:**
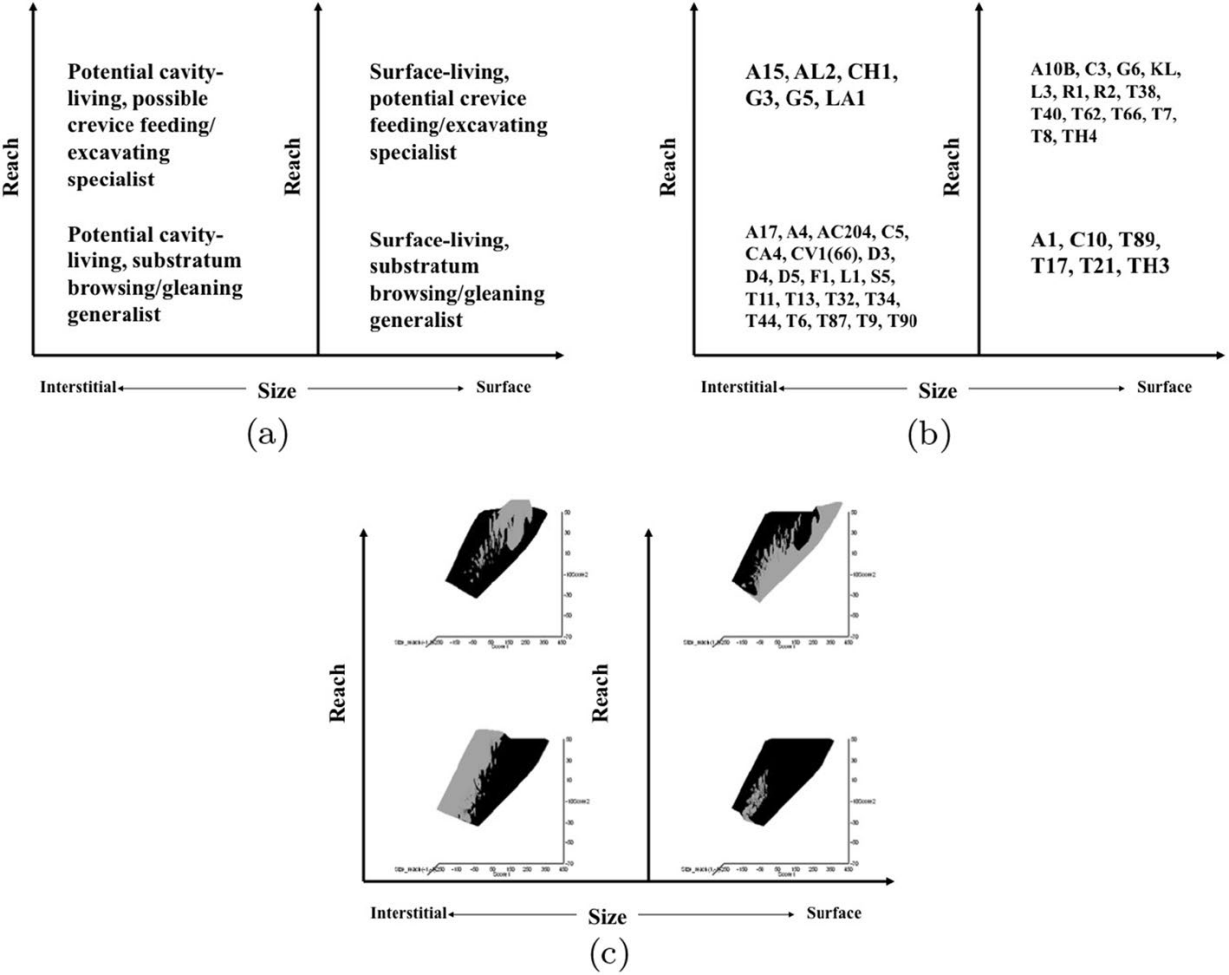
Food access as determined by cheliceral design—four-box summary model (1.) for size (*IL*) and reach (*CLI*) defining habit. Species codes as in Table 1. **a** Biological schema. **b** Species allocated to each quadrant (based on their average value). **c** Heat-map of species’ individuals (in grey) within black design space for each quadrant. Cavity-living may indicate a burrowing habit. Diagonality of sub-figures (bottom left to top right in **c**) is indicating a congruent scale change. Diagonality of sub-figures (top left to bottom right in **c**) is indicating an overall shape change. Small, big reach mites (*Acarus siro* SW sp. (A15), *Aleuroglyphus ovatus* (AL2), *Chortoglyphus arcuatus* (CH1), *Glycycometrus hugheseae* (G3), *Glycyphagus domesticus* (G5), *Neosuidasia* sp. (LA1)) have commonality with the upper groups in Figs. 24c and 25c

**Fig. 10:**
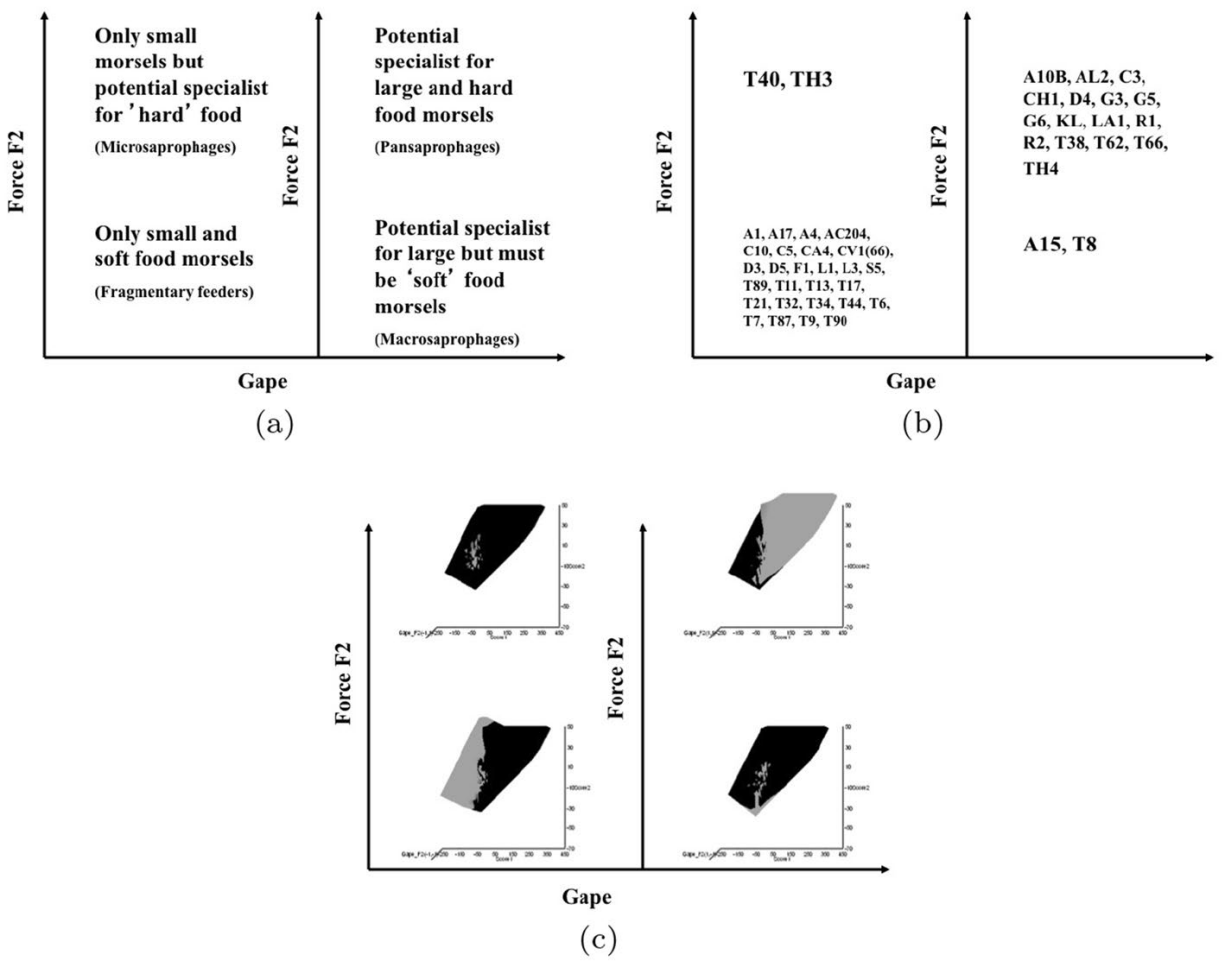
Food handling as determined by chelal design—four-box summary model (2.) for gape (*L*2*M*) and crunch force (*F*2*AV*) defining saprophagous subtypes. Species codes as in Table 1. **a** Biological schema. **b** Species allocated to each quadrant (based on their average value). **c** Heat-map of species’ individuals (in grey) within black design space for each quadrant. Small gape suggests selective feeding on small spores, particles, thin fungal hyphae etc. Large gape indicates the possibility of being an indiscriminate feeder of large and small spores, particles, both fat and thin fungal hyphae etc. Mites with low F2 may be nematode/microbiota eaters. If so, *Acarus siro* SW sp. (A15) and *Tyrophagus perniciosus* [‘A’] (T8) could tackle large worms. Diagonality of sub-figures (bottom left to top right in **c**) is indicating a congruent hardness increase with scale change. Diagonality of sub-figures (top left to bottom right in **c**) is indicating particular specialisms. *Tyrophagus longior* (T40) and *Thyreophagus entomophagus* (TH3) top left $\rightarrow$ ‘tweezers’ for hard/intractable, small, possibly hidden food items. *Acarus siro* SW sp. (A15) and *Tyrophagus perniciosus* [‘A’] (T8) bottom right $\rightarrow$ particularly large particularly soft food items or gnawing/tearing off chunks of very tractable material

**Fig. 11:**
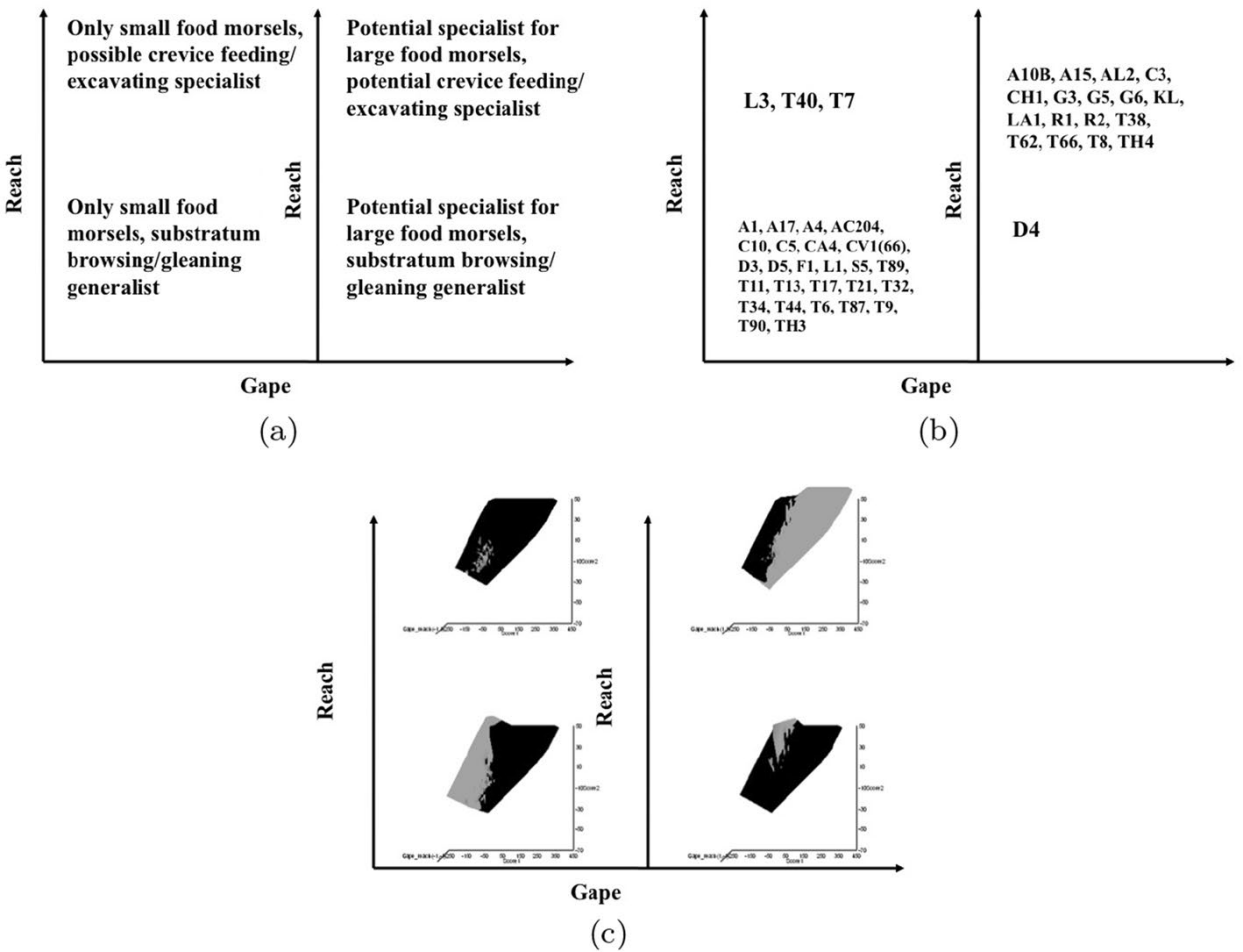
Four-box summary model for gape (*L*2*M*) and reach (*CLI*). Species codes as in Table 1. **a** Biological schema. **b** Species allocated to each quadrant (based on their average value). **c** Heat-map of species’ individuals (in grey) within black design space for each quadrant. *Lardoglyphus zacheri* (L3), *Tyrophagus longior* (T40) and *Tyrophagus vanheuri* (T7) may be seekers of food morsels at a distance (small nematodes, hidden microbiota, sequestered ‘nuggets’ of nutrition etc.?). Note anomalous position of *Dermatophagoides farinae* (D4) as a potential feeder of nearby large fragments (D4 is also in the upper group of Figs. 24c and 25c)

**Fig. 12:**
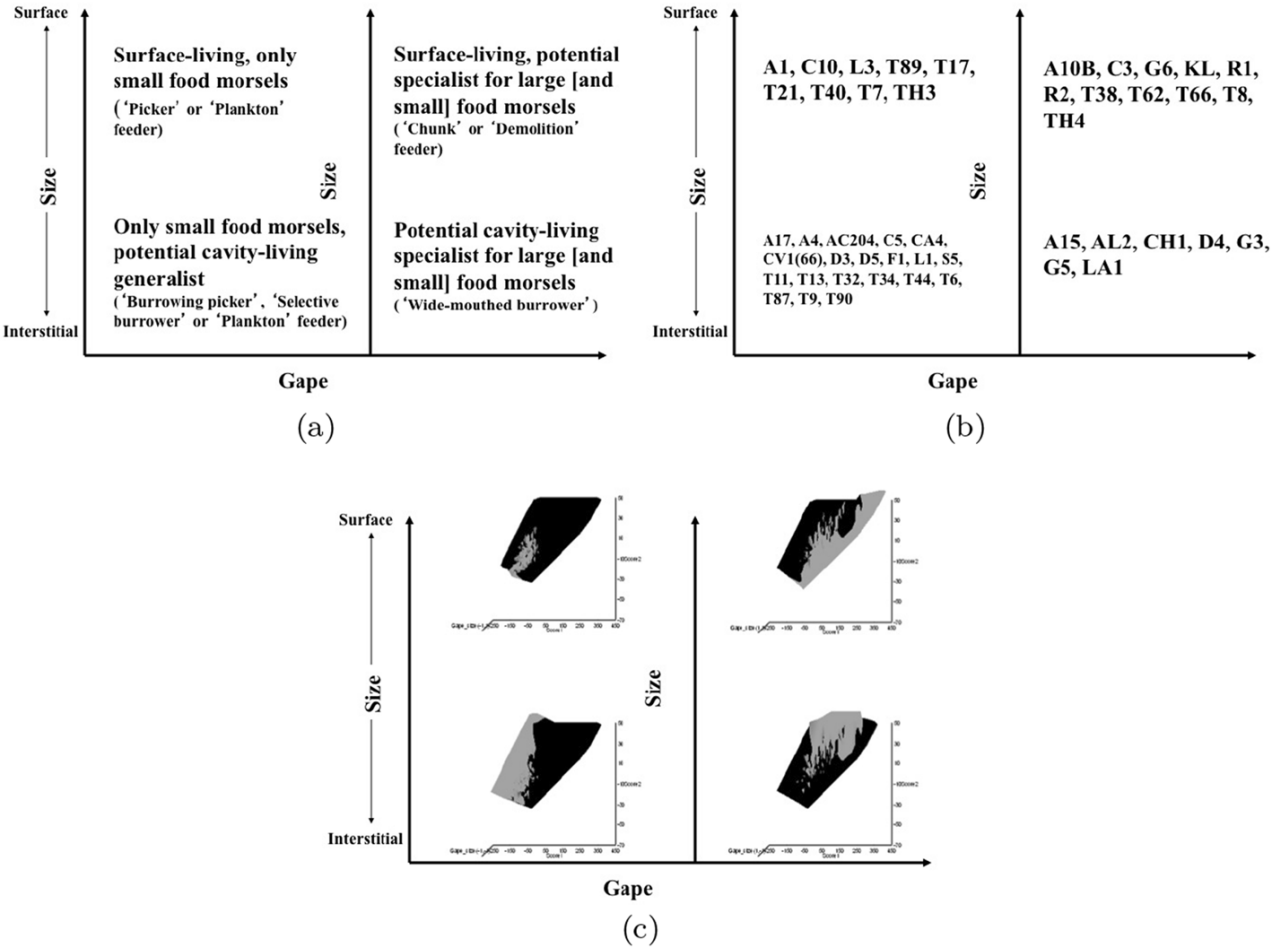
Four-box summary model for gape (*L*2*M*) and size (*IL*). Species codes as in Table 1. **a** Biological schema. **b** Species allocated to each quadrant (based on their average value). **c** Heat-map of species’ individuals (in grey) within black design space for each quadrant. The ‘wide-mouthed burrower’ species (*Acarus siro* SW sp. (A15), *Aleuroglyphus ovatus* (AL2), *Chortoglyphus arcuatus* (CH1), *Dermatophagoides farinae* (D4), *Glycycometrus hugheseae* (G3), *Glycyphagus domesticus* (G5), *Neosuidasia* sp. (LA1)) have a high commonality with the upper groups in Figs. 24c and 25c

**Fig. 13:**
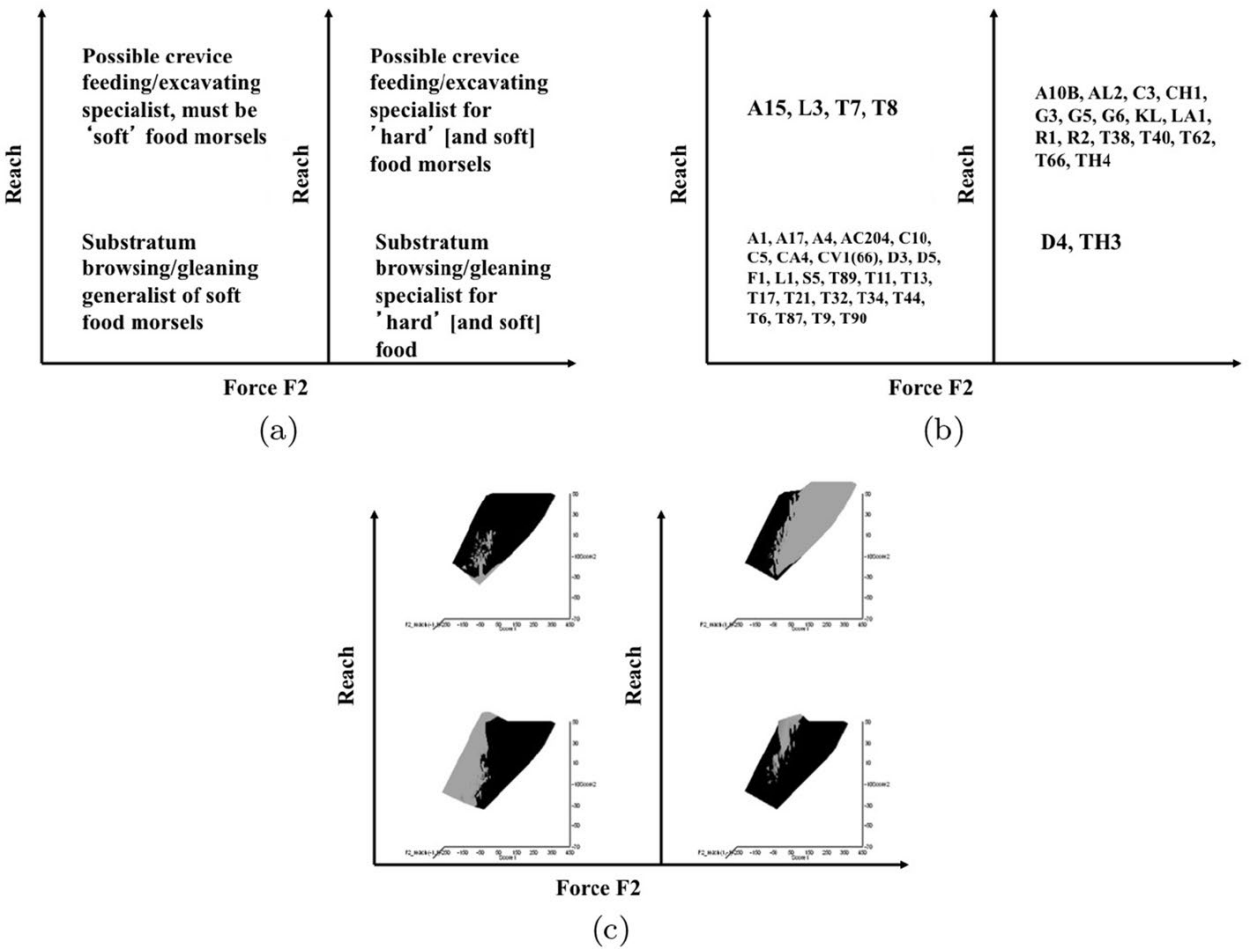
Four-box summary model for crunch force (*F*2*AV*) and reach (*CLI*). Species codes as in Table 1. **a** Biological schema. **b** Species allocated to each quadrant (based on their average value). **c** Heat-map of species’ individuals (in grey) within black design space for each quadrant. Note anomalous position of *Dermatophagoides farinae* (D4) which is also in the upper group of Figs. 24c and 25c)

**Fig. 14:**
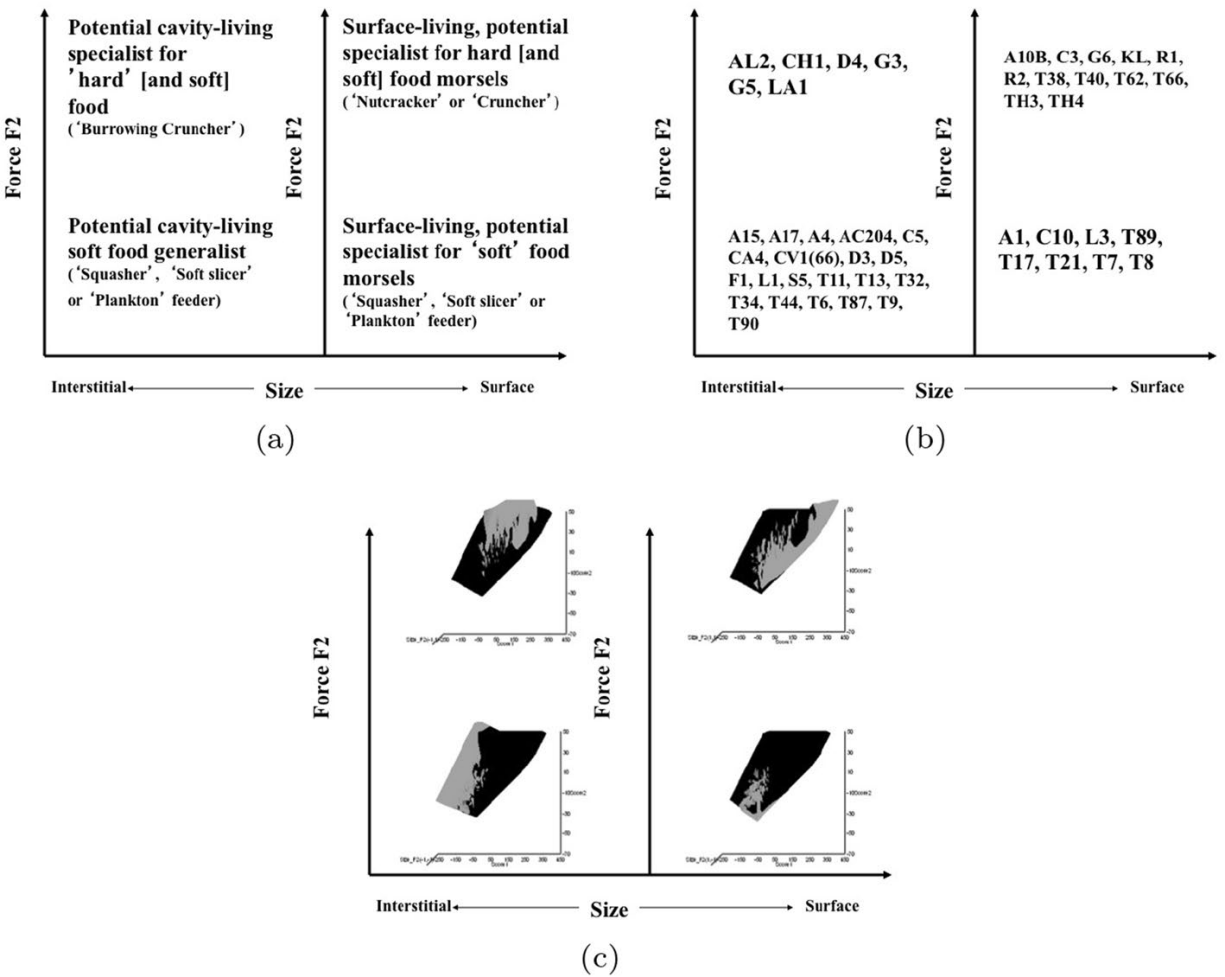
Four-box summary model for size (*IL*) and crunch force (*F*2*AV*). Species codes as in Table 1. **a** Biological schema. **b** Species allocated to each quadrant (based on their average value). **c** Heat-map of species’ individuals (in grey) within black design space for each quadrant. The ‘burrowing cruncher’ mites species (*Aleuroglyphus ovatus* (AL2), *Chortoglyphus arcuatus* (CH1), *Dermatophagoides farinae* (D4), *Glycycometrus hugheseae* (G3), *Glycyphagus domesticus* (G5), *Neosuidasia* sp. (LA1)) have a high commonality with the upper groups in Figs. 24c and 25c

The two key summary models posed in the “Expected results” section above are given in Figs. 9 and 10. The size and shape change described above is illustrated in four-box model (1.) in Fig. 9. A longer reach may facilitate finding deeper buried nutritional sources (‘treasure’) or being nematophagous (cf. a longer reach allows a mite to approach its prey with less chance of alerting it due to body proximity). The biological relevance of four-box model (2.) is explained in Fig. 10. Only a modest number of saprophagous species show clear trophic specialisation over and above the typical average mite design (Table 2). In these specialists, various combinations (Figs. 11-14) of adaptations for food access (Fig. 9) and food handling (Fig. 10) arise depending upon the relative interplay of length versus height measures. *Dermatophagoides farinae* (D4) appears particularly distinct in its design. There is also a fairly clear group of potentially interstitial substrate-excavating large reach wide-mouthed high crunch force species different from the basal surface-living browsing/gleaning/squishing ‘plankton/microbe/picking’ astigmatid form. The heuristic approach *is* insightful of potential adaptive syndromes in astigmatids.

**Fig. 15:**
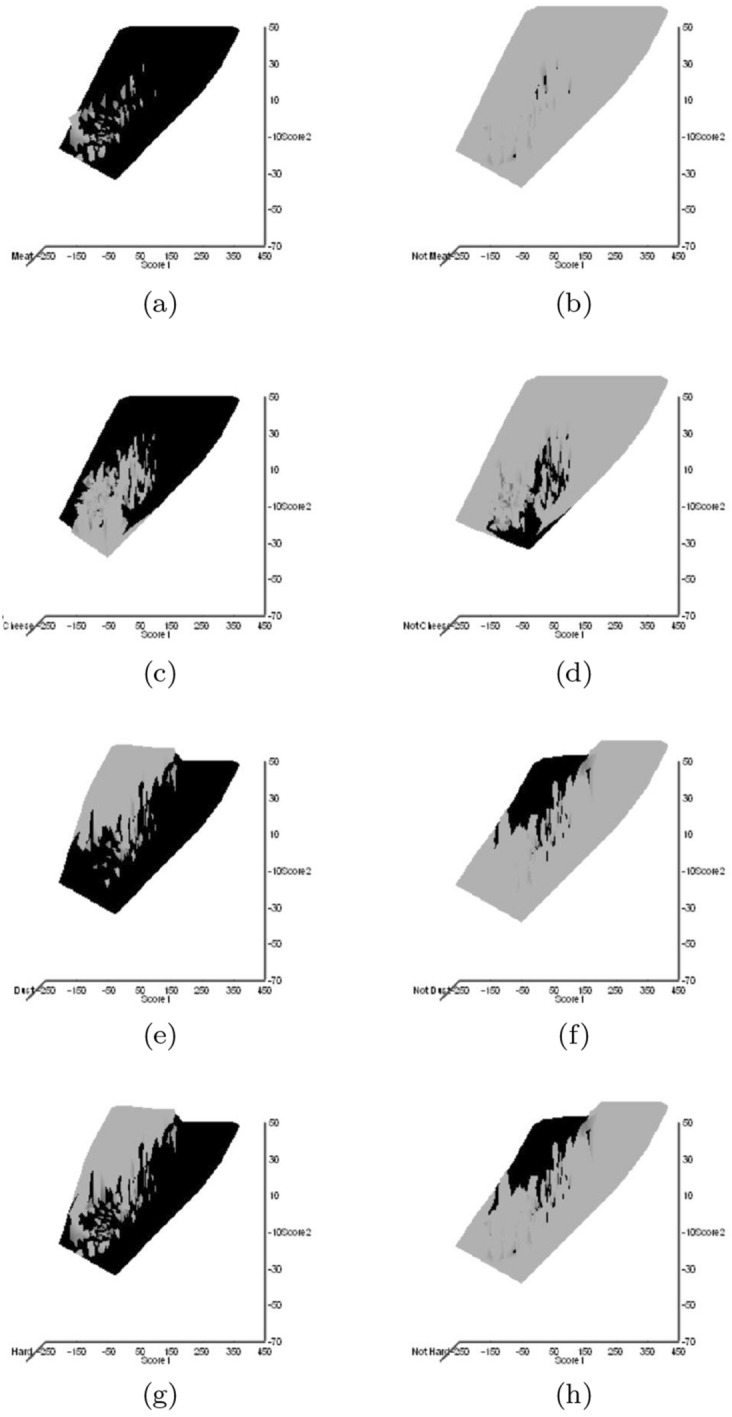
Duplicate mirror-image heat-map over ordination of reviewed astigmatid species for statistically significant associations (see Table 5 for food type in pairs from Hughes 1976). Each species’ individuals in grey within black design space. **a** Meat, **b** Not meat, **c** Cheese, **d** Not cheese, **e** Dust, **f** Not dust, **g** Hard, **h** Not hard

**Fig. 16:**
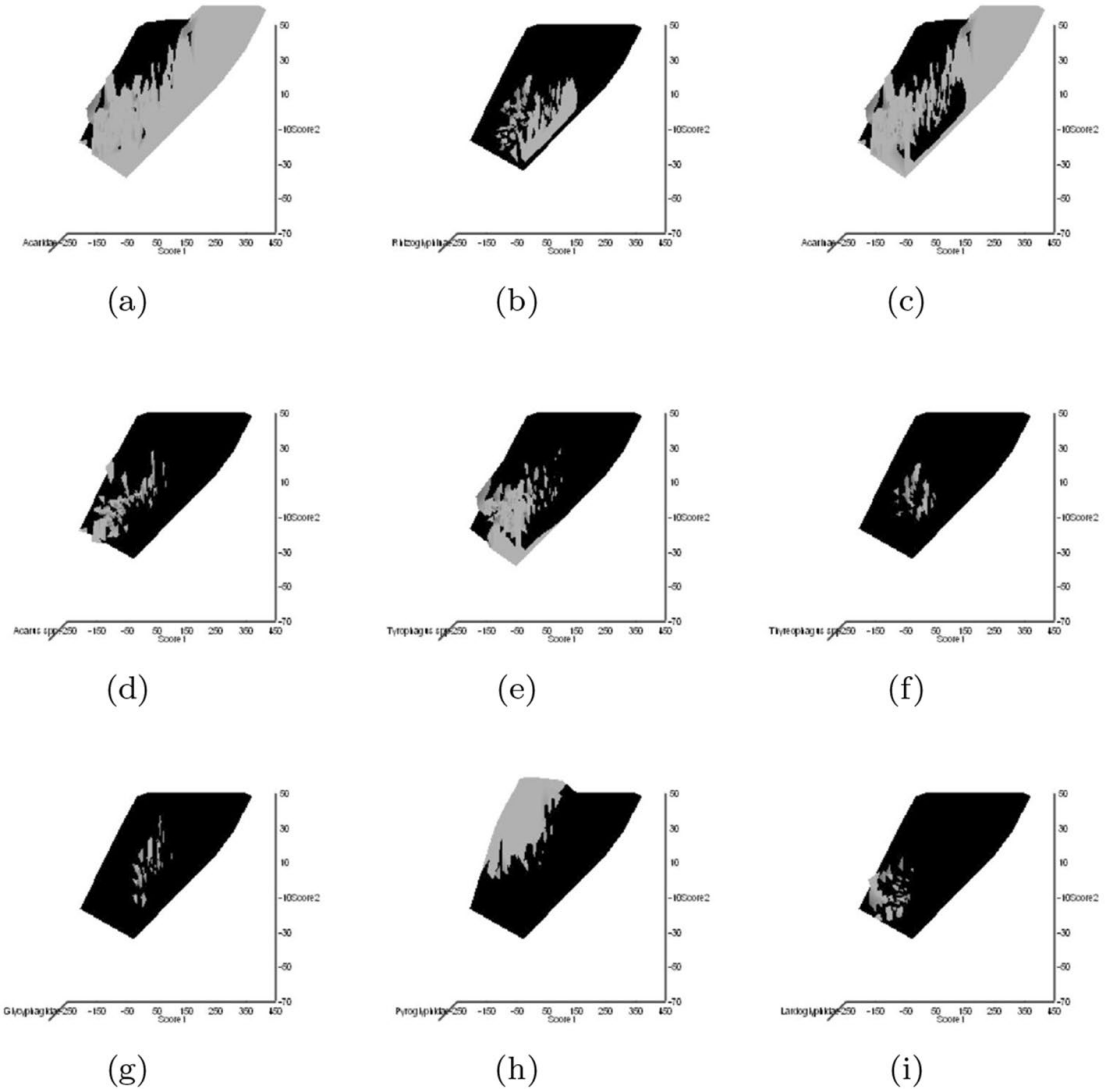
Heat-map over ordination of reviewed astigmatid species for families, subfamilies and genera where this study covers more than one species. See Table 5 for statistical tests. Each species’ individuals in grey within black design space. **a** All Acaridae, **b** Rhizoglyphinae, **c** Acarinae, **d** *Acarus* spp., **e** *Tyrophagus* spp., **f** *Thyreophagus* spp., **g** Glycyphagidae. **h** Pyroglyphidae. **i** Lardoglyphidae which represent fairly well the ‘typical Acaridae’ trophic design

**Fig. 17:**
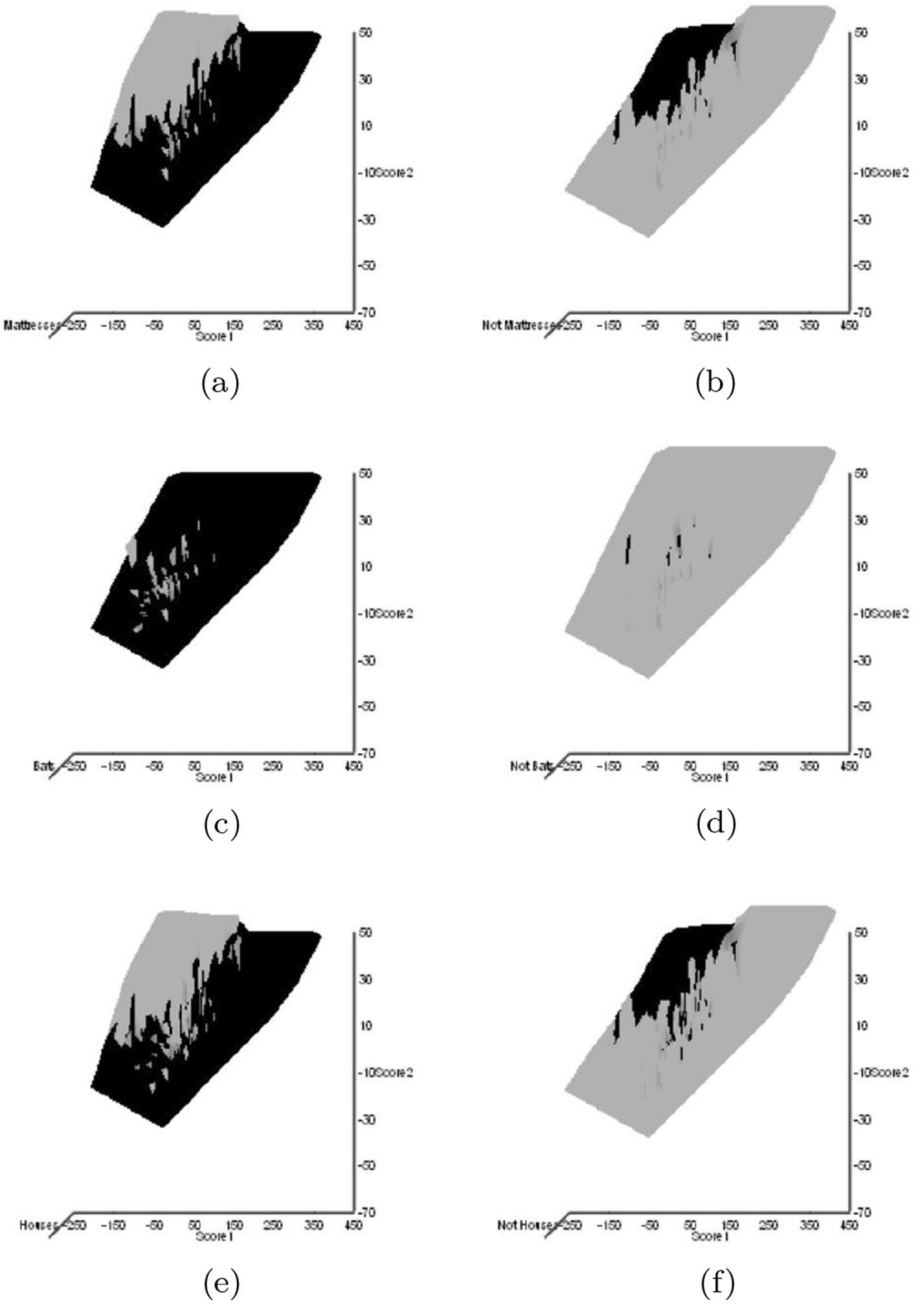
Duplicate mirror-image heat-map (for clarity of inspection) over ordination of reviewed astigmatid species for statistically significant habitats in pairs from Hughes (1976). See Table 5 for statistical tests. Each species’ individuals in grey within black design space. **a** Mattresses, **b** not mattresses, **c** bats, **d** not bats, **e** houses, **f** not houses

**Fig. 18:**
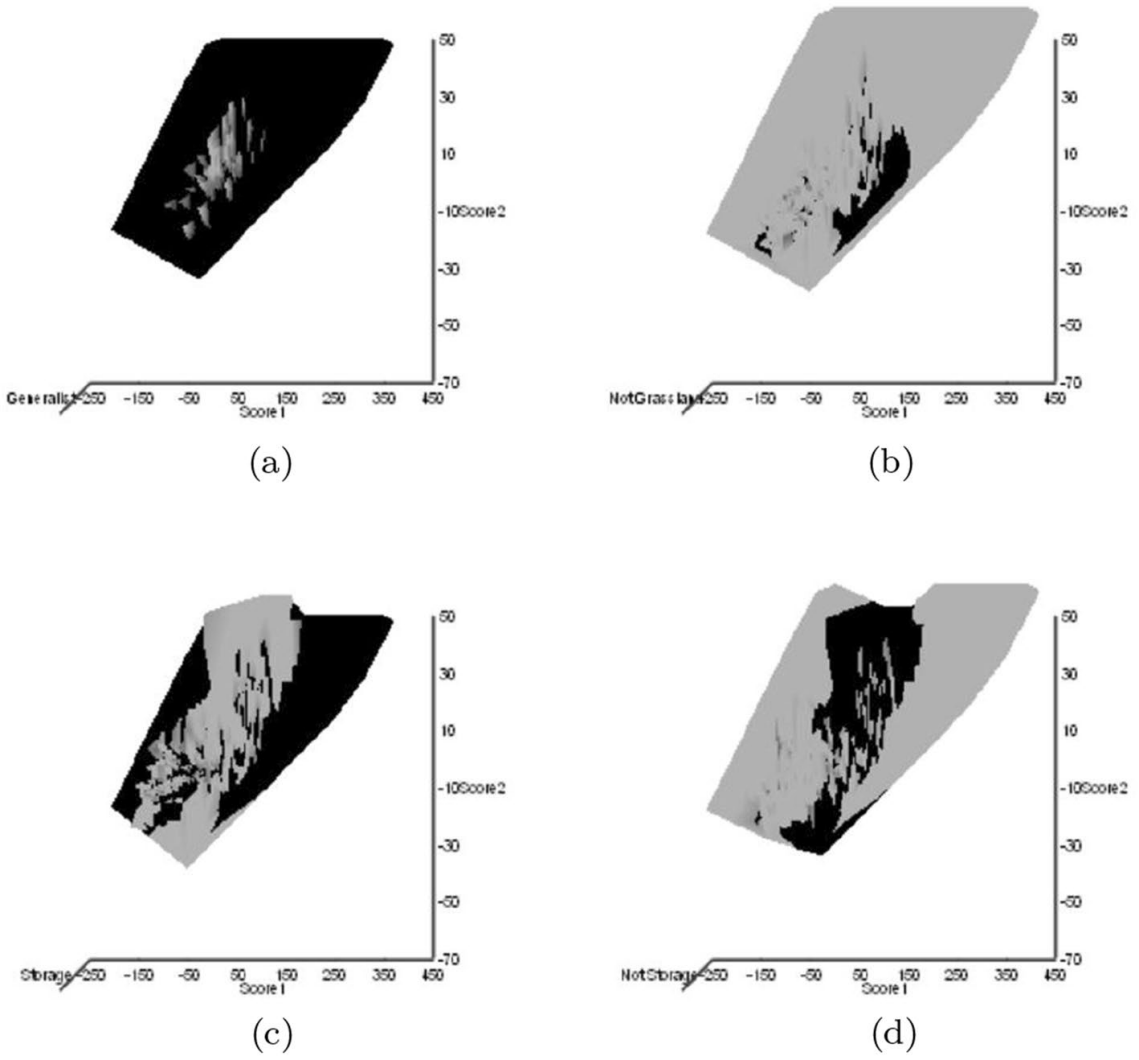
Duplicate mirror-image heat-map (for clarity of inspection) over ordination of reviewed astigmatid species for habitats in pairs from Hughes (1976). See Table 5 for statistical tests. Each species’ individuals in grey within black design space. **a** Grassland, **b** not grassland, **c** storage, **d** not storage

**Fig. 19:**
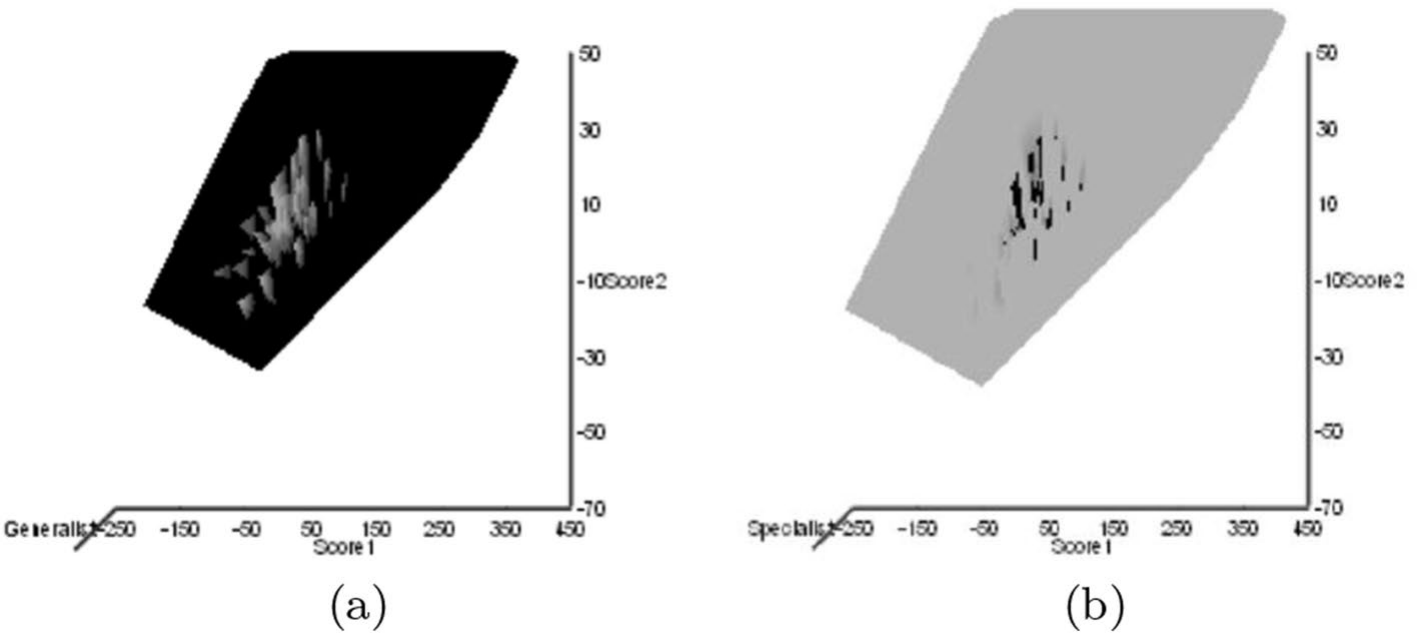
Heat-map over ordination of reviewed astigmatid species for life strategies: Each species’ individuals plotted within design space. See Table 5 for statistical tests. **a** Specialist (in black), **b** Generalist (in black). Note location of generalists well approximates that of the central reference typical mite design group (located at origin)

**Fig. 20:**
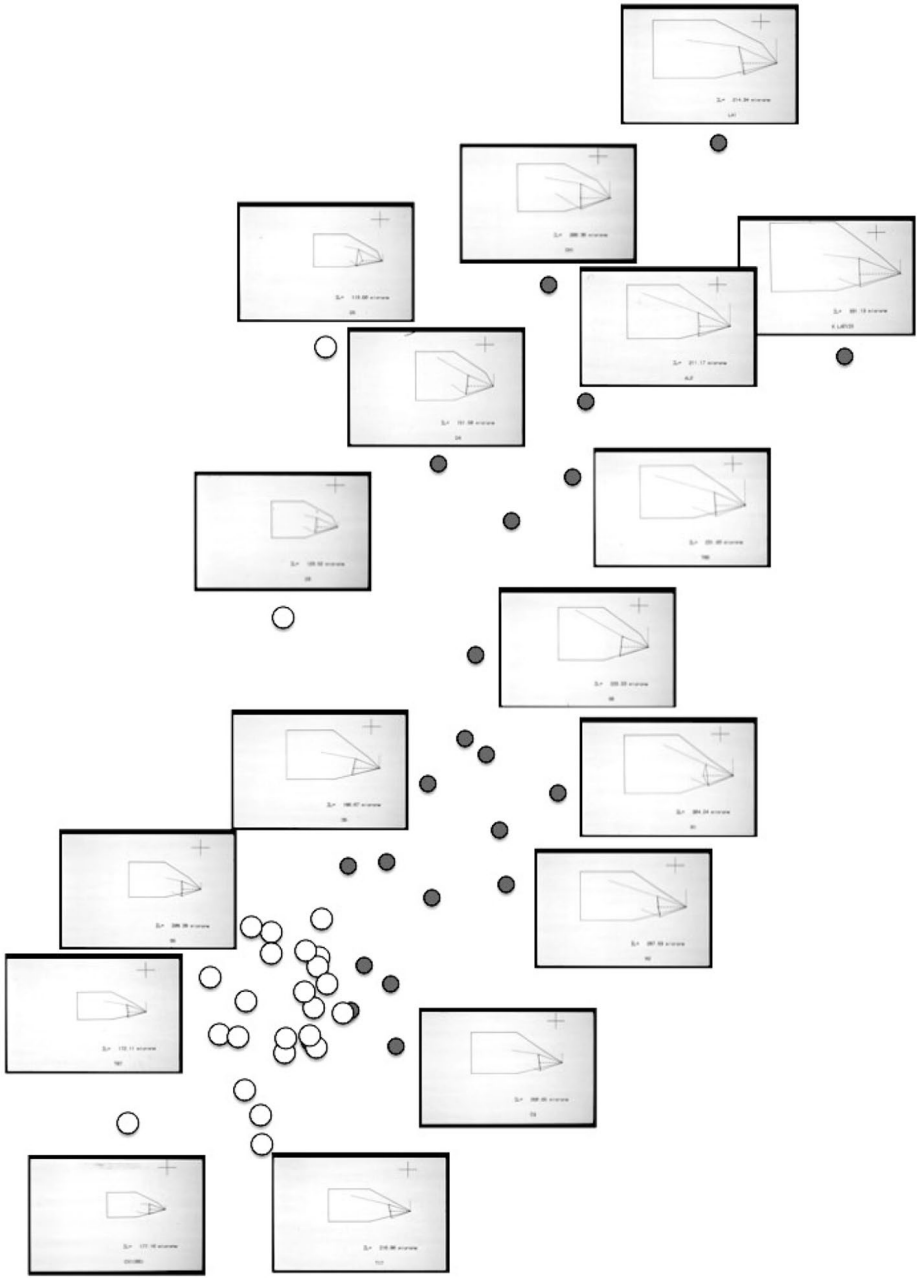
Panels of key astigmatid species cheliceral and chelal designs in block diagrammatic form overlain upon the average trophic ordination scores (Score1 and Score2 from Fig. 5d) at a standard size and format for each species reviewed. Species codes as in Table 1. Species arrangement matches nomogram in Fig. 21. Grey circles = ‘macrosaprophagous’ (demolition feeding) astigmatids. Open circles = ‘microsaprophagous’ (fragmentary feeding) astigmatids. Boundary goes through ‘Typical’ astigmatid. Block diagram shows synthetic depiction of moveable digit, adductive tendon, cheliceral length and cheliceral height all to a common scale. The cross is a standard registration point for the synthetic drawings. The heightening/shortening and scale changing (see Fig. 5c) is up the page. Note those to the right have larger chelicerae, those towards the top the more robust chelae. The smallest, daintiest chelicerae are at the lower left (Fig. 22a)

**Fig. 21:**
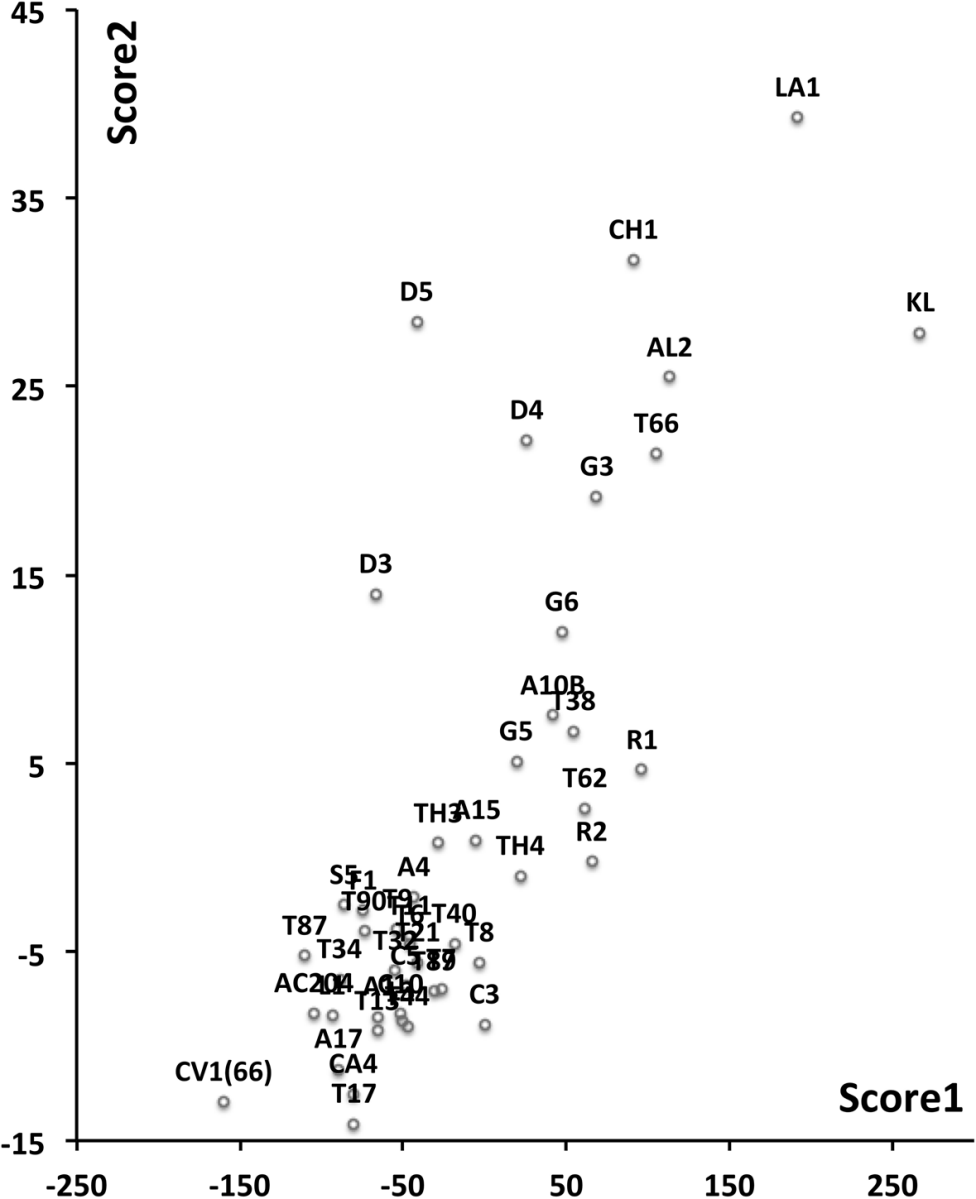
Nomogram ordination of species ($j=1\ldots 46$) in size (*IL*), *L*1*U*, gape (*L*2*M*), *CHI*, reach (*CLI*) and crunch force (*F*2*AV*) in information space. Species codes as in Table 1. Equations are: $Score1= \frac{1}{\sqrt{5.48}}*[\ (0.525*lbf_{j,IL})+(0.889*lbf_{j,L1U})+(0.927*lbf_{j,L2M})+(0.954*lbf_{j,CHI})+(0.906*lbf_{j,CLI})+(0.925*lbf_{j,F2AV})\ ]$ , $Score2= \frac{1}{\sqrt{1.81}}*[\ (-0.796*lbf_{j,IL})+(0.333*lbf_{j,L1U})+(0.010*lbf_{j,L2M})+(0.104*lbf_{j,CHI})+(-0.200*lbf_{j,CLI})+(0.216*lbf_{j,F2AV})\ ]$. Note $lbf_{j,xx}$ is the quadratic discriminant function for individuals (*i*) of a species (*j*) compared to the typical central reference species for measurement *xx* (see Eq. (1) in text). Typical central reference species (measured in $\mu$m) has Mean and SD as in Table 2

**Fig. 22:**
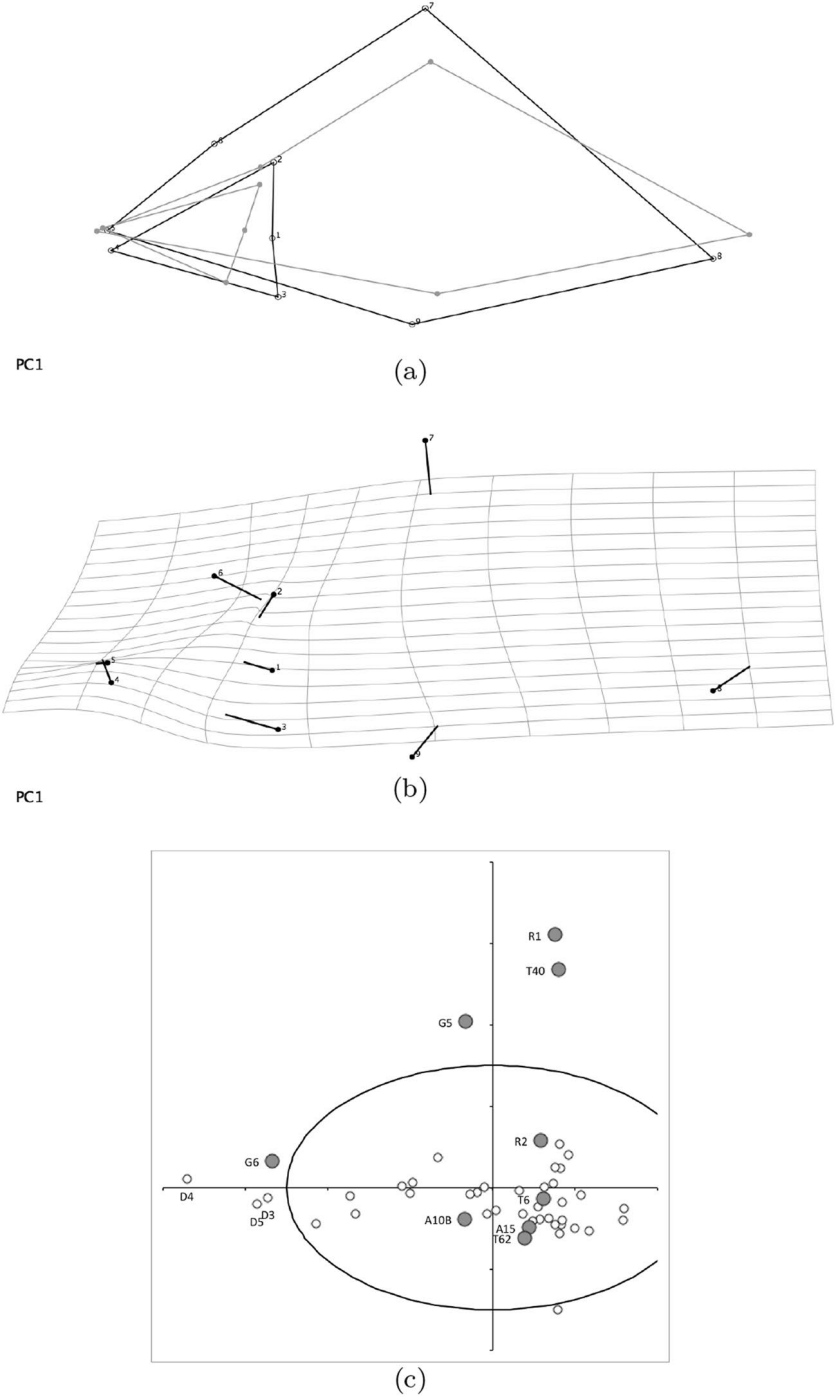
Transformation for each landmark in reviewed astigmatids as in wireframe from Fig. 3c (omitting *CHI* and *CLI* for clarity) based upon first principal component of Procrustes shape analysis (PC1 represents 52% variation between species). **a** Extremes of cheliceral Procrustes shape, effectively equivalent to a transit from robust mites like those top-right in Fig. 20 (dark circles, here dark lines), to daintiest mites like those bottom-left in Fig. 20 (pale circles, here pale lines). Note that even after allowing for size change (any ‘swelling/shrinking’) a fundamental overall aspect ratio change occurs. **b** Vectors for each landmark on thin-plate spline grid (Bookstein 2018). Note how also the shape of the moveable digit alters, together with the positioning of the condyle and fixed digit lyrifissure. (c) Plot of reviewed astigmatid species scores on first two principal components (PC1 is *x*-axis, PC2 representing a further 21% of sample variation is *y*-axis) from Procrustes analysis. PC2 is a relative shortening plus heightening (and vice versa) of just the basal part of cheliceral segment. Species codes as in Table 1. Pest species coloured grey in larger circles and labeled. Ellipse is 90% boundary showing pyroglyphids and glycyphagids (both labelled) have different fundamental shape to most acarids

**Fig. 23:**
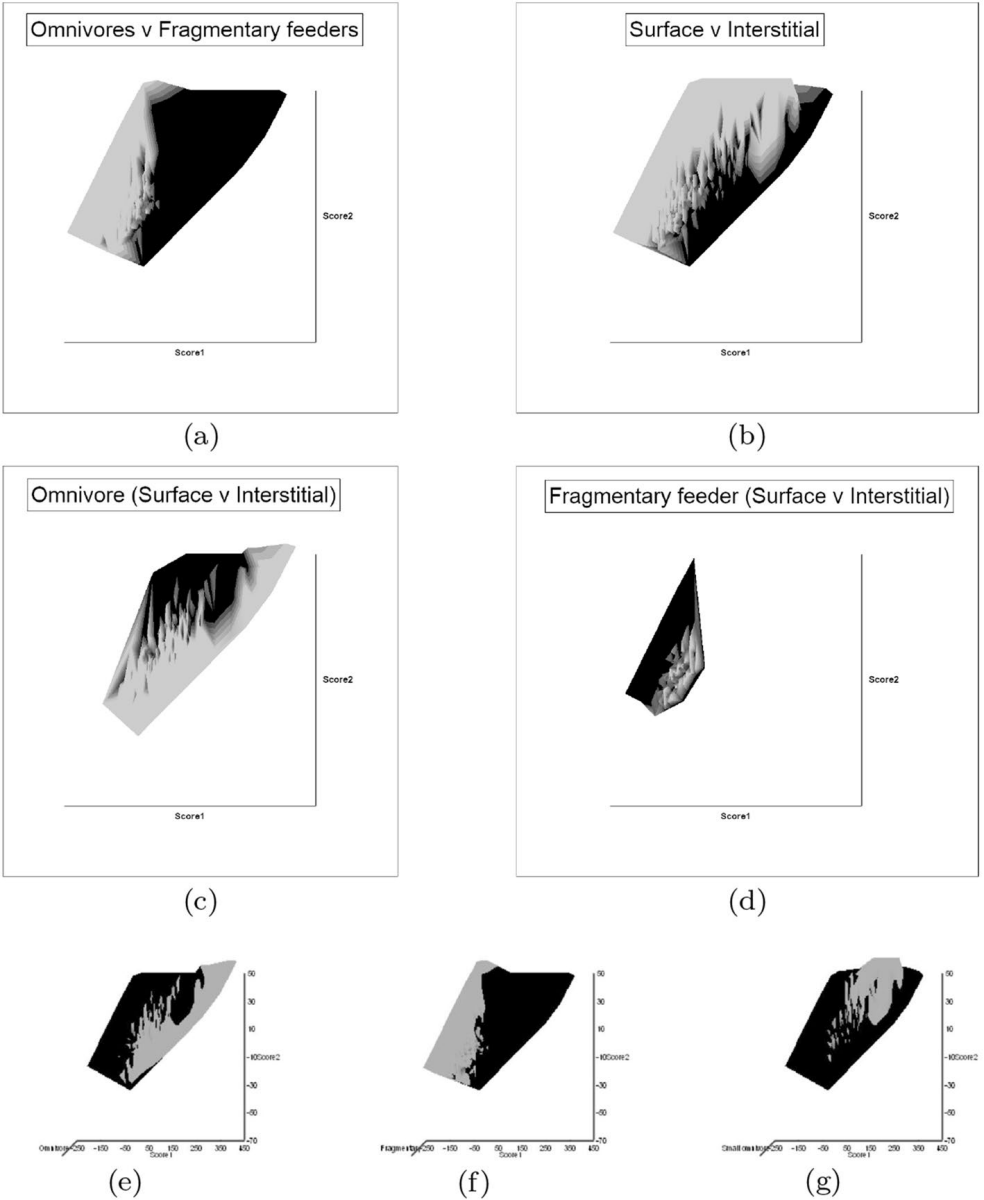
Summary of results for reviewed astigmatids. Species codes as in Table 1. **a**–**d** Heat-maps to match statistically important hypothesis tests in Table 8. **e**–**g** Heat-map of species’ individuals in grey within black design space (darker = lower, paler = higher, ‘North’ vertical on page) as grouped from four-box heuristic modelling conclusions; see Table 8. **e** Omnivores (Type 1.): A10b, C3, KL, R1, R2, TH4, T66, T62, T38 and G6. (f) Fragmentary feeders (Type 2.): AC204, A17, A4, C5, L1, T34, S5, T6, T32, T13, T9, T87, T44, T11, T90, Ca4, D5, D3, CV1(66) and F1. **g** Small omnivores: AL2, LA1, CH1, G3 and G5

**Fig. 24:**
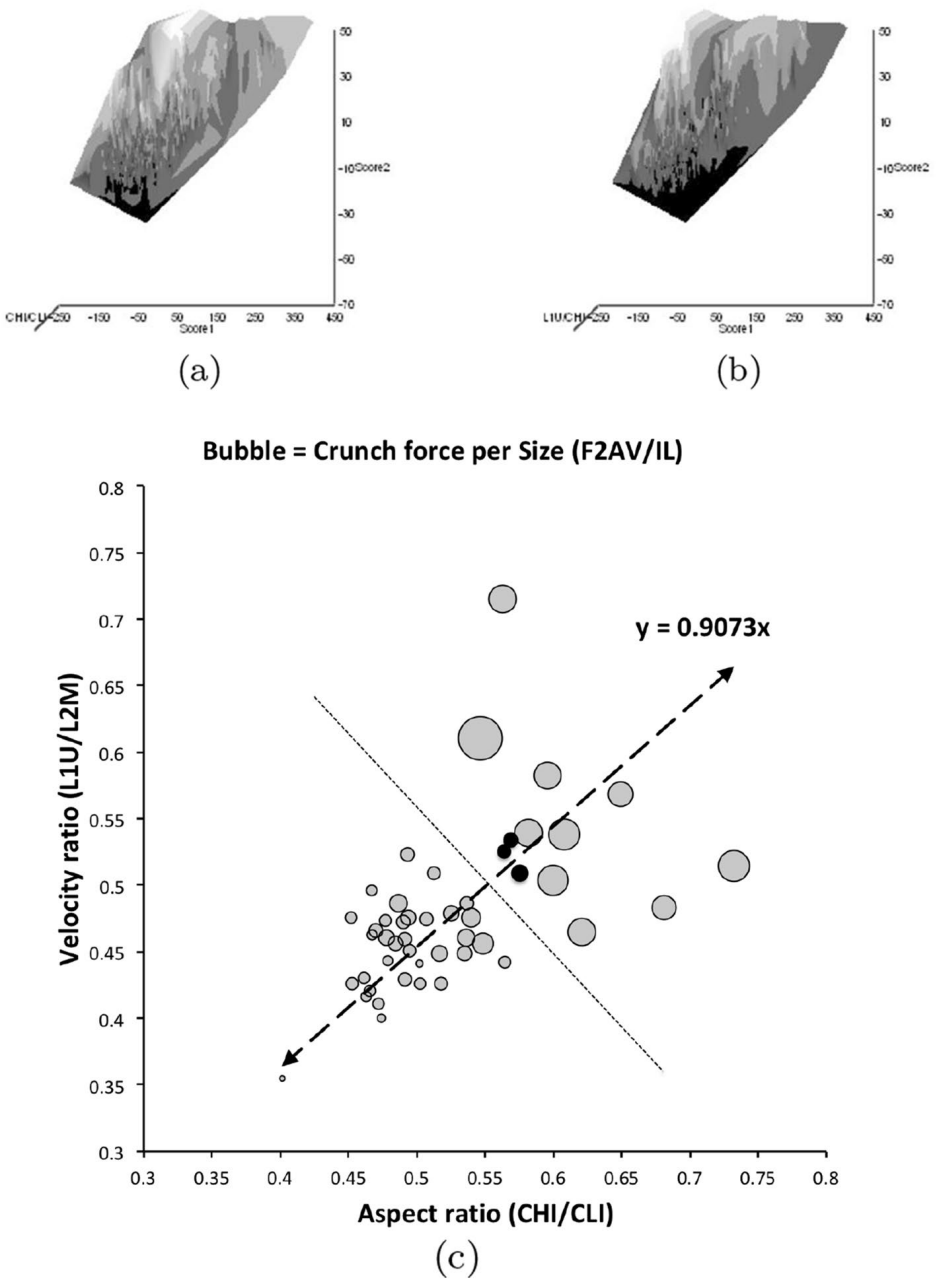
Astigmatid summary of relative measures. Species codes as in Table 1. **a** Cheliceral aspect ratio (*CHI*/*CLI*). Complicated gradients along gentle roughly South-North trend. Mites in the black zone have disproportionately slender chelicerae, those in the white zone are stubby in shape (see Fig. 20). **b** Chelal relative moment arm measure (*L*1*U*/*CHI*). Complicated gradients along gentle roughly South-North trend. **c** Summary plot showing how astigmatids as pale grey circles partition into two groups along axis of almost linear growth (black arrowed regression line). Dotted boundary line is orthogonal to astigmatid regression for clarity. The Upper group (above the boundary) is those species with a powerful ’punch’ adapted for usually hard foods. Most of these species (AL2, CH1, D3, D4, D5, G3, G6, KL, LA1, T66) are included in the set with CL/*IL* values $>50\%$ (i.e., AL2, CH1, D3, D4, D5, G3, G5, LA1, T11, T66), many feature as specialists in Fig. 9, and invariably also have a high Score2 on the ordination (see Fig. 21). Solid black circles are *Suidasia pontifica* (S5), *Thyreophagus entomophagus* (TH3), *Thyreophagus* sp. (TH4) indicating distinctive sub-design. The lower group (below the boundary) is the remaining ‘base-design’ species (with the exception of the low outlier on both axes of *Carpoglyphus lactis* (Ca4) next to the arrowhead on the regression line). The agricultural pest species of *Acarus siro* (A10B) and *Rhizoglyphus echinopus* (R1, R2) sit near the boundary. Crunch force is morphologically estimated as before

**Fig. 25:**
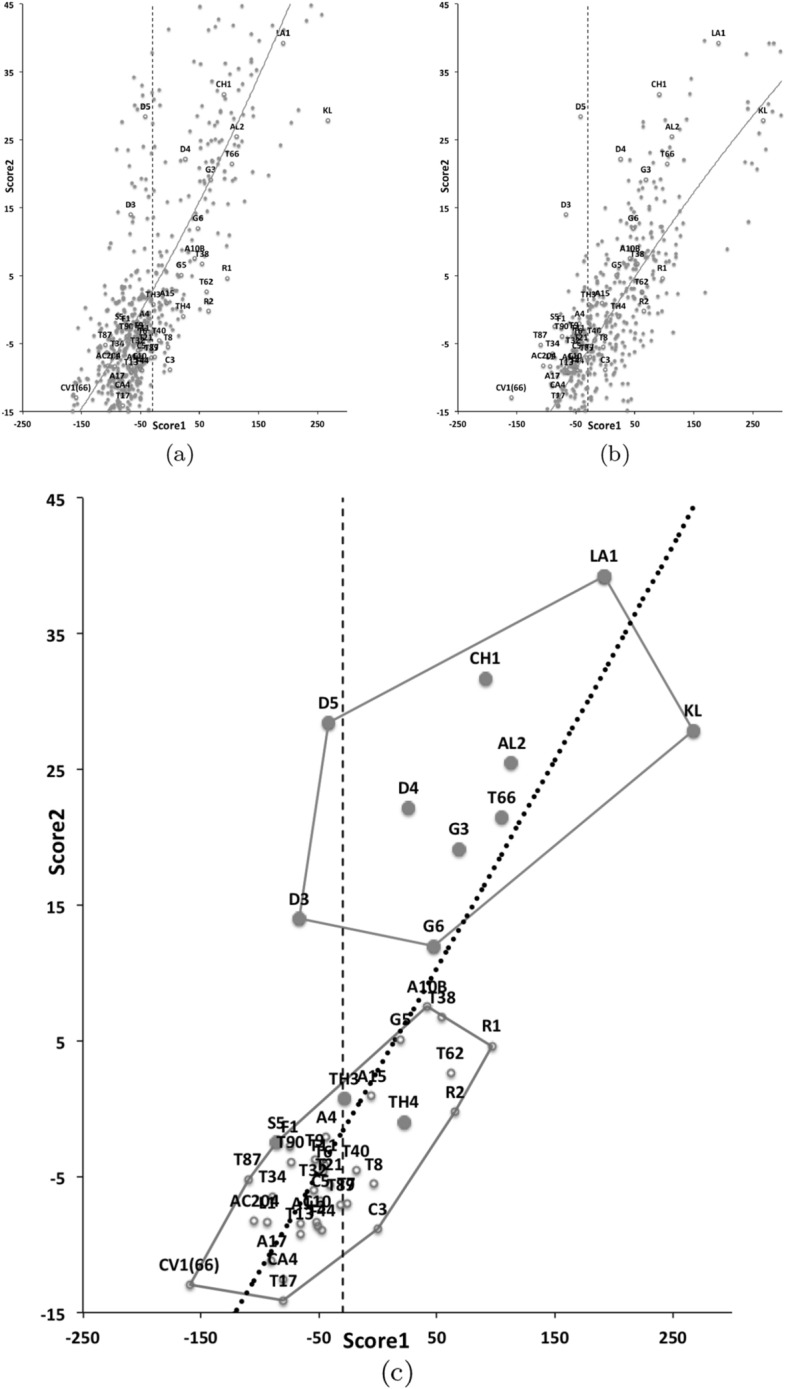
Summary of results for astigmatids reviewed. Species codes as in Table 1. **a** Individual mites overlain upon nomogram in Fig. 21. Interstitial habit plus quadratic fitted summary line. **b** Individual mites overlain upon nomogram in Fig. 21. Surface habit plus quadratic fitted summary line. **c** Major groups of astigmatid feeding design. Species arrangement matches nomogram in Fig. 21. Solid circles species as in Fig. 24c upper group. Dashed vertical line boundary between microsaprophagous astigmatids (left) and macrosaprophagous astigmatids (right) from Figs. 20, 23a. Dotted diagonal line = approximate boundary between interstitial habit (upper and left) and surface habit (lower and right); see Fig. 23b; derived from difference in quadratic fits in sub-figure (a) and (b). Upper convex hull (macrophytophagous oribatid-like) subset of this consistent but atypical astigmatid design form comprising mainly glycyphagoids and pyroglyphoids (see Fig. 9). Lower convex hull subset of general astigmatid trophic design form shown by acaroids akin to microphytophagous/fragmentary feeding oribatids (note the ‘Typical’ astigmatid mite sits inside this group at the origin). Economically important acaroids are macro-saprophagous versions of the classic base design. Note the Winterschmidtiids are at the extreme lower left

**Fig. 26:**
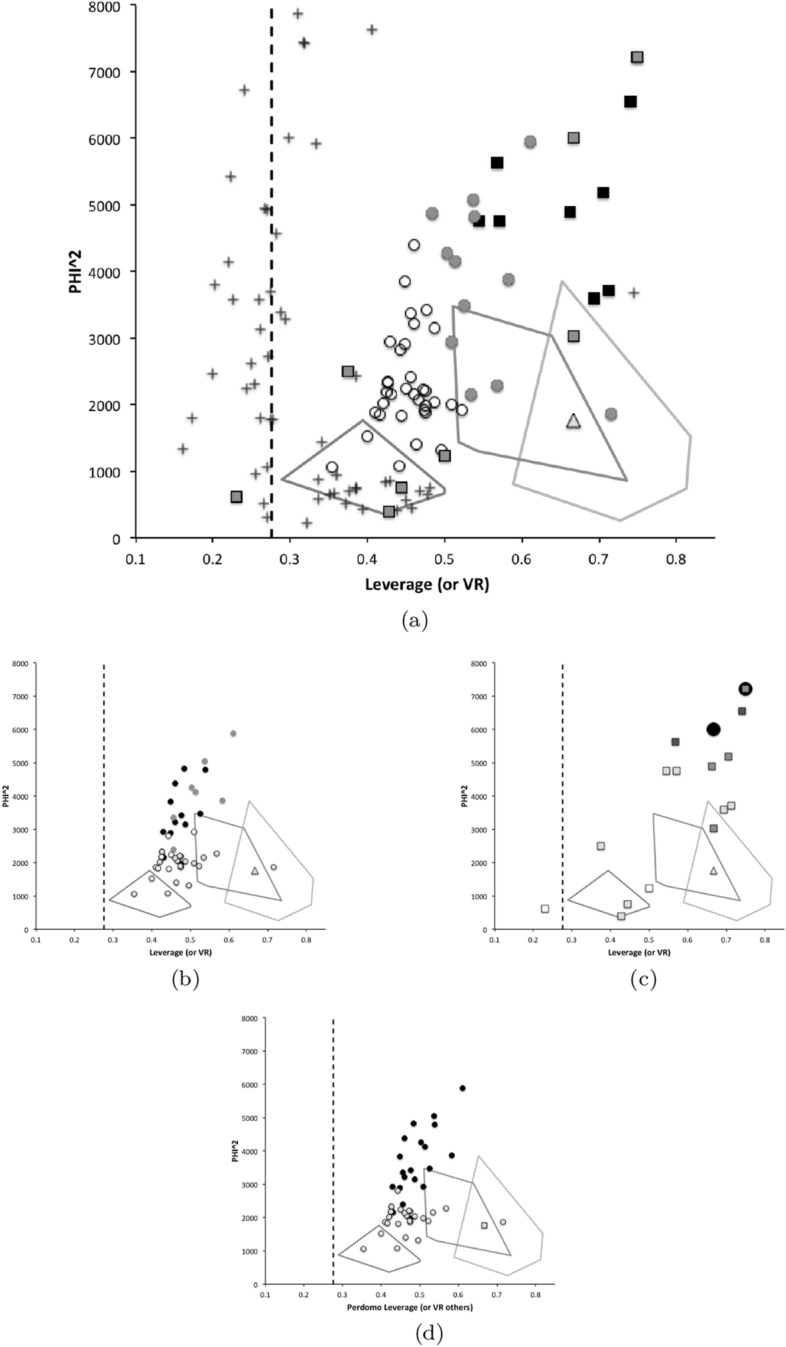
Summary over mite sub-orders. Uses convex hulls (left to right) encompassing the trophic regions confirmed with radioisotopes from Perdomo et al. (2012) where left polygon = oribatid ‘carnivores’, middle polygon = oribatid ‘secondary decomposers’, right polygon = oribatid ‘primary decomposers’ (Table 9). Velocity ratio (*VR*) used as ‘Leverage’ for species not examined radiologically. $PHI^2$ = surrogate for adductive tendon force *F*1. Dashed line at *VR* = 0.276 boundary between cutting killing-style mesostigmatid carnivores (to the left) and crushing killing-style mesostigmatid carnivores (to the right) from Bowman (2021). **a** All mites. Circles = Astigmata (this review). Closed grey circles = Astimata Upper group in Fig. 24c (includes also S5, TH3, TH4 from lower group species in Fig. 25c). Crosses = Mesostigmata from Bowman (2021). Triangle = Lichenivore (*Austrachipteria* sp. 1) from Perdomo et al. (2012). Black squares = Oribatids extracted from Schuster (1956). Grey squares = Oribatids extracted from Kaneko (1988). **b** Astigmatids only. Black circles: ‘Surface-omnivores’. Dark grey circles: ‘Interstitial omnivores’. Open circles: ‘Surface-fragmentary feeders’. Pale grey circles: ‘Interstitial fragmentary feeders’. **c** Oribatids only. Species from Schuster (1956) and Kaneko (1988). Open squares through to black circles in colour density: ‘Fragmentary’ < ‘Microphytophage’ < ‘Macrophytophage’ <’Non-specialised’ < ‘Panphytophage’. **d** Astigmatids only. Micro-saprophagous (pale grey circles) versus macro-saprophagous (black circles) mites (see Fig. 25c)

**Fig. 27:**
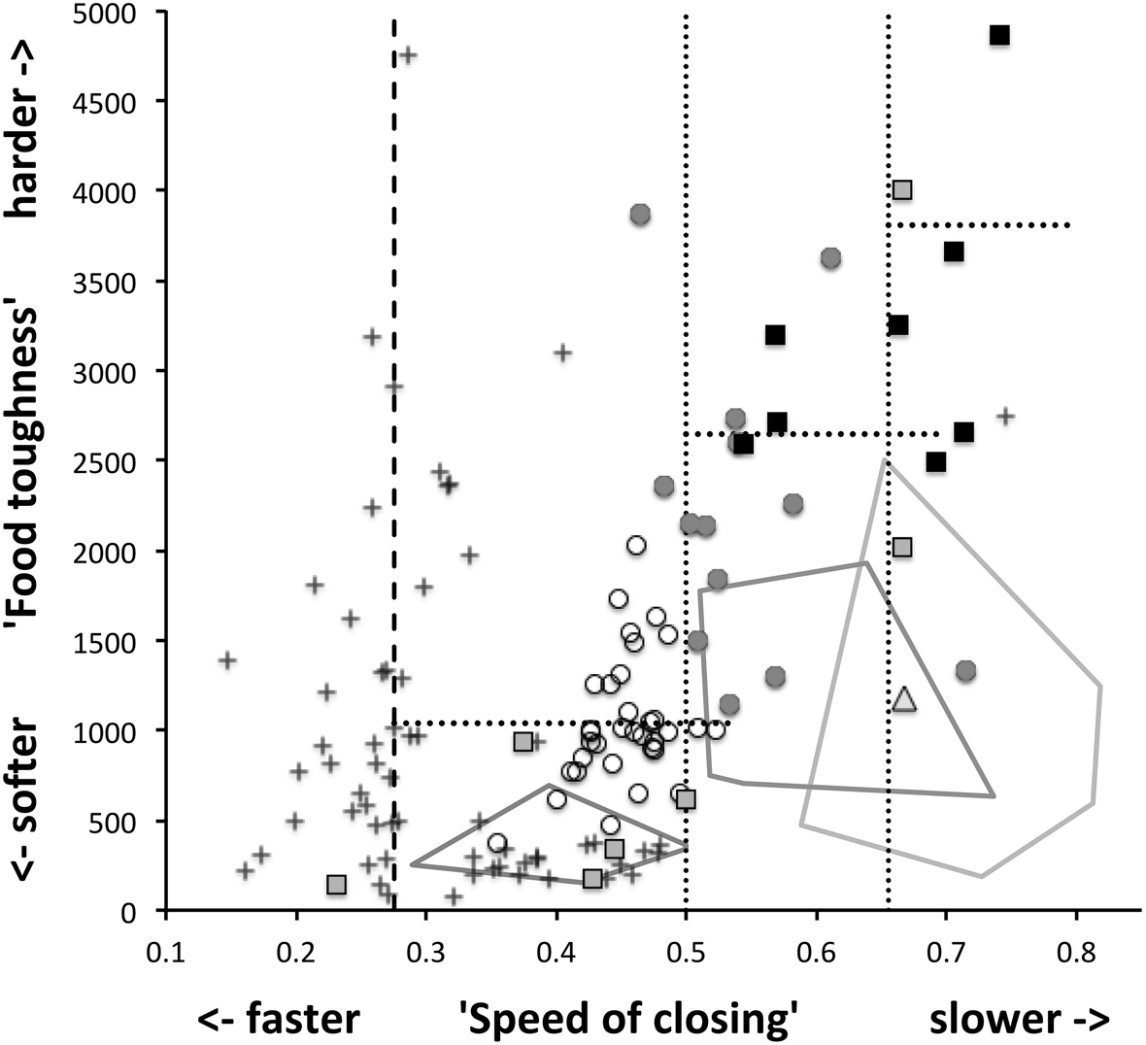
Synthesis of terminology over mite sub-orders based upon notional ‘speed of closing’ of cheliceral chela (surrogate $=$ leverage or velocity ratio as appropriate for data origin) versus ‘toughness of food’ (surrogate $= VR*PHI^2 \approx F2AV$). Convex hulls (left to right) encompass the trophic regions confirmed with radioisotopes from Perdomo et al. (2012) where left polygon = oribatid ‘carnivores’, middle polygon $=$ oribatid ‘secondary decomposers’, right polygon $=$ oribatid ‘primary decomposers’ (Table 9). Velocity ratio (*VR*) used as leverage for species not examined radiologically. Dashed line at $VR = 0.276$ boundary between cutting killing-style mesostigmatid carnivores (to the left) and crushing killing-style mesostigmatid carnivores (to the right) from Bowman (2021). The dotted vertical line at 0.5 is taken from the velocity ratio value of the cross-point in Fig. 24c. The vertical dotted line at 0.65 is calculated to be the midpoint threshold between Perdomo et al. (2012)’s primary (to the right) and secondary decomposers (to the left). It is close to the 0.6 used morphologically by Kaneko (1988) between his phytophagous types. Dotted horizontal lines (at $VR*PHI^{2}=1039.0, 2642.7, 3807.0$) are proposed boundaries for morphologically described feeding types (see text). Circles $=$ Astigmata (this review). Closed grey circles $=$ Astigmata in upper group in Fig. 24c (includes also *Suidasia pontifica* (S5), *Thyreophagus entomophagus* (TH3), *Thyreophagus* sp. (TH4) distinctive sub-design from lower group species in Fig. 25c). Crosses $=$ Mesostigmata from Bowman (2021). Triangle $=$ Lichenivore (*Austrachipteria* sp. 1) from Perdomo et al. (2012). Black squares $=$ Oribatids extracted from Schuster (1956). Grey squares $=$ Oribatids extracted from Kaneko (1988)

**Fig. 28:**
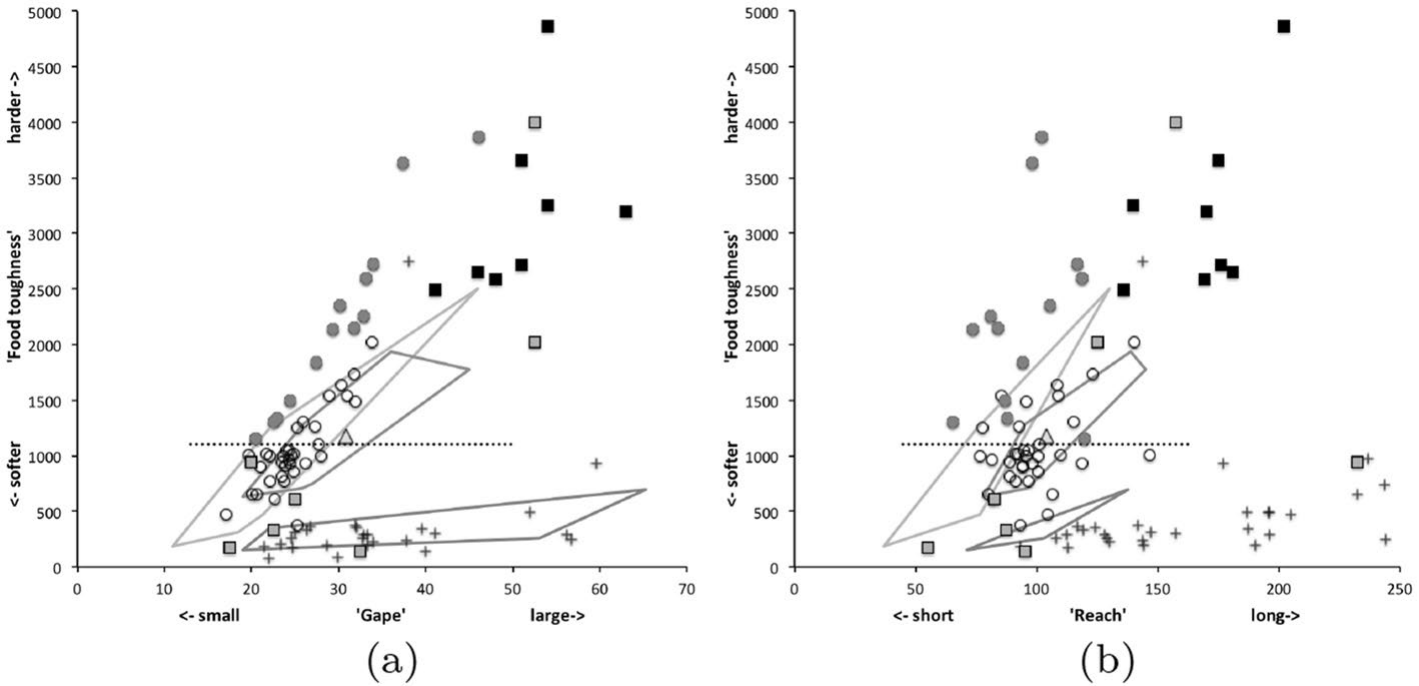
Comparative chelal gape **a** *L*2*M* or MOVLENGTH in $\mu$m, and cheliceral reach, **b** *CLI* or FIXLENGTH in $\mu$m, versus toughness of food (surrogate $= VR*PHI^2 \approx F2AV$). Convex hulls encompass the trophic regions confirmed with radioisotopes from Perdomo et al. (2012) where bottom polygon $=$ oribatid ‘carnivores’, other two overlapping polygons $=$ oribatid ‘secondary decomposers’ (slightly to right and down) and oribatid ‘primary decomposers’ (slightly to left and up)—see Table 9. Circles = Astigmata (this review). Closed grey circles = Astigmata Upper group in Fig. 24 (includes also *Suidasia pontifica* (S5), *Thyreophagus entomophagus* (TH3), *Thyreophagus* sp. (TH4) distinctive sub-design from lower group species in Fig. 25). Crosses $=$ Mesostigmata from Bowman (2021). Triangle $=$ Lichenivore (*Austrachipteria* sp. 1) from Perdomo et al. (2012). Black squares $=$ Oribatids extracted from Schuster (1956). Grey squares $=$ Oribatids extracted from Kaneko (1988). Dotted line (at $VR*PHI^{2}=1100.6$) is lowest ‘food toughness’ $\approx$ surrogate of chelal adductive force, for common recognised agricultural pest astigmatid species (*Acarus siro*, *Glycyphagus domesticus*, *Lepidoglyphus destructor*, *Rhizoglyphus echinopus*, *Tyrolichus casei*, *Tyrophagus longior* and *Tyrophagus nieswanderi*)

**Fig. 29:**
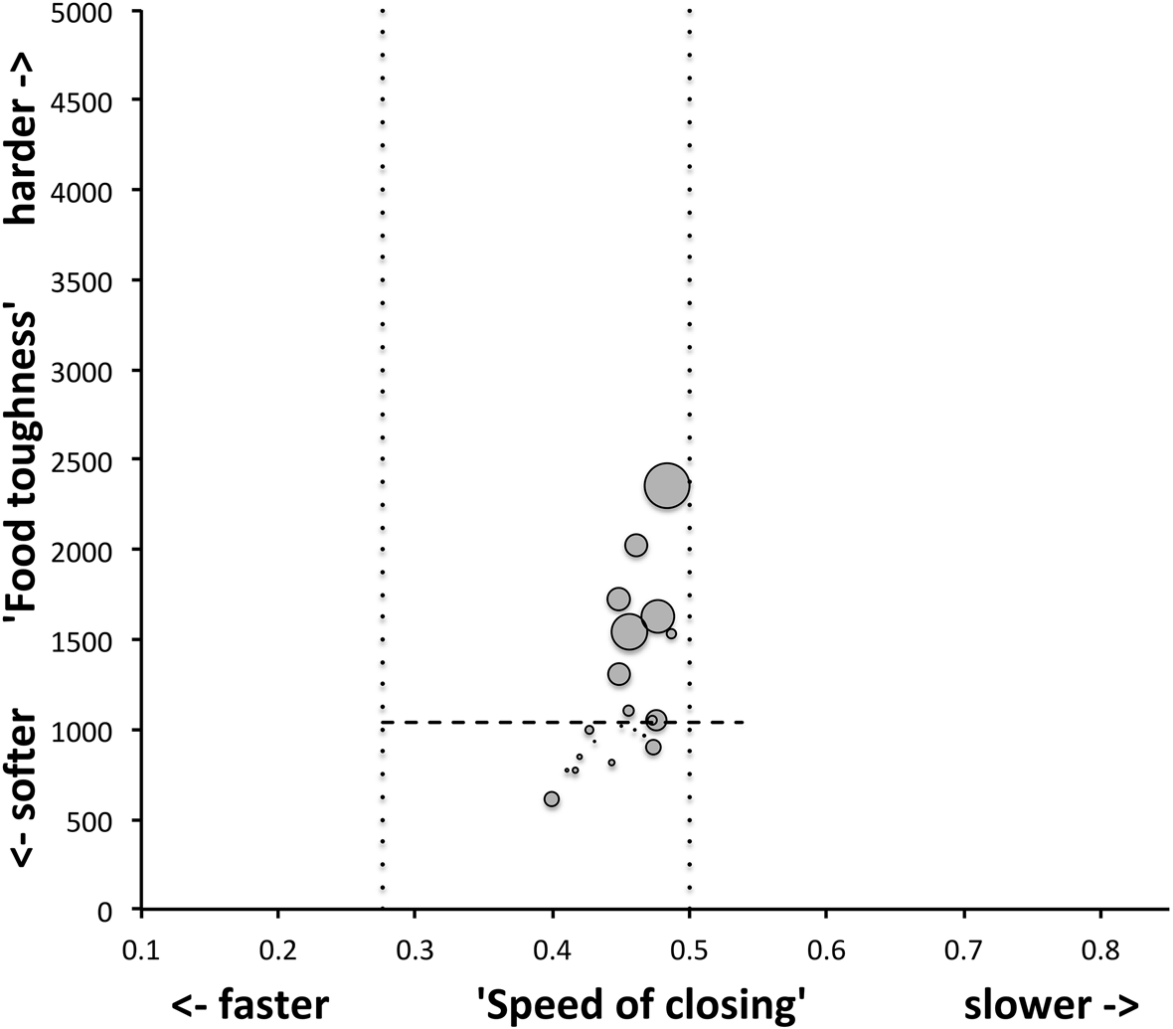
Future work. Notional speed of closing of cheliceral chela (surrogate $=$ velocity ratio) versus ‘toughness of food’ (surrogate $= VR*PHI^2 \approx F2AV$) as in Fig. 27 for those astigmatids having hide protease assays. Bubble size is activity in $\mu$g trypsin/mg BSA from Bowman (1981, 1987). Dashed threshold at $VR*PHI^{2}=1039.0$ (see text). The lower group are: *Acarus farris* (A17), *Acarus farris* (A1), *Tyrophagus palmarum* [‘A’] (T17), *Tyrophagus palmarum* [‘B’] (T32), *Tyrophagus robertsonae* (T7), *Tyrophagus perniciosus* [‘A’] (T8), *Tyrophagus putrescentiae* [‘A’] (T13), *Tyrophagus putrescentiae* [‘B’] (T9), *Tyrophagus similis* [‘A’] (T44), *Tyrophagus similis* [‘B’] (T21), and *Tyrophagus savasi* (T11). The upper group are: *Acarus gracilis* (A4), *Acarus siro* (A10b), *Acarus siro* [SW sp.] (A15), *Rhizoglyphus echinopus* (R2 ,R1), *Tyrophagus longior* (T40), *Tyrophagus nieswanderi* (T6), *Tyrophagus perniciosus* [‘B’] (T38), *Glycyphagus domesticus* (G5), and *Lepidoglyphus destructor* (G6)

**Table 5:**
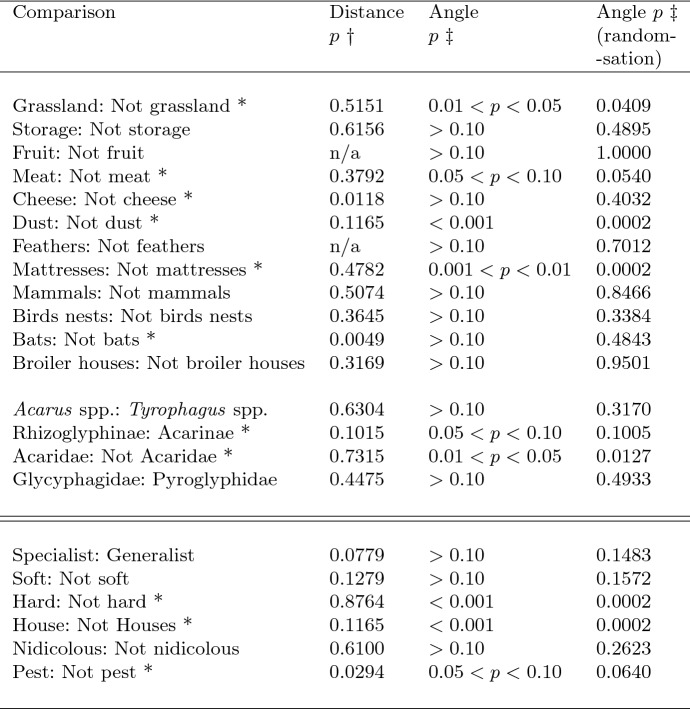
Statistical hypothesis tests for species’ trophic form in Fig. 5d and food habitat type, taxonomic position, and biome specialisation according to Hughes (1976) above double line

Hypothesis tests for extra subjective scoring by the review author below double line. See Figs. 15, 16, 17, 18 and 19, 28. Pest species: *Acarus siro* (A10B, A15), *Glycyphagus domesticus* (G5), *Lepidoglyphus destructor* (G6), *Rhizoglyphus echinopus* (R1, R2), *Tyrophagus nieswanderi* (T6), *Tyrophagus longior* (T40), *Tyrolichus casei* (T62). Non pest: remaining 38 taxa. †Welch Two Sample *t*-test (alternative hypothesis: true difference in means is not equal to 0). ‡Watson’s Two-Sample Test of Homogeneity. n/a Insufficient replication. *Result of interest

Are habitats associated with astigmatid form? Observed biological and ecological heat-maps for statistically important associations (see Table 5) are shown of: food type in Fig. 15, taxonomic families, sub-families and genera in Fig. 16, habitats in Figs. 17 and 18, and life strategies in Fig. 19. Heat-maps for each species and other measures over the same ordination are available from the author on request. However, no others were strongly informative.

Velocity ratio is not important in predicting pest status (Welch’s *t*-test $p = 0.1024$), rather crunch force might indicate agricultural importance. Herbivores (‘Grassland’ in Table 5) and pest species (statistically confirmed as of distinct design; Table 5) show high *F*2*AV* values (Fig. 14). However, due to high between species residual variation neither group shows significant elevations (Welch’s *t*-test $p=0.23$ and $p=0.13$, respectively). Rather, variation in velocity ratio (Fig. 6a) appears to be related to eating dust (Welch’s *t*-test $p=0.093$), the hardness of food (Fig. 15 Welch’s *t*-test $p=0.054$), the pyroglyphid taxonomic grouping (Fig. 16 but Welch’s *t*-test $p=0.16$), and living in mattresses (Fig. 17 Welch’s *t*-test $p=0.081$) or houses (Fig. 17 Welch’s *t*-test $p=0.093$).

Trophic correlates of fruit eating could not be tested (Table 5). However, *Carpoglyphus lactis* appears not to be of a fundamentally distinct design compared to the bulk of free-living astigmatids despite it’s low velocity ratio (0.355) for its tweezer-like chela and its obligate habitat restriction of figs, dates, dried fruits like sultanas and raisins (Evans et al. 1961; Munro 1966). Rather it has the consilient lowest crunch-force (*F*2*AV*). The genus *Carpoglyphus* is enigmatic (OConnor *pers. comm.*). *Carpoglyphus lactis* is not just restricted to fruits, in addition it has been found in various fermenting substrates (e.g., sap flows, fermented beverages, etc.) so this mite may actually feed largely on yeasts. A related species *Carpoglyphus wardleorum* has been found associated with mouldy honeydew (Clark 2010). This genus is most closely related to the Algophagidae, many species of which are restricted to sap flows.

There was a clear design correlate with meat and separately with cheese eating (Table 5, Fig. 15). The well-known cheese eating T62 *Tyrolichus casei*’s destructive power (Evans et al. 1961) is confirmed, it has a crunch force (*F*2*AV*, Table 2) comparable to that of *Acarus siro*. Dust eating (Table 5, Fig. 15) is partially associated with: high velocity ratios (Fig. 6 Welch’s *t*-test $p=0.093$), or being a pyroglyphid (Fig. 16), or eating hard food, or living in mattresses, or in houses. This all makes sense with respect to the known *Dermatophagoides* species feeding (Evans et al. 1961). Whilst there was no design correlate with eating soft food (Table 5), eating hard food is partially associated with: high velocity ratios (Fig. 6 Welch’s *t*-test $p=0.054$), or being a pyroglyphid (Fig. 16), or eating dust, or living in mattresses or houses, as one would expect. The trophic design of mites that live in mattresses (cf. Table 5) is partially associated with: the pyroglyphid form (Fig. 16), or a high chelal velocity ratio (Fig. 6 Welch’s *t*-test $p=0.081$), or eating dust, or the mite design that lives in houses (Fig. 17). Those designed for living in houses are partially associated with: the pyroglyphid design (Fig. 16), or a high chelal velocity ratio (Fig. 6 Welch’s *t*-test $p=0.093$), or eating dust, or the mite design that lives in mattresses (Fig. 17). All of these thus make a fully congruent set i.e., each dimension captures the same information without contradiction. The pyroglyphid adaptive syndrome is coherent. It is tempting to conclude that such mites are adapted for coprophagy and necrophagy, lifestyles rare in oribatids (Wallwork 1983). Such material could be highly nutritious if somewhat hard on drying out (like an Egyptian ‘mummy’). If pyroglyphids are small size necrophages/coprophages, one would expect them to be species in general who dig in and ‘demolish’ this material (like the head of a long-necked carrion-feeding vulture, a dermestid ‘bacon beetle’ or a carcass-burrowing hyena).

No obvious trophic correlate with being associated with bats was clear (Fig. 18). It is not clear how to interpret the recorded habitat with respect to *Acarus gracilis* and *Glycyphagus domesticus* and bats (Table 5). More field sampling of bat roosts is needed. The span of species used in ecomorphological studies is important (Bowman 2021).

The lack of a significant result for mites of storage (versus not storage) was a surprise. However, inspection of Fig. 18 nicely shows that there are two separate clusters of storage related designs typified by the ‘East’ and the ‘West’ but not the middle nor the ‘North’ or ‘South’. This is derived from the locations on the ordination (Fig. 5d) of the particular mixture of mite families used in this review (see Fig. 16).

The premise of a food access (i.e., size and reach) plus a morsel handling (i.e., gape and crunch force) trophic framework defining the adaptive syndromes of astigmatid functional groups (posed as in models 1. and 2. in the “Expected results” section above) is vindicated.

### Have astigmatid species evolved differently to avoid resource competition?

As Hurlbutt (1968) states “Two species having the same ecology cannot persist in the same region”, i.e., complete competitors cannot co-exist. Yet, Krantz and Lindquist (1979) describe frequent astigmatid co-existence. Hutchinson (1959)’s rule is the observation that the trophic structures (i.e., mouths) of sympatric congeneric species (*in competition with each other*) generally vary by a factor of 1.1–1.4. The rule’s legitimacy has been questioned (Roth 1981) and other categories of objects also exhibit roughly similar 1.3 size ratios (see Eadie and Broekhoven 1987). This trophic variation is assumed to lead to niche differentiation, and thus allow the coexistence of multiple similar species in the same habitat, by the partitioning of food resources. Care needs to be taken in any interpretation if it is to be used to infer detailed community structure (Wiens and Rotenberry 1981). Herein the checks using size (idiosomal index *IL*; Tables 3 and 4) are for just the first step in any simple explanation of any co-occurrence of gross morphology amongst the astigmatids. It is used here only as an initial rough first-pass filter to see if further detailed investigations are warranted. A factor of 1.3 in linear dimensions infers about a doubling of overall size by volume or weight.

A habitat cross-classification drawn up from those in Hughes (1976) (see “Materials and methods”) was sparse with little clustering (just as in phrynosomatid lizards; Herrel et al. 2002). Out of the 37 astigmatids classified into 24 ‘biomes’, five taxa (*Suidasia pontifica* (S5), *Tyrophagus putrescentiae* [‘B’] (T9), *Tyrophagus putrescentiae* [‘A’] (T13), *Tyrophagus perniciosus* [‘A’] (T8), and *Tyrophagus perniciosus* [‘B’] (T38)) fall together as Biome 1 - ‘cheese and storage habitat, not-nidicolous not-houses soft food specialists’, three taxa (*Acarus immobilis* (A1), *Tyrophagus palmarum* [‘A’] (T17) and, *Tyrophagus palmarum* [’B’] (T32)) fall together as Biome 2—‘grassland & cheese, birds-nest nidicolous (but) not-houses soft food specialists’, and three taxa (*Rhizoglyphus echinopus* both samples R1 and R2, and *Tyrophagus nieswanderi* (T6)) fall together as Biome 3—‘grassland, not-houses not-nidicolous soft food specialists’. The remaining cross-classifications as biomes represent just one or two taxa (*results not shown*). Trying to borrow information from species by smoothing over the ordination to see overall patterns in astigmatid trophism is thus justified for at least 12 of the 47 taxa studied. Taking each of these three ‘biome’ groupings as sets of competitive sympatric congeneric species, would the design of mites within each potentially impact competition for resources between them in that biome (as has been found for food collection specialisations in fish; Fryer and Iles 1972)? Are there differences of form within each biome to avoid species elimination or natural selection acting in some other way to avoid astigmatid competition?

Table 3 appears to show some empirical evidence of Hutchinson’s ratio in body size stepping from species to species (i.e., in 60% of the comparisons). On the face of it, Hughes (1976)’s habitat classification, whilst not perfect, appears useful. However, these size differences for coexistence of taxa are then statistically checked in two ways in the Table: whether there is a minimum size ratio compatible with coexistence of ecologically similar species (test “1” in Simberloff and Boecklen 1981), and whether three or more ecologically similar co-existing species tend to have constant size ratio between species adjacent in size-ranking (test “2” in Simberloff and Boecklen 1981). This check is whether the species’ sizes are reasonably viewed as non-random and not independent i.e., the null $H_0$ is that the ratio of sizes is random, the alternative $H_1$ is that they are regularly spaced.

**Table 6:** Reviewed astigmatids ordered by trophic type from heuristic modelling within super-family, family and sub-family using terms from Bowman (2021). Note blocked patterns

Super-family	Family	Sub-family	Species	Code	Mite is.	Food is at a	Which is relatively ...body	Food is .	Requiring a .	Chela takes .	In .	With oral area being.
Acaroidea	Acaridae	Acarinae	*Tyrophagus longior*	T40	Large	Long Range	Close to	Hard	Feeble effort	Small food	Little chunks	Tiny mouthfulls
Acaroidea	Acaridae	Acarinae	*Kuzinia laevis*	KL or $	Large	Long Range	Close to	Hard	Powerful grip	Big food	Major grab	Well stuffed
Acaroidea	Acaridae	Acarinae	*Tyrophagus vanheurni*	T7	Large	Long Range	Close to	Soft	Feeble effort	Small food	Little chunks	Tiny mouthfulls
Acaroidea	Acaridae	Acarinae	*Tyrolichus casei*	T62	Large	Long Range	Well away	Hard	Powerful grip	Big food	Major grab	Tiny mouthfulls
Acaroidea	Acaridae	Acarinae	*Tyrophagus perniciosus* [’B’]	T38	Large	Long Range	Well away	Hard	Powerful grip	Big food	Major grab	Tiny mouthfulls
Acaroidea	Acaridae	Acarinae	*Acarus siro* [’H’]	A10b	Large	Long Range	Well away	Hard	Powerful grip	Big food	Major grab	Well stuffed
Acaroidea	Acaridae	Acarinae	*Tyroborus lini*	T66	Large	Long Range	Well away	Hard	Powerful grip	Big food	Major grab	Well stuffed
Acaroidea	Acaridae	Acarinae	*Tyrophagus perniciosus* [’A’]	T8	Large	Long Range	Well away	Soft	Feeble effort	Big food	Major grab	Tiny mouthfulls
Acaroidea	Acaridae	Acarinae	*Acarus immobilis*	A1	Large	Short distance	Close to	Soft	Feeble effort	Small food	Little chunks	Tiny mouthfulls
Acaroidea	Acaridae	Acarinae	*Tyrophagus brevicrinatus*	T89 or TB	Large	Short distance	Close to	Soft	Feeble effort	Small food	Little chunks	Tiny mouthfulls
Acaroidea	Acaridae	Acarinae	*Tyrophagus palmarum* [’A’]	T17	Large	Short distance	Close to	Soft	Feeble effort	Small food	Little chunks	Tiny mouthfulls
Acaroidea	Acaridae	Acarinae	*Tyrophagus similis* [’B’]	T21	Large	Short distance	Close to	Soft	Feeble effort	Small food	Little chunks	Tiny mouthfulls
Acaroidea	Suidasiidae	Acarinae	*Neosuidasia* sp.	LA1	Small	Long Range	Well away	Hard	Powerful grip	Big food	Major grab	Tiny mouthfulls
Acaroidea	Acaridae	Acarinae	*Aleuroglyphus ovatus*	AL2	Small	Long Range	Well away	Hard	Powerful grip	Big food	Major grab	Well stuffed
Acaroidea	Acaridae	Acarinae	*Acarus siro* [SW sp.]	A15	Small	Long Range	Well away	Soft	Feeble effort	Big food	Major grab	Well stuffed
Acaroidea	Acaridae	Acarinae	*Acarus chaetoxysilos*	AC204	Small	Short distance	Close to	Soft	Feeble effort	Small food	Little chunks	Tiny mouthfulls
Acaroidea	Acaridae	Acarinae	*Forcellinia galleriella*	F1	Small	Short distance	Close to	Soft	Feeble effort	Small food	Little chunks	Tiny mouthfulls
Acaroidea	Acaridae	Acarinae	*Tyrophagus nieswanderi*	T6	Small	Short distance	Close to	Soft	Feeble effort	Small food	Little chunks	Tiny mouthfulls
Acaroidea	Acaridae	Acarinae	*Tyrophagus putrescentiae* [’B’]	T9	Small	Short distance	Close to	Soft	Feeble effort	Small food	Little chunks	Tiny mouthfulls
Acaroidea	Acaridae	Acarinae	*Tyrophagus robertsonae*	T87	Small	Short distance	Close to	Soft	Feeble effort	Small food	Little chunks	Tiny mouthfulls
Acaroidea	Acaridae	Acarinae	*Tyrophagus tropicus*	T90	Small	Short distance	Close to	Soft	Feeble effort	Small food	Little chunks	Tiny mouthfulls
Acaroidea	Acaridae	Acarinae	*Acarus farris*	A17	Small	Short distance	Close to	Soft	Feeble effort	Small food	Little chunks	Well stuffed
Acaroidea	Acaridae	Acarinae	*Madaglyphus legendrei*	T34	Small	Short distance	Well away	Soft	Feeble effort	Small food	Little chunks	Tiny mouthfulls
Acaroidea	Acaridae	Acarinae	*Tyrophagus palmarum* [’B’]	T32	Small	Short distance	Well away	Soft	Feeble effort	Small food	Little chunks	Tiny mouthfulls
Acaroidea	Acaridae	Acarinae	*Tyrophagus similis* [’A’]	T44	Small	Short distance	Well away	Soft	Feeble effort	Small food	Little chunks	Tiny mouthfulls
Acaroidea	Acaridae	Acarinae	*Acarus gracilis*	A4	Small	Short distance	Well away	Soft	Feeble effort	Small food	Major grab	Tiny mouthfulls
Acaroidea	Acaridae	Acarinae	*Tyrophagus putrescentiae* [’A’]	T13	Small	Short distance	Well away	Soft	Feeble effort	Small food	Major grab	Tiny mouthfulls
Acaroidea	Acaridae	Acarinae	*Tyrophagus savasi*	T11	Small	Short distance	Well away	Soft	Feeble effort	Small food	Major grab	Tiny mouthfulls
Acaroidea	Acaridae	Rhizoglyphinae	*Sancassania berlesei*	C3	Large	Long Range	Close to	Hard	Feeble effort	Big food	Little chunks	Tiny mouthfulls
Acaroidea	Acaridae	Rhizoglyphinae	*Thyreophagus* sp.	TH4	Large	Long Range	Close to	Hard	Feeble effort	Big food	Little chunks	Tiny mouthfulls
Acaroidea	Acaridae	Rhizoglyphinae	*Rhizoglyphus echinopus*	R2	Large	Long Range	Close to	Hard	Powerful grip	Big food	Little chunks	Tiny mouthfulls
Acaroidea	Acaridae	Rhizoglyphinae	*Rhizoglyphus echinopus*	R1	Large	Long Range	Close to	Hard	Powerful grip	Big food	Little chunks	Well stuffed
Acaroidea	Acaridae	Rhizoglyphinae	*Thyreophagus entomophagus*	TH3	Large	Short distance	Close to	Hard	Powerful grip	Small food	Little chunks	Tiny mouthfulls
Acaroidea	Acaridae	Rhizoglyphinae	*Cosmoglyphus oudemansi*	C10	Large	Short distance	Close to	Soft	Feeble effort	Small food	Little chunks	Tiny mouthfulls
Acaroidea	Acaridae	Rhizoglyphinae	*Cosmoglyphus hughesae*	C5	Small	Short distance	Close to	Soft	Feeble effort	Small food	Little chunks	Tiny mouthfulls
Acaroidea	Lardoglyphidae	-	*Lardoglyphus zacheri*	L3	Large	Long Range	Close to	Soft	Feeble effort	Small food	Little chunks	Tiny mouthfulls
Acaroidea	Lardoglyphidae	-	*Lardoglyphus konoi*	L1	Small	Short distance	Close to	Soft	Feeble effort	Small food	Little chunks	Tiny mouthfulls
Acaroidea	Suidasiidae	Suidasiinae	*Suidasia pontifica*	S5	Small	Short distance	Close to	Soft	Feeble effort	Small food	Little chunks	Tiny mouthfulls
Glycyphagoidea	Aeroglyphidae	-	*Glycycometus hughesae*	G3	Small	Long Range	Well away	Hard	Powerful grip	Big food	Major grab	Well stuffed
Glycyphagoidea	Chortoglyphidae	-	*Chortoglyphus arcuatus*	CH1	Small	Long Range	Well away	Hard	Powerful grip	Big food	Major grab	Well stuffed
Glycyphagoidea	Glycyphagidae	Glycyphaginae	*Lepidoglyphus destructor*	G6	Large	Long Range	Close to	Hard	Powerful grip	Big food	Major grab	Well stuffed
Glycyphagoidea	Glycyphagidae	Glycyphaginae	*Glycyphagus domesticus*	G5	Small	Long Range	Well away	Hard	Powerful grip	Big food	Major grab	Well stuffed
Hemisarcoptoidea	Carpoglyphidae	-	*Carpoglyphus lactis*	Ca4	Small	Short distance	Close to	Soft	Feeble effort	Small food	Major grab	Well stuffed
Hemisarcoptoidea	Winterschmidtiidae	-	“Winterschmidtiidae sp.”	CV1(66)	Small	Short distance	Close to	Soft	Feeble effort	Small food	Little chunks	Tiny mouthfulls
Pyroglyphoidea	Pyroglyphidae	Dematophagoidinae	*Dermatophagoides farinae*	D4	Small	Short distance	Well away	Hard	Powerful grip	Big food	Major grab	Well stuffed
Pyroglyphoidea	Pyroglyphidae	Dematophagoidinae	*Dermatophagoides microceras*	D5	Small	Short distance	Well away	Soft	Powerful grip	Small food	Major grab	Well stuffed
Pyroglyphoidea	Pyroglyphidae	Dematophagoidinae	*Dermatophagoides pteronyssinus*	D3	Small	Short distance	Well away	Soft	Powerful grip	Small food	Major grab	Well stuffed
			Typical		Small	Short distance	Close to	Soft	Feeble effort	Small food	Little chunks	Tiny mouthfulls

**Table 7:** Standardised verbal summary over astigmatid species reviewed based upon heuristics

Species	Culture number	Summary
*Acarus chaetoxysilos *Griffiths	AC204	Interstitial. Potential cavity-living, substratum browsing/gleaning generalist. Only small and soft food morsels (Fragmentary feeders). Burrowing picker, Selective burrower, Squasher, Soft slicer or ‘Plankton’ feeder
*Acarus farris* (Oudemans)	A17	Interstitial. Potential cavity-living, substratum browsing/gleaning generalist. Only small and soft food morsels (Fragmentary feeders). Burrowing picker, Selective burrower, Squasher, Soft slicer or ‘Plankton’ feeder
*Acarus gracilis* Hughes	A4	Interstitial. Potential cavity-living, substratum browsing/gleaning generalist. Only small and soft food morsels (Fragmentary feeders). Burrowing picker, Selective burrower, Squasher, Soft slicer or ‘Plankton’ feeder
*Acarus immobilis* Griffiths	A1	Surface-living, substratum browsing/gleaning generalist. Only small and soft food morsels (Fragmentary feeders). Picker, Squasher, Soft slicer or ‘Plankton’ feeder
*Acarus siro* L. [‘H’]	A10b	Surface-living, potential crevice feeding/excavating specialist. Potential specialist for large (and small), hard (and soft) food morsels (Pan-saprophages). Chunk or Demolition feeder, Nutcracker or Cruncher
*Acarus siro* L. [SW sp.]	A15	Interstitial. Potential cavity-living, possible crevice feeding/excavating specialist. Potential specialist for large but must be soft food morsels (Macro-saprophages). Wide-mouthed burrower, Squasher, Soft slicer or ‘Plankton’ feeder
*Aleuroglyphus ovatus* (Troupeau)	AL2	Interstitial. Potential cavity-living, possible crevice feeding/excavating specialist. Potential specialist for large (and small) and hard (and soft) food morsels (Pan-saprophages). Burrowing Cruncher
*Sancassania berlesei* (Michael)	C3	Surface-living, potential crevice feeding/excavating specialist. Potential specialist for large (and small), hard (and soft) food morsels (Pan-saprophages). Chunk or Demolition feeder, Nutcracker or Cruncher
*Cosmoglyphus oudemansi* Zachvatkin	C10	Surface-living, substratum browsing/gleaning generalist. Only small and soft food morsels (Fragmentary feeders). Picker, Squasher, Soft slicer or ‘Plankton’ feeder
*Cosmoglyphus hughesae* Samsinak]	C5	Interstitial. Potential cavity-living, substratum browsing/gleaning generalist. Only small and soft food morsels (Fragmentary feeders). Burrowing picker, Selective burrower, Squasher, Soft slicer or ‘Plankton’ feeder
*Kuzinia laevis* (Dujardin)	KL or $	Surface-living, potential crevice feeding/excavating specialist. Potential specialist for large (and small), hard (and soft) food morsels (Pan-saprophages). Chunk or Demolition feeder, Nutcracker or Cruncher. Known from Bumblebees (Schwarz and Huck (1997))
*Lardoglyphus konoi* (Sasa and Asanuma)	L1	Interstitial. Potential cavity-living, substratum browsing/gleaning generalist. Only small and soft food morsels (Fragmentary feeders). Burrowing picker, Selective burrower, Squasher, Soft slicer or ‘Plankton’ feeder
*Lardoglyphus zacheri* Oudemans	L3	Surface-living, potential crevice feeding/excavating specialist. Only small and soft food morsels (Fragmentary feeders). Picker, Squasher, Soft slicer or ‘Plankton’ feeder
*Neosuidasia* sp.	LA1	Interstitial. Potential cavity-living, possible crevice feeding/excavating specialist. Potential specialist for large (and small) and hard (and soft) food morsels (Pan-saprophages). Burrowing Cruncher
*Madaglyphus legendrei* (Fain)	T34	Interstitial. Potential cavity-living, substratum browsing/gleaning generalist. Only small and soft food morsels (Fragmentary feeders). Burrowing picker, Selective burrower, Squasher, Soft slicer or ‘Plankton’ feeder
*Rhizoglyphus echinopus* (Fumouze and Robin)	R2	Surface-living, potential crevice feeding/excavating specialist. Potential specialist for large (and small), hard (and soft) food morsels (Pan-saprophages). Chunk or Demolition feeder, Nutcracker or Cruncher
*Rhizoglyphus echinopus* (Fumouze and Robin)	R1	Surface-living, potential crevice feeding/excavating specialist. Potential specialist for large (and small), hard (and soft) food morsels (Pan-saprophages). Chunk or Demolition feeder, Nutcracker or Cruncher
*Suidasia pontifica* Oudemans	S5	Interstitial. Potential cavity-living, substratum browsing/gleaning generalist. Only small and soft food morsels (Fragmentary feeders). Burrowing picker, Selective burrower, Squasher, Soft slicer or ‘Plankton’ feeder
*Thyreophagus* sp.	TH4	Surface-living, potential crevice feeding/excavating specialist. Potential specialist for large (and small), hard (and soft) food morsels (Pan-saprophages). Chunk or Demolition feeder, Nutcracker or Cruncher. Possible associated with springtails (Fain and Johnston (1974)), also see Michael (1885)
*Thyreophagus entomophagus* (Laboulbene)	TH3	Surface-living, substratum browsing/gleaning generalist. Only small morsels but potential specialist for hard (and soft) food (Micro-saprophages). Picker or ‘Plankton’ feeder, Nutcracker or Cruncher
*Tyroborus lini* Oudemans	T66	Surface-living, potential crevice feeding/excavating specialist. Potential specialist for large (and small), hard (and soft) food morsels (Pan-saprophages). Chunk or Demolition feeder, Nutcracker or Cruncher
*Tyrolichus casei* Oudemans	T62	Surface-living, potential crevice feeding/excavating specialist. Potential specialist for large (and small), hard (and soft) food morsels (Pan-saprophages). Chunk or Demolition feeder, Nutcracker or Cruncher
*Tyrophagus brevicrinatus* Robertson	T89 or TB	Surface-living, substratum browsing/gleaning generalist. Only small and soft food morsels (Fragmentary feeders). Picker, Squasher, Soft slicer or ‘Plankton’ feeder
*Tyrophagus longior* (Gervais)	T40	Surface-living, potential crevice feeding/excavating specialist. Only small morsels but potential specialist for hard (and soft) food (Micro-saprophages). Picker or ‘Plankton’ feeder, Nutcracker or Cruncher
*Tyrophagus nieswanderi* Johnston and Bruce	T6	Interstitial. Potential cavity-living, substratum browsing/gleaning generalist. Only small and soft food morsels (Fragmentary feeders). Burrowing picker, Selective burrower, Squasher, Soft slicer or ‘Plankton’ feeder
*Tyrophagus palmarum* Oudemans [‘A’]	T17	Surface-living, substratum browsing/gleaning generalist. Only small and soft food morsels (Fragmentary feeders). Picker, Squasher, Soft slicer or ‘Plankton’ feeder
*Tyrophagus palmarum* Oudemans [B]	T32	Interstitial. Potential cavity-living, substratum browsing/gleaning generalist. Only small and soft food morsels (Fragmentary feeders). Burrowing picker, Selective burrower, Squasher, Soft slicer or ‘Plankton’ feeder
*Tyrophagus vanheurni* Oudemans]	T7	Surface-living, potential crevice feeding/excavating specialist. Only small and soft food morsels (Fragmentary feeders). Picker, Squasher, Soft slicer or ‘Plankton’ feeder
*Tyrophagus perniciosus* Zachvatkin [‘A’]	T8	Surface-living, potential crevice feeding/excavating specialist. Potential specialist for large (and small) but must be soft food morsels (Macro-saprophages). Chunk or Demolition feeder, Squasher, Soft slicer or ‘Plankton’ feeder
*Tyrophagus perniciosus* Zachvatkin [B]	T38	Surface-living, potential crevice feeding/excavating specialist. Potential specialist for large (and small), hard (and soft) food morsels (Pan-saprophages). Chunk or Demolition feeder, Nutcracker or Cruncher
*Tyrophagus putrescentiae* (Schrank) [‘A’]	T13	Interstitial. Potential cavity-living, substratum browsing/gleaning generalist. Only small and soft food morsels (Fragmentary feeders). Burrowing picker, Selective burrower, Squasher, Soft slicer or ‘Plankton’ feeder
*Tyrophagus putrescentiae* (Schrank) [B]	T9	Interstitial. Potential cavity-living, substratum browsing/gleaning generalist. Only small and soft food morsels (Fragmentary feeders). Burrowing picker, Selective burrower, Squasher, Soft slicer or ‘Plankton’ feeder
*Tyrophagus robertsonae* Lynch	T87	Interstitial. Potential cavity-living, substratum browsing/gleaning generalist. Only small and soft food morsels (Fragmentary feeders). Burrowing picker, Selective burrower, Squasher, Soft slicer or ‘Plankton’ feeder.
*Tyrophagus similis* Volgin [‘A’]	T44	Interstitial. Potential cavity-living, substratum browsing/gleaning generalist. Only small and soft food morsels (Fragmentary feeders). Burrowing picker, Selective burrower, Squasher, Soft slicer or ‘Plankton’ feeder
*Tyrophagus similis* Volgin [B]	T21	Surface-living, substratum browsing/gleaning generalist. Only small and soft food morsels (Fragmentary feeders). Picker, Squasher, Soft slicer or ‘Plankton’ feeder
*Tyrophagus savasi* Lynch	T11	Interstitial. Potential cavity-living, substratum browsing/gleaning generalist. Only small and soft food morsels (Fragmentary feeders). Burrowing picker, Selective burrower, Squasher, Soft slicer or ‘Plankton’ feeder
*Tyrophagus tropicus* Robertson	T90	Interstitial. Potential cavity-living, substratum browsing/gleaning generalist. Only small and soft food morsels (Fragmentary feeders). Burrowing picker, Selective burrower, Squasher, Soft slicer or ‘Plankton’ feeder
*Carpoglyphus lactis* (L.)	Ca4	Interstitial. Potential cavity-living, substratum browsing/gleaning generalist. Only small and soft food morsels (Fragmentary feeders). Burrowing picker, Selective burrower, Squasher, Soft slicer or ‘Plankton’ feeder
*Chortoglyphus arcuatus* (Troupeau)	CH1	Interstitial. Potential cavity-living, possible crevice feeding/excavating specialist. Potential specialist for large (and small) and hard (and soft) food morsels (Pan-saprophages). Burrowing Cruncher
*Glycycometus hughesae* (Fain)	G3	Interstitial. Potential cavity-living, possible crevice feeding/excavating specialist. Potential specialist for large (and small) and hard (and soft) food morsels (Pan-saprophages). Burrowing Cruncher
*Lepidoglyphus destructor* (Schrank)	G6	Surface-living, potential crevice feeding/excavating specialist. Potential specialist for large (and small), hard (and soft) food morsels (Pan-saprophages). Chunk or Demolition feeder, Nutcracker or Cruncher
*Glycyphagus domesticus* (De Geer)	G5	Interstitial. Potential cavity-living, possible crevice feeding/excavating specialist. Potential specialist for large (and small) and hard (and soft) food morsels (Pan-saprophages). Burrowing Cruncher
*Dermatophagoides farinae* Hughes	D4	Interstitial. Potential cavity-living, substratum browsing/gleaning generalist. Potential specialist for large (and small) and hard (and soft) food morsels (Pan-saprophages). Wide-mouthed burrower, Burrowing Cruncher
*Dermatophagoides microceras* Griffiths and Cunnington	D5	Interstitial. Potential cavity-living, substratum browsing/gleaning generalist. Only small and soft food morsels (Fragmentary feeders). Burrowing picker, Selective burrower, Squasher, Soft slicer or ‘Plankton’ feeder
*Dermatophagoides pteronyssinus* (Trouessart)	D3	Interstitial. Potential cavity-living, substratum browsing/gleaning generalist. Only small and soft food morsels (Fragmentary feeders). Burrowing picker, Selective burrower, Squasher, Soft slicer or ‘Plankton’ feeder
“Winterschmidtiidae sp.”	CV1(66)	Interstitial. Potential cavity-living, substratum browsing/gleaning generalist. Only small and soft food morsels (Fragmentary feeders). Burrowing picker, Selective burrower, Squasher, Soft slicer or ‘Plankton’ feeder
*Forcellinia galleriella* Womersley	F1	Interstitial. Potential cavity-living, substratum browsing/gleaning generalist. Only small and soft food morsels (Fragmentary feeders). Burrowing picker, Selective burrower, Squasher, Soft slicer or ‘Plankton’ feeder

Capital letters in square brackets [...] represent isolated breeding groups (Griffiths 1979). Updated names after Barry OConnor (pers. comm.). ‘Plankton’ indicates microbes, spores, yeasts, algae, pollen grains, hyphae, rotifers, small nematodes etc. Unique types are: A15, TH3, T40, T8, and D4

For the first biome in Table 3: *Tyrophagus perniciosus* [‘A’] (T8) and *Tyrophagus perniciosus* [‘B’] (T38) have different trophic facies which are different to the other three taxa (Tables 6, 7). Although *Suidasia pontifica* (S5), *Tyrophagus putrescentiae* [’B’] (T9) and *Tyrophagus putrescentiae* [’A’] (T13) appear designed broadly similarly (Tables 6, 7), they vary in relative sizes (S5:T13 = 1.380, T13:T9 = 1.211). However, this pattern is not statistically different from random. All five species could thus not avoid resource competition if sympatric. *Tyrophagus perniciosus* [‘A’] (T8) and *Tyrophagus perniciosus* [‘B’] (T38) might not even be breeding groups of *Tyrophagus perniciosus* of course. *Tyrophagus perniciosus* [‘A’] (T8) is definitely *Tyrophagus perniciosus*; OConnor *pers. comm.*, but perhaps the identification of *Tyrophagus pernciosus* ‘B’] (T38) should be checked following the revision of *Tyrophagus* by Fan and Zhang (2007) before making biological conclusions about it?

For the second biome in Table 3: *Acarus immobilis* (A1) and *Tyrophagus palmarum* [’A’] (T17) have exactly the same categorisations in Tables 6, 7. Their relative sizes (in Table 2) of 225.81 and 216.86 $\mu$m, respectively, infers a relative size ratio of 1.041. Nevertheless for this set of species this pattern is not statistically different from random. *Tyrophagus palmarum* [‘B’] (T32) is distinct in trophic style from *Acarus immobilis* (A1) and *Tyrophagus palmarum* [‘A’] (T17), having very different facies from each other (Tables 6, 7) so perhaps could coexist. However, on this morphometric evidence all three species could not avoid resource competition if sympatric.

For the third biome in Table 3: the two instances of *Rhizoglyphus echinopus* R1, R2, have similar categorisation in Table 7, their design being only different in size—Table 4 (Welch’s *t*-test $p=0.045$), and perhaps oral ’greediness (Table 6). These two look more like distinct ‘cultivars’ (using a plant breeding analogy). Perhaps their size differences may be related to their different times in culture? The facies for *Tyrophagus nieswanderi* T6 is markedly distinct trophically from them (Tables 6, 7). Perhaps this is why its ratio in Table 3 to *Rhizoglyphus echinopus* can be seen to be essentially one. Moreover, there is evidence that these set of species are regularly spaced, i.e., an adjustment for co-existence is indicated (a random arrangement of sizes is rejected at $p<0.1$). These three taxa could thus be avoiding resource competition when sympatric. Maybe each particular *Rhizoglyphus echinopus* occurrence could be related to the chance opportunities for colonization choosing different size forms in their founder? The deutonymphs of these (and some other *Rhizoglyphus* species) are morphologically indistinguishable (OConnor *pers. comm.*), so different founder sizes however may be unlikely. Either way there is some evidence that perhaps *Tyrophagus nieswanderi* (T6) could theoretically coexist with them without strong competition. Of course they are usually spatially distinct on plant material, *Tyrophagus nieswanderi* on green tissues like leaves and *Rhizoglyphus* spp. infesting bulbs, so the Hutchison ratio may be irrelevant. These results are unsurprising as in fact, Simberloff and Boecklen (1981) effectively demolishes the idea of a “1.3 rule” at all.

If the extremes of congeneric species are examined, i.e., *ignoring* sympatry, an illuminating picture is seen in body size ratios (Table 4). Using the stated genera, breeding groups and samples in Table 1, the ratio of the idiosomal index for the largest species to the smallest *frequently* agrees with Hutchinson (1959). However, this size change infers approximately a doubling in effective body weight (i.e., $1.4^2 \approx 2$) suggesting that the size ratio might not just be about possible competition but also have something to do with how acarologists perceive differences in series of astigmatids to then pursue taxonomically? Only if taxonomic practice was actually steered by habitat sympatry would this be expected. Caution in the interpretation of astigmatid figures like those in Table 3 is strongly advised.

Unlike in Lepidoptera (Loder et al. 1998), there was no evidence, using the total count of different habitats which Hughes (1976) outlines, that larger mites are more generalists (see Wiens and Rotenberry 1980). Indeed the three species called as “Generalist” by Hughes (1976) herself (*Acarus siro* (A10b), *Acarus siro* [SW sp.] (A15), *Glycyphagus domesticus* (G5)) are in the mid-range of idiosomal index values. There was also no clear link between genus and ‘biome’ (*results not shown*). Of course, the term “generalist” here is relative as *Acarus siro* is truly found in more different types of such materials than other species. OConnor *pers. comm.* points out that Hughes (1976) was focused specifically on stored food and houses. *Acarus* species appear to have originated in the nests of vertebrates (most species are found only there, with deutonymphs phoretic on fleas), so one does not normally find *Acarus* species outside of nests or human associations. *Glycyphagus domesticus* is truly exceptional in that unlike most *Glycyphagus* species, which are restricted to nest habitats, this taxon can be found almost anywhere because its deutonymphs are wind dispersed. In fact it is *Tyrophagus* species, particularly *Tyrophagus putrescentiae*, that are considered as the most generalised of all Astigmata (OConnor *pers. comm.*). However, in a trophic design sense, this is not supported by this review, *Lardoglyphus* spp. being the closest to the ‘typical’ generalist mite (Figs. 16, 19) herein this study.

## Discussion

### General points

All studies have limitations and this review is no different. Many acarological textbooks (Evans et al. 1961; Krantz 1978; Krantz and Walter 2008) paint a general picture of astigmatids associated with stored products, and nests or dwelling-spaces of invertebrates and vertebrates. However, even for the common pest *Acarus siro*, what they actually feed upon has been disputed since the start of the 20th century; Munro (1966). As already pointed out, what a mite ingests and what it chews on is not necessarily the same as what it really digests and assimilates. Newstead states this pest astigmatid species attacks whole grain (Newstead and Duvall 1918a, b), but Solomon (1962) says *Acarus siro* only attacks broken or damaged grains eating out the (protein-rich) embryo. Recall that all Hughes (1976) is, is an attempt at an overview of broad predilections for a limited subset of Astigmata that can be found in stored food and houses. The true ecological range of the group (even excluding the fully parasitic taxa) is much broader, encompassing a wide variety of patchy or ephemeral habitats (OConnor *pers. comm.*). There are also many other reports of astigmatids eating other very different specific animal and plant foods, such as, nematodes (Bilgrami and Tahseen 1992, Walter et al. 1986), insect eggs (Brust and House 1988), fungi (Duek et al. 2001), plants (Evans et al. 1961; Fan and Zhang 2003), skin scales (Evans 1992), etc. This review does not cover these records in detail as its aim is understand astigmatid design first. However, those mites deemed generalist from the Hughes (1976) treatise generally *do* have a trophic design more like that of the simulated ‘typical’ mite species (Fig. 19), validating the algorithmic premise of this study in comparing individual species to a ‘central’ instance. The experimental rationale is sound.

Different statistical assumptions could have been used for estimates and statistical hypothesis tests (e.g., the Weibull distribution; Belu and Koracin 2013, rather than effectively a Normal distribution for distances from the reference typical mite location in ordinations). One obvious limitation of this review is that it assumes typicality of all of the mite samples used for each species (this might not be true, particularly as many had been laboratory cultured for some time). Poikilotherm phenotypes are plastic (Bouton et al. 2002), so future work could cover more examples of different populations of each species from different locations to confirm measurements. However, the actual specimens used in this review were certainly a reasonable sample of each of the astigmatid species, as for instance, the measured average ( ± SD) idiosomal index values for *Tyrophagus savasi* (T11) and *Tyrophagus robertsonae* (T87) broadly agree with those of their original allo- and paratype females given by Lynch (1989) (*Tyrophagus savasi*: T11 221.41 ± 11.33, T12 244.06 ± 22.05, T19 231.98 ± 16.17, T59 238.18 ± 13.46, CN3/1/1 226.46 ± 15.31, T92 249.12 ± 17.88, and *Tyrophagus robertsonae*: T87 201.84 ± 12.57, T24 182.31 ± 9.32). Culturing therefore may have only a small impact on species comparisons.

By using single population samples, no allowance for any character displacement or ecological release (Losos and De Queiroz 1997) that might occur between cohabiting species in the field has been allowed for either. This may not matter as, at least in oribatids (Gan et al. 2014), there is little evidence that physical environmental change fundamentally alters trophic structuring in communities. The species reviewed herein arose mainly from mono-cultures, so one cannot be completely sure that they represent the same stable morphology from the wild. However, one assumes that astigmatids probably behave similarly to their close relatives the oribatids in this aspect. Similarly, in using all females there are no conclusions to be had regarding if shape changes at all during ontogeny so avoiding within-species competition between stadia (Vincent et al. 2004) or if inbreeding over the long culturing periods for some populations have induced a shape change (Trotta et al. 2011).

Perhaps a better assumption of the scaling of static force with muscle volume could be used based upon say actual measurements of chelal closure in other larger related arthropods like crustacea, or methods like Heethoff and Koerner (2007) used? Another limitation is the assumption that the pennate muscle only occupies half the cheliceral shaft height, which is fairly stringent (the calculation of *F*2*AV* slightly disfavouring the *F*1*P* approximation). Perdomo et al. (2012) assumes effectively the whole of the chelicera to be adductive muscle which is not true. Future work might examine assuming different proportions (e.g., $\frac{2}{3}, \frac{3}{4}, \frac{4}{5}, \frac{5}{6}$ etc.) of the shaft height being filled with adductor pennate muscle or more accurate modeling of the true cross-sectional area from say histological sections. Many improvements could be made.

### What might be concluded about interrelationships from the results?

Hughes (1976) was a comprehensive attempt at the time which has not yet been significantly up-dated. Notwithstanding its limitations, given it exists and can be used, a variety of significant trophic design (${\hat{D}}$) correlates were found in this review (Table 5, Figs. 15, 16, 17 and 18). Surprisingly this did not include ’storage’. However, the design of acarines found in storage tended to be that of a medium size mite which best matches aspects of species found in houses (Fig. 17) and those with a high velocity ratio (Fig. 6). This is congruent with the view of Solomon (1962) that *Acarus siro* may have begun as a nest dweller. as well as its commoness in birds nests as found by Woodroffe (1953). Perhaps *Acarus gracilis* and *Glycyphagus domesticus* reflects a bat ‘house’ origin? More sampling for astigmatid species in that habitat is needed.

It is acknowledged that the detailed food specialism conclusions in this review depend upon this classification provided by Hughes (1976). Alternatives (for *B*) could be proposed perhaps based upon fungal selectivity (e.g., Sinha 1968) but the morphological ordination would not change (just the overlays of known *B*, and any extra hypothesis tests needing to be done). More information is available for sure on some of the species studied herein (OConnor *pers. comm.*). For example, *Forcellinia galleriella* is most commonly associated with honey bee hives and *Kuzinia laevis* with *Bombus* spp. nests. The *Thyreophagus* species TH4 is indicated as having originated in “sea bindweed”. Many species in this genus are known to burrow in the stems of reeds, others in subcortical habitats. *Glycycometus* ($=$ *Austroglycyphagus*) species are most commonly encountered in bat roosts as well as house dust and occasionally bee nests. The cheliceral design of bat associated species really needs further investigation. However, a definitive review of all astigmatid species habitats was not the intention of this study. Rather, determining the distinction of free-living taxa from a notional average morphological form, over which all sorts of biological hypotheses could be laid now or into the future, was the aim.

Notwithstanding, this review shows that free-living astigmatid mites have a common ‘Bauplan’ (at different scales) plus some detailed adjustments to their mechanical functioning. The typical trophic design of a free-living, saprophagous astigmatid mite is of a modest size arthropod (idiosomal index around $216\,\mu \text {m}$) with a potential reach around 98 $\mu$m and a gape about $27\,\mu \text {m}$. Such mites carry chelicerae about twice (1.903) as as long as high with an average chelal velocity ratio of 0.478. Unlike mesostigmatid mites (Buryn and Brandl 1992), the chelicerae make up $<\frac{1}{2}$ of body size on average over all species (mean *CLI*/$IL = 0.466$, exceptions were: *Aleuroglyphus ovatus* (AL2), *Chortoglyphus arcuatus* (CH1), *Dermatophagoides pteronyssinus* (D3), *Dermatophagoides farinae* (D4), *Dermatophagoides microceras* (D5), *Glycycometus hugheseae* (G3), *Glycyphagus domesticus* (G5), *Neosuidasia* sp. (LA1), *Tyrophagus savasi* (T11), and *Tyroborus lini* (T66) being bigger). Given that visually the total body length would be $>2-3$ times the idiosomal index, this ratio is in fact actually smaller. Gnathosomas are thus relatively small in astigmatids. The overall average estimated adductive crunch force on statically biting food (927 $\text {micron}^2$) sits in the range of *Carpoglyphus lactis* (302, minimum) to that of *Neosuidasia* sp. (2615, maximum). Gross variation in trophic design shown by cheliceral and chelal differentiation matches taxonomic position (Fig. 16). Astigmatid velocity ratios are substantially smaller than those inferred for oribatids (Fig. 8b). Astigmatids fill a different discrete niche. Conclusions for each species from the heuristic modelling are presented in Tables 6 and 7.

Specialism in saprophagous astigmatid trophic design can be seen in the different directions moving radially away from the typical mite design in the ordination (Figs. 20 and 21). The Procrustes shape analysis shows that this is a fundamental shape change (Fig. 22). Moreover compared to their larger relatives, small astigmatid mites with feeble chelae have a condyle relatively further forward, and a dorsal chelal lyrifissure ($\equiv$ flexure sensor) almost right above the apex of the moveable digit where the adductive tendon is attached. This approximates the chelal head to cheliceral shaft flexure region seen in uropodoids (Bowman 2021) suggesting a degree of mechanical convergence in the trophic functioning and potential fixed digit (or chelal head) movement in such mites. However, even those astigmatids with chelae designed with high velocity ratios only mildly overlap with the same measures in oribatids (Fig. 8b). They sit in a slightly different and fairly unique space, furthermore pyroglyphids are different to acarids.

It some ways this is all a surprise. Many lines of evidence strongly support the derivation of the Astigmata from within the oribatid lineage (OConnor *pers. comm.*). However, from the outset, the Astigmata appear to be specialists on patchy/ephemeral habitats, with dispersal between these accomplished by the phoretic deutonymph which is unlike oribatids. This matches possible necrophagy (or coprophagy?) as core habits. In this review, there is some possible commonality with minute microphytophagous oribatids like *Heterobelba stellifera* but, despite the often postulated common heritage of astigmatids and oribatids (Dabert et al. 2010, Domes et al. 2007, Maraun et al. 2004), the chelae of most saprophagous astigmatids as a general group cannot be definitively classed as exactly designed like macro- or panphytophagous oribatids in absolute measurements. This not surprising, the food sources and feeding morphology of these astigmatid mites must have diverged significantly from their non-specialised ancestors inhabiting the soil.

Changes in relative humidity can affect the softness/hardness of food. The outstanding feature of astigmatid pests of stored food is that they are intolerant of dry conditions and require a high relative humidity both in their food and their surroundings (Munro 1966). This suggests that say compared to free-living oribatids they must have relatively softer foodstuff to consume. This may go some way to explain why the velocity ratio of these free-living astigmatids does not match those of soil oribatids (Fig. 8b) who need to perhaps produce much higher adductive chelal forces in their phytophagy over a wider variety of environmental conditions. Could perhaps astigmatids fit any trends yet to be discovered amongst microphytophagous and the smaller macro-phytophagous oribatids? Remeasuring of such oribatids first studied by Schuster (1956) and Kaneko (1988) is needed to fill in missing key morphological measures (e.g., *CHI*, *VR*, etc.) for various species in order to calculate all the same summaries (and perform a wider geometric morphometric analysis) as in this review. Whether there are allometric homologues (i.e., log-log similarity between astigmatids and such oribatids) awaits further phylogenetic work.

### Is astigmatid taxonomic position relevant to trophic design (${\hat{D}}$)?

The distinction in trophic design between G6 (was *Glycyphagus destructor* = *Lepidoglyphus destructor*) and G5 (*Glycyphagus domesticus*) matches their different habits. *Glycyphagus destructor* is found frequently in association with *Acarus siro* consuming stored food products (Zárdková 1967; Cusack et al. 1975), but *Glycyphagus domesticus* feeds mainly if not-exclusively on micro-organisms (Evans et al. 1961), especially infesting inside furniture where it feeds on moulds growing on highly hygroscopic vegetable fibres (Hora 1934; Munro 1966). This trophic design distinction also supports the taxonomic assignment of G6 to *Lepidoglyphus* rather than *Glycyphagus*. Phylogenetically, *Lepidoglyphus* represents a monophyletic lineage within a larger *Glycyphagus* (OConnor *pers. comm.*). Removal of *Lepidoglyphus* renders *Glycyphagus* paraphyletic. No doubt this lineage specialized on something different than its ancestors, although many species still live in vertebrate nests. More research is needed.

Table 6 arranges the trophic designs of each astigmatid within their super-family, family and sub-family using the verbal classification of Bowman (2021). The surprisingly overall clear taxonomic correlate of trophic designs in this study (e.g., pyroglyphid specialisation, etc.) is like that for mesostigmatids (Buryn and Brandl 1992 in general, and Adar et al. 2012 Fig 6 for phytoseiids), as well as for oribatid families having similar feeding habits (Kaneko 1988). However, despite its clarity, this result may not be real. It could, of course, reflect a bias in the subjective formation of mite classifications by past workers (as for all biological classifications through history!). That is, this could be driven for instance by taxonomists inadvertently choosing or defining genera or family groupings on biological habit rather than shared phylogenetic origin. Alternatively, this pattern could be due to the poor coverage of the range of possible trophic types within each nominal taxon (family, sub-family, genus) and a lack of knowledge of their foodstuffs. Or perhaps, it could be that acarine phylogenetics has been strongly determined by trophic opportunities and vice-versa in astigmatids ? Certainly evolution produces concerted changes, biological systems morph as a whole over time not just individual characters only alter. More work is needed.

Modern classifications are phylogenetically based. New results from molecular phylogenetics, particularly the detailed works of Pavel Klimov (see http://insects.ummz.lsa.umich.edu/ACARI/staff/pklimov/), suggest that some of the astigmatid families diagnosed as monophyletic on the basis of morphological characters, do not hold up when sequence data is analyzed. The Glycyphagoidea as originally constituted by OConnor, appears to be quite polyphyletic, and the Acaridae to be diphyletic (but not dividing into the traditional Acarinae/Rhizoglyphinae; OConnor *pers. comm.*). This is still an evolving field full of exciting developments.

It would appear that speciation in the Astigmata has not been recent, as if it had there would have been insufficient time to allow such significant morphological divergence as found in this review. If DNA (or RNA) sequencing confirms their common phylogenetic history, then evolutionary trophic specialism in astigmatid mites would have to be ancient. Perhaps Next Generation Sequencing (NGS) can estimate when the breeding groups within *Tyrophagus putrescentiae* and *Tyrophagus similis* might have diverged in size and cheliceral/chelal shape? Indeed, would these breeding groups actually be sustained as evolutionarily distinct? When Donald Griffiths studied the “breeding groups” in the 1970s–1980s within what he considered species of *Tyrophagus*, there was little if any knowledge of the role that microorganisms (e.g., *Wolbachia*, *Cardinium*) can play in reproductive incompatibility. His results showing such incompatibilities between morphologically similar populations being necessarily evolutionary must be taken with a grain of salt (OConnor *pers. comm.*). Indeed the detection of acarine cryptic species appears to be correlated with the effort made to find them (Skoracka et al. 2015). Future work may tell if this trophic design pattern generally agreeing with nominal taxonomic groupings in Table 6 persists on examining more examples of different astigmatid species that have been confirmed molecularly.

### How do astigmatid designs (${\hat{D}}$) relate to what is known of other mites?

This review finds that most free-living astigmatid species fall into one of two main blocks (Table 8, Fig. 23):

1. Large surface living omnivores (A10b, C3, G6, KL, R1, R2, T38, T62, T66, TH4), or
2. Small burrowing/interstitial usually fragmentary feeders (AC204, A17, A4, C5, Ca4, CV1(66), D3, D5, F1, L1, S5, T6, T9, T11, T13, T32, T34, T44, T87, T90),

versus a remaining species group of A1, A15, AL2, C10, D4, CH1, G3, G5, L3, LA1, T40, T8, T17, T21, T7, T89, TH3. The former group (1) contains the herbivores and pest species, the latter group (2) may be obligate fungivores/microbial gleaners (although *Tyrophagus nieswanderi* is known to attack the foliage of cucumber plants; Johnston and Bruce 1965). The importance of this split is confirmed by the statistical tests in Table 8. The third group contains the remaining species of particular specialist designs (i.e., the off-diagonal blocks in Figs. 9, 10, 11, 12, 13 and 14). Eleven are either: small omnivores, a durophage (*Dermatophagoides farinae* D4), or a surface scraping/gleaning fragmentary feeding sub-type. The remaining six divide into groups of one to two species.

**Table 8:** Hypothesis tests for mite design groups see Fig. 23

Comparison	Distance *p* †	Angle *p* ‡	Angle *p* ‡ (random–isation)
Omnivore: Fragmentary feeder *	0.93	$< 0.001$	0.0001
Surface: Interstitial *	0.28	$< 0.001$	0.0001
Surface omnivore: Interstitial omnivore *	0.65	$0.001< p < 0.01$	0.0006
Surface fragmentary: Interstitial fragmentary *	0.039	$0.05< p < 0.10$	0.061

^†^Welch Two Sample *t*-test (alternative hypothesis: true difference in means is not equal to 0)

^‡^Watson’s Two-Sample Test of Homogeneity. *Result of interest. Omnivore: A10B, A15, AL2, C3, CH1, D4, G3, G5, G6, KL, L3, LA1, R1, R2, T38, T40, T62, T66, T7, T8, TH4. Fragmentary feeder: A1, A17, A4, AC204, C10, C5, CA4, CV1(66), D3, D5, F1, L1, S5, T11, T13, T17, T21, T32, T34, T44, T6, T87, T89, T9, T90, TH3. Surface: A1, A10B, C10, C3, G6, KL, L3, R1, R2, T17, T21, T38, T40, T62, T66, T7, T8, T89, TH3, TH4. Interstitial: A15, A17, A4, AC204, AL2, C5, CA4, CH1, CV1(66), D3, D4, D5, F1, G3, G5, L1, LA1, S5, T11, T13, T32, T34, T44, T6, T87, T9, T90. See Table 1

**Table 9:** Oribatid data used in Figs. 26, 27 and 28 sorted by genus and species Perdomo et al. (2012)

Taxon	*LEVERAGE* or Velocity ratio (*VR*)	$PHI^2$ (=$CHI^2$ herein)	Product ($\approx F2AV$)	Gape (*MDL* or *MOVLENGTH*)	Reach (*CLI* or *FIXLENGTH*)	Designation	Source
*Achipteria coleoptrata*	0.369	798.06	469.45	21.25	76.50	Primary decomposer	Perdomo et al. (2012)
*Amerus troisii*	0.713	3721.00	2653.23	46.00	181.00	Microphytophage	Schuster (1956)
*Amerus troisii*	0.299	676.00	338.00	22.00	87.00	Carnivore/Omnivore	Perdomo et al. (2012)
*Arcoplophora villosa*	0.500	1225.00	612.50	25.00	82.50	Fragmentary feeder	Kaneko (1988)
*Austrachipteria* sp.1	0.404	1764.00	1177.53	30.80	104.00	Lichenivourous	Perdomo et al. (2012)
*Austrachipteria* sp.2	0.433	1936.00	1201.39	31.63	101.50	Secondary decomposer	Perdomo et al. (2012)
*Belba verticillipes*	0.571	4761.00	2716.57	51.00	176.00	Microphytophage	Schuster (1956)
*Ceratoppia sexpilosa*	0.544	4761.00	2588.79	48.00	169.00	Microphytophage	Schuster (1956)
Ceratozetoidid	0.376	858.49	632.57	19.00	78.00	Secondary decomposer	Perdomo et al. (2012)
*Chamobates borealis*	0.420	1667.36	1013.08	26.33	97.33	Secondary decomposer	Perdomo et al. (2012)
*Chamobates cuspidatus*	0.396	3025.00	1932.64	36.00	139.00	Secondary decomposer	Perdomo et al. (2012)
*Chamobates voigtsi*	0.453	1849.00	1280.08	26.00	95.00	Secondary decomposer	Perdomo et al. (2012)
*Crotonia* sp.	0.405	1778.31	1042.27	35.83	104.25	Secondary decomposer	Perdomo et al. (2012)
*Cultroribatula* sp.	0.443	1777.47	1235.19	29.50	95.20	Primary decomposer	Perdomo et al. (2012)
*Eohypochtonius magnus*	0.231	625.00	144.23	32.50	95.00	Fragmentary feeder	Kaneko (1988)
*Epilohmannoides esculatus*	0.667	3025.00	2016.67	52.50	125.00	Macrophytophage	Kaneko (1988)
*Euepicrius lootsi*	0.305	1764.00	696.14	65.25	137.75	Carnivore/Omnivore	Perdomo et al. (2012)
*Eupelops* sp.	0.375	2500.00	937.50	20.00	232.50	Microphytophage	Kaneko (1988)
*Gamasellus *sp.	0.288	882.09	254.64	53.00	103.00	Carnivore/Omnivore	Perdomo et al. (2012)
*Gymnodamaeus bicostatus*	0.693	3600.00	2493.66	41.00	136.00	Microphytophage	Schuster (1956)
*Hermaniella granulata*	0.706	5184.00	3659.29	51.00	175.00	Macrophytophage	Schuster (1956)
*Heterobelba stellifera*	0.444	756.25	336.11	22.50	87.50	Microphytophage	Kaneko (1988)
*Hypochthonius rufulus*	0.268	361.00	152.00	19.00	71.00	Carnivore/Omnivore	Perdomo et al. (2012)
*Hypodamaeus riparius*	0.388	1444.00	748.74	27.00	98.00	Secondary decomposer	Perdomo et al. (2012)
*Lanceoppia* sp.	0.284	729.00	364.50	24.00	95.00	Carnivore/Omnivore	Perdomo et al. (2012)
*Liacarus acutidens*	0.757	22500.00	17027.03	92.50	300.00	Microphytophage	Kaneko (1988)
*Malaconothrus talaitae*	0.449	462.25	313.27	18.44	47.83	Primary decomposer	Perdomo et al. (2012)
*Nothrus palustris*	0.479	1156.00	770.67	24.00	71.00	Primary decomposer	Perdomo et al. (2012)
*Nothrus silvestris*	0.568	5625.00	3196.43	63.00	170.00	Non-specialised	Schuster (1956)
*Oppiella nova*	0.429	400.00	171.43	17.50	55.00	Microphytophage	Kaneko (1988)
*Oribatula tibialis*	0.429	729.00	592.31	16.00	63.00	Primary decomposer	Perdomo et al. (2012)
*Paradamaeus clavipes*	0.407	3481.00	1779.18	45.00	145.00	Secondary decomposer	Perdomo et al. (2012)
*Phthiracarus* sp.	0.477	3844.00	2506.96	46.00	130.00	Primary decomposer	Perdomo et al. (2012)
*Platynothrus peltifer*	0.520	1521.00	1244.45	22.00	75.00	Primary decomposer	Perdomo et al. (2012)
*Protoribates lophotricus*	0.667	6006.25	4004.17	52.50	157.50	Panphytophage	Kaneko (1988)
*Pseudoceratoppia* sp.	0.374	1296.00	704.62	25.75	96.25	Secondary decomposer	Perdomo et al. (2012)
*Rhysotritia ardua*	0.750	7225.00	5418.75	60.00	182.50	Macrophytophage	Kaneko (1988)
*Rhysotritia ardua*	0.750	7225.00	5418.75	60.00	182.50	Panphytophage	Kaneko (1988)
*Steganacarus cf. clavigera*	0.663	4900.00	3248.52	54.00	140.00	Macrophytophage	Schuster (1956)
*Tectocepheus velatus*	0.432	256.00	186.18	11.00	37.00	Primary decomposer	Perdomo et al. (2012)
*Xenillus tegeocranus*	0.741	6561.00	4860.00	54.00	202.00	Non-specialised	Schuster (1956)

†Mesostigmatid. LEVERAGE, PHI $=$ FIXHEIGHT, MOVLENGTH, and, FIXLENGTH from Perdomo et al. (2012)

**Table 10:**
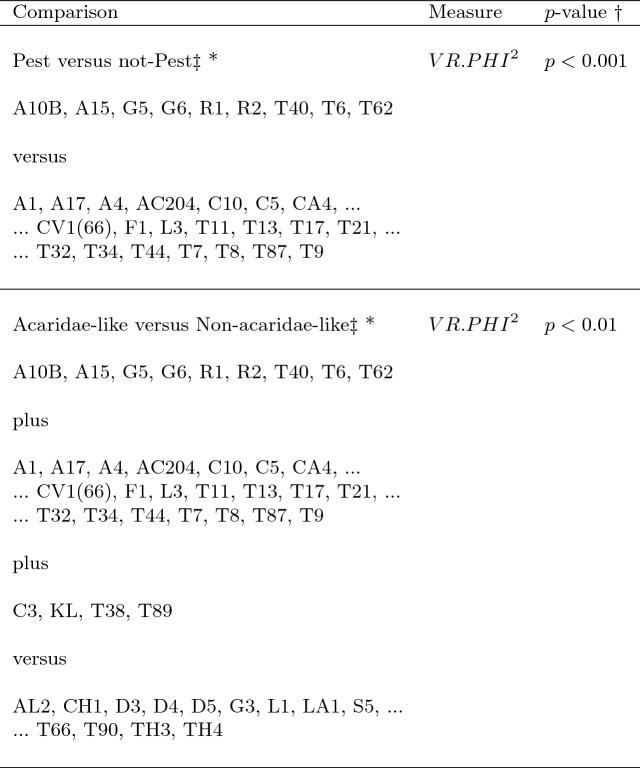
Statistical tests of astigmatid cohorts with respect to food toughness (see Fig. 27)

Species codes as in Table 1. †$=$ Welch Two Sample *t*-test (alternative hypothesis: true difference in means is not equal to 0). *Result of interest. Population codes as in Table 1. ‡ Non-pest species are mainly ‘fragmentary carnivores’. Non-acaridae-like includes all the other families but also the large acarids like *Aleuroglyphus ovatus*, the two *Thyreophagus* spp., *Tyroborus lini* (better considered perhaps as a pest?) and *Tyrophagus tropicus* and is based upon a threshold of velocity ratio (or leverage) $>0.5$

**Table 11:**
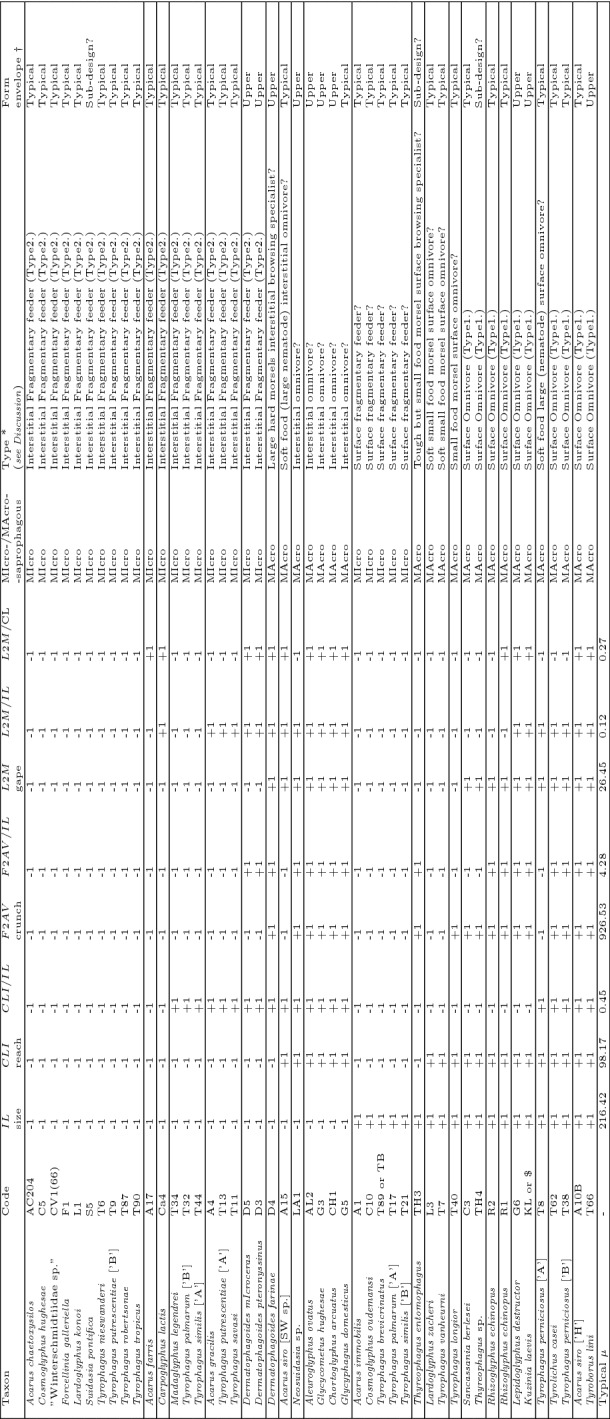
High-throughput screening field-use look-up table

Astigmatids ordered by coded heuristic ($+1 =$ above typical reference mite average, $-1 =$ below) left-to-right then by Species. *Type 2. = obligate fungi-microbivore? † see Fig. 24. Horizontal lines indicate groupings

This distinction between the two main species groups:

1. *Acarus siro*, *Sancassania berlesei*, *Lepidoglyphus destructor*, *Kuzinia laevis*, *Rhizoglyphus echinopus* (both samples), *Tyrophagus perniciosus* [‘B’], *Tyrolichus casei*, *Tyroborus lini*, and *Thyreophagus* sp.
2. *Acarus chaetoxysilos*, *Acarus farris*, *Acarus farris*, *Cosmoglyphus hugheseae*, *Carpoglyphus lactis*, “Winterschmidtiidae sp.”, *Dermatophagoides pteronyssinus*, *Dermatophagoides microceras*, *Forcellinia galleriella*, *Lardoglyphus konoi*, *Suidasia pontifica*, *Tyrophagus nieswanderi*, *Tyrophagus putrescentiae* [‘B’], *Tyrophagus savasi*, *Tyrophagus putrescentiae* [‘A’], *Tyrophagus palmarum* [‘B’], *Madaglyphus legendrei*, *Tyrophagus similis* [‘A’], *Tyrophagus robertsonae*, and *Tyrophagus tropicus*

matches the philosophy (but does not exactly the calculation rule used by previous researchers) for that of macrophytophagous versus microphytophagous oribatids. Note, that the average $\frac{fd}{md}$ measure used in oribatids studies for the astigmatid group (1) is 3.65, which is smaller than the average $\frac{fd}{md}$ measure for the second astigmatid group (2) at 3.84 (and the equivalent value for the remaining species group is 3.71 congruently sitting in the middle). The former reference macrophytophagous oribatid group feeds mainly on fragments of higher plant material and rarely on fungi, the latter reference oribatid group consumes a wide range of microflora such as fungi, bacteria, yeasts (Wallwork 1983) and algae (see references in Walter 1987). Given that astigmatids tend to feed on decaying material these two astigmatid index classes (1. v 2.) might be more precisely denoted as macrohumiphagous (aka macrosaprophagous) and microhumiphagous (aka microaprophagous); see Tarman (1968). If they feed on animal derived material, labelling them as macronecrophages versus micronecrophages could be justified. Taking this ratio approach further, the broad cut of astigmatid species in terms of their gross functionality is summarised in Fig. 24 derived from Fig. 6 (compare this to the change in shape of their chelicera; (Fig. 22a), this pulls out most of the specialist mites (i.e., not in 1. and 2. above).

The fact that *Rhizoglyphus echinopus* both samples R1 and R2 being in the first group (1.) fits with the feeding observations of Hodson (1948). The widely distributed *Dermatophagoides pteronyssinus* (represented here by D3) is particularly present in the humid coastal climates of Western Europe and North America. *Dermatophagoides farinae* (here represented by D4) prefers drier continental climates of Central Europe and Central USA (Evans 1992). Could this explain their difference in feeding design herein? D4 being like a durophage (hard, perhaps drier food?). Could dust consumption be considered as necrophagy?

As in birds (Wiens and Rotenberry 1980), the clear patterns in astigmatid morphology with diet is preferentially driven by the larger species. Any proposed pan-saprophagy (i.e., non-specialised feeding, see Table 7) amongst the first group (1.) can only be determined by very close examination of their gut contents (e.g., Walter 1987). Within the microsaprophagous group, there may be some feeding specificity (Hartenstein 1962; Luxton 1972). If the latter group (2.) were zoophagous and ate nematodes (see references in Walter 1987, and references for acarids in Muraoka and Ishibashi 1976) then they *per force* would need to be consumers of small worms. *Sancassania berlesei* can be reared in the laboratory on nematodes (El-Atta et al. 2014) but is also capable of feeding on a wide range of substrates as long as the humidity is high (grain, manure, dead insects, fungi, nematodes; OConnor *pers. comm.*). This genus is mainly associated with scarabaeoid beetles, where they may feed on provisioned larval food or on dead insects themselves. If *Sancassania berlesei* (C3) were nematophagous in the field (like other *Sancassania* sp.; Karagoz et al. 2007; Sell 1988) it could probably deal with quite large worms. *Tyrophagus putrescentiae* (T9 [‘B’], T13 [‘A’]) being classed as a microsaprophage matches its known complicated feeding relationship with nematodes, fungi and near symbiotic bacteria (Brust and House 1988; Bilgrami and Tahseen 1992; Evans et al. 1961; El-Atta and Osman 2016; Smrž et al. 2016 and many other references therein). *Tyrophagus similis* is known to eat nematodes, fungi and algae; Walter (1987). The two breeding groups may perhaps specialise in different size food (Table 6).

Turning to other mites, Perdomo et al. (2012) investigated various oribatids with stable radioisotopes. They related the equivalent of adductive force *F*1 in this review (i.e., cross-sectional area of cheliceral muscle as estimated by cheliceral height squared, their $PHI^2=CHI^2$) to the mite’s trophic role. They state:

...Leverage and estimated cross-sectional area of the levator can be used in conjunction to differentiate between guilds with higher accuracy than each measure would provide independently (*their* Fig. 4). Carnivorous / omnivorous / scavenger mites have chelicerae that have a low leverage index and little space for levator muscles. Primary decomposers generally have chelicerae with a high leverage index, and an estimated cross-sectional area smaller than $2000\,\mu m^2$. The pthiracarid mite is an exception to this general pattern, with its chelicerae showing the second largest area of all species. Chelicerae of secondary decomposers (feeding mostly on fungi) generally have a large cross-sectional area (higher than $2000\,\mu \text {m}^2$) and a leverage index between 0.5 and 0.7...”.

They also claimed that leverage was independent of body size (as also is the case for the velocity ratio (*VR*) versus idiosomal index (*IL*) for the astigmatids herein $R^{2}=0.0317$, despite Figs. 5 and 6a). There was also a mild correlation of oribatid cheliceral cross-sectional area with log(overall oribatid body size) ($R^{2}=0.3752$). For the astigmatids in this review this was also mildly supported for *F*1*AV* versus *IL* ($R^{2} = 0.2215$). However, it is not so much how hard a mite can pull on its adductive tendon inside the chelicera as to how strong a force it can deliver in-between its chelal tips. Nature sees the equivalent of $F2AV=F1AV*VR$ i.e., $Leverage*PHI^{2}$ (see argument in the “Expected results” section above). In particular if this food crushing force is especially high for that physical size of the mite concerned. This is explored further below in the context of zoophagy.

In astigmatids, adductive chelal crunch force (*F*2*AV*) is mildly correlated with aspect ratio ($R^2=0.377$). Where oribatids would sit in the plot in Fig. 24c is not clear. Future work could get better estimates of idiosomal index in oribatids (rather than total body size from SEMs used by Perdomo et al. 2012) in order to decide if some of the astigmatids could ‘pack a punch’ similar to the relative chelal crunch-force values of oribatids. Could oribatids, by their body armouring, be in anyway constrained in their possible cheliceral height and have they opted for a slightly different shape of chelae to deal with hard food? Is this the origin of any distinction between the sub-orders, or is it just their overall body size versus that of astigmatids? A comparative geometric morphometric study should be able to confirm or refute this.

### Possible validations

Some external validation can be made of the two main astigmatid body plan types 1. and 2. above. A classification of feeding guilds in oribatids has been proposed by Siepel and Ruiter-Dijkmann (1993) based upon the presence and absence of saccharolytic enzymes (like that for isopods and millipedes; Beck and Friebe 1981). Macrophytophagous oribatids generally possess cellulase in their digestive enzyme complement, whereas micro-phytophages do not (Zinkler 1972; Wallwork 1983). Could it be the same situation for the assignation of the two main astigmatid morphological forms (1. and 2.) above?

Four out of five of the astigmatids previously recorded or testing positive for cellulase in Bowman and Childs (1982) that were also examined in this morphological review (i.e., *Rhizoglyphus echinopus* R1 and R2 tested, *Tyrophagus longior* T40 tested, *Aleuroglyphus ovatus* recorded represented here by AL2, *Sancassania (Caloglyphus) berlesei* recorded represented here by C3) are concluded to belong in the omnivore ‘macrosaprophagous’ class. *Sancassania (Caloglyphus) berlesei* has been classified as a phytophage (although necrophagy on dead soil insects is recorded; Krantz 1978). *Rhizoglyphus* spp. damage healthy bulbs of ornamentals (Michael 1903; Hodson 1948; Hussey et al. 1969). Only one species which is recorded as having cellulolytic activity (i.e., *Thyreophagus entomophagus* represented here by TH3) is assigned to the fragmentary feeding ‘micro-saprophagous’ class above. Furthermore all three species recorded as lacking cellulase that were also examined in this morphological study (*Carpoglyphus lactis* represented here by Ca4, *Tyrophagus similis* represented here by T21 [’B’] and T44 [‘A’], *Tyrophagus putrescentiae* represented here by T9 [‘B’] and T13 [‘A’]) are concluded to belong to the ‘microsaprophagous’/fragmentary feeding class. This is despite *Tyrophagus similis* being a crop pest; Krantz and Lindquist (1979). Cellulase patterns are thus congruent with this review’s morphological design conclusions.

This is very strong independent corroborative evidence that the discovered trophic designs (1. and 2.) do indeed match likely life habits. Sinha and Mills (1968) consiliently confirm *Tyrophagus putrescentiae* to be a more efficient fungivore than *Acarus siro*). Note that *Acarus siro* (represented here by A10b and A15) is allocated to the macrosaprophagous grouping but so far has only inconclusive cellulase results, this needs rechecking. Unfortunately Bowman and Childs (1982) did not test any species represented here by the above proposed fragmentary feeding (microsaprophagous) class for their levels of fungal digesting enzymes (such as trehalase and chitinase), so the converse logic check cannot be made. Follow-up work could perhaps discern xylophages and phyllophages amongst the macrosaprophagous astigmatids, and delineate mycophages, bacteriophages (Smrž et al. 2016) and phycophages amongst the microsaprophagous astigmatids?

Amongst the remaining species, *Aleuroglyphus ovatus* (AL2), *Neosuidasia* sp. (LA1), *Chortoglyphus arcuatus* (CH1), *Glycycometus hugheseae* (G3) and *Glycyphagus domesticus* (G5) appear to be variant trophic designs of the standard omnivore with a particular small body size and thus perhaps have an interstitial habit. They (with *Dermatophagoides farinae* (D4); see below) essentially cover the remaining design space (Fig. 23g) not covered by the large omnivore and small fragmentary feeder design; see ‘upper convex hull’ in Fig. 25c. Of the remaining free-living astigmatids (not listed above) these are scattered across the design space within the one other lower ‘convex hull’ (Fig. 25c) in small particular areas. From Tables 6 and 7, *Tyrophagus perniciosus* [‘A’] (T8) together with its small body size (= a burrowing/interstitial adaptation?) design variant *Acarus siro* [SW sp.] (A15), both appear to be soft food specialisms of an omnivore ‘Bauplan’—they thus may be trophically designed to eat large nematodes? The *Tyrophagus longior* (T40) design is a specialism of a surface omnivore design for small food morsels (*Tyrophagus longior* is found on hay and straw as well as grain; Munro 1966 and will attack leaves of plants; references in Krantz and Lindquist 1979). *Lardoglyphus zacheri* (L3) and *Tyrophagus vanheuri* (T7) appear to be a soft small food morsel design specialism of the *Tyrophagus longior* (T40) surface omnivore variant. *Thyreophagus entomophagus* (TH3) is trophically designed as a tough but small food morsel surface browsing specialist. Does this match its field records? Could this be evidence of pollenophagy? *Dermatophagoides farinae* (D4) is trophically adapted for interstitially browsing large hard morsels of food. *Acarus immobilis* (A1), *Cosmoglyphus oudemansi* (C10), *Tyrophagus brevicrinatus* (T89), *Tyrophagus palmarum* [‘A’] (T17), and *Tyrophagus similis* [‘B’] (T21) are large body size (= surface habit adapted?) variants of the fragmentary feeder (or obligate fungivore/microbiovore) design. That would partly fit with *Tyrophagus similis* eating minute holes in young cucumber leaves (see references in Krantz and Lindquist 1979). Of course small gape, soft food specialists could also be small nematode feeders. All of these observations suggest further work.

### Does possible zoophagy lead to useful insights?

Figure 26 plots the astigmatids of this review on the axes used by Perdomo et al. (2012) together with figures for mesostigmatids from Bowman (2021) and data extracted from various Figures and Tables in Schuster (1956) and Kaneko (1988). Convex hulls for the oribatid species radiologically denoted with a soil community trophic role by Perdomo et al. (2012) are added for comparison. A variety of insights can be made.

Firstly, from Fig. 26a:

- All astigmatids and oribatids fall into the ‘crushing action’ rather than ‘cutting action’ chelal design (see Bowman 2021).
- Some astigmatids fall into the ‘carnivores’ trophic community defined by Perdomo et al. (2012) where a host of low adductive force micro- and meso-cephalic mesostigmatids of ‘worm-like’ feeding habit also lie. Such mesostigmatids invariably had been given the vertebrate feeding analogy of a ‘Kitten, Mole, Shrew, Small pig’ in Bowman (2021). They may facultatively feed on small nematodes.
- Most astigmatids appear to fall between the ‘carnivores’ group and the convex hull of oribatid secondary decomposers. Only a few would be scored as like oribatid secondary decomposers: *Thyreophagus* sp. (TH4), *Thyreophagus entomophagus* (TH3), *Suidasia pontifica* (S5), and *Dermatophagoides pteronyssinus* (D3) of the ‘upper group’ in Fig. 24c plus *Lardoglyphus koni* (L1). Only one *Dermatophagoides microceras* (D5) is near the Lichenivore *Austrachiptera* sp. 1 and would be classed in the oribatid primary decomposer group. These all have *F*1*AV* equivalent values in the range of crushing style predators like *Pergamasus digitulus*, analogously described as a ‘Big cat’, and *Rhodacarellus epigynalis*, analogously described as a ‘Rat’ in Bowman (2021).
- A variety of astigmatids (most of those in the ‘upper’ distinct design group of Fig. 24c and many of the oribatids studied by other authors; especially by Schuster (1956), have estimated chela adductive tendon forces (*F*1) well above those of the oribatid mites studied by Perdomo et al. (2012). Perhaps the particular species studied by the latter authors were relatively small? However, given the relative left-right position of the radioisotope confirmed trophic community roles at $PHI^2$ values less than 4000, it is tempting to accept that the top right positioned extra oribatids are probably ‘primary decomposers’ in the soil community and the top middle positioning extra oribatids are probably ‘secondary decomposers’ in the soil community (i.e., one can extend the convex hull categorisations vertically). This needs confirmation by the extension of Perdomo et al. (2012)’s radio-isotope work and re-measurement of these larger species (only partial information is available in Schuster 1956’s and Kaneko 1988’s publications). For sure together with most from the ‘upper group’ designed Astigmata from Fig. 24c, i.e., *Aleuroglyphus ovatus* (AL2), *Dermatophagoides farinae* (D4), *Glycycometus hugheseae* (G3), *Lepidoglyphus destructor* (G6), *Kuzinia laevis* (KL), *Neosuidasia* sp. (LA1), and *Tyroborus lini* (T66), they all have adductive tendon primary force (*F*1) equivalent values to ‘crushing kill’ style mesostigmatids denoted as analogues to vertebrate boars, hyenas, and Tyrannosaurs in Bowman (2021). Many of the latter mesostigmatids are durophages attacking hard material including insect eggs.

Secondly, from Fig. 26b:

- Omnivorous astigmatids have higher adductive tendon forces than fragmentary feeders irrespective of whether being surface or interstitial habit designs (i.e., irrespective of overall body size). Interstitial astigmatids have more in common with oribatid secondary decomposers than surface feeding astigmatids do. *Dermatophagoides microceras* (D5) is particularly distinct in design.

Thirdly, from Fig. 26c:

- Panphytophagous oribatids, non-specialised and macrophytophagous species studied by Schuster (1956) and Kaneko (1988) appear to be more like primary decomposers. They have adductive tendon primary force (*F*1) equivalent values to ‘crushing kill’ style mesostigmatids denoted as analogues to vertebrate boars, hyenas, and Tyrannosaurs by Bowman (2021). Many of the latter predators are durophages attacking hard material including insect eggs. This validates the oribatid designation by Schuster (1956) and Kaneko (1988). These latter authors’ microphytophagous oribatids overlap with secondary decomposers from Perdomo et al. (2012). The ‘fragmentary feeder’ oribatid type used by Kaneko (1988) clearly matches the ‘carnivore’ role from Perdomo et al. (2012).

Fourthly, from Fig. 26d:

- Macrosaprophagous astigmatids have generally larger tendon (*F*1) adductive forces than microsaprophagous astigmatids but both are generally distinct from the oribatid trophic design.

Perdomo et al. (2012)’s approach is valid and useful, but as pointed out above, Nature sees the *resultant* of tendon adductive force (*F*1) and leverage when a jaw tries to break food up i.e., *F*2.

Figure 27 recalculates the adductive crunch force ( $\equiv$ *F*2*AV*) as a surrogate of the toughness of food material for all the mites, and plots this versus leverage or velocity ratio (as available across the studies) as a surrogate of speed of chelal closing (Table 9 gives the oribatid data). Boundaries are proposed for the understanding of various historical terminologies. The speed of closing at 0.5 is taken from the velocity ratio value of the cross-point in Fig. 24c. This marks a shift in design from essentially acarid astigmatids and very small size taxa (to the left) to something quite different (i.e., elongate/giant acarids, glycyphagids, pyroglyphids, etc., and oribatids to the right). Astigmatids on either side of the boundary certainly have very different (and statistically significant) abilities to handle the toughness of food (Table 10). It probably also indicates a boundary in facultative carnivory (cf. nematode feeding), below it is probably more and more predominantly fungal feeding taxa as crunch-force values fall. All (bar one) mesostigmatids have chelae with velocity ratios below this. This suggests that attacking live nematodes only occurs below this threshold irrespective of gape (which determines nematode prey size) or reach (which determines access into pores/crevices, etc.). The boundary at speed of closing = 0.65 is calculated to be the midpoint threshold between Perdomo et al. (2012)’s primary (to the right) and secondary decomposers (to the left). It is close to the 0.6 used morphologically by Kaneko (1988) between his phytophagous types. The boundary at ‘food toughness’ of just over 1000 is midway between the values of fragmentary and microphytophagous oribatids. The boundary at ‘food toughness’ just over 2500 is midway between the values of microphytophagous and macrophytophagous oribatids. The boundary just below 4000 in ‘food toughness’ is midway between those values of macrophytophagous oribatids and those scored as non-specialised or panphytophages by Schuster (1956). Only the latter three oribatid groups (i.e., $>4000$ on *y*-axis) are likely to contain primary decomposers. Secondary decomposer oribatids are a mixture of micro- and macrophytophages. For this latter reason, the use of this older morphologically derived terminology to delineate trophic roles in communities without considering adductive force (*F*2*AV*) is strongly questioned.

Where does this leave the detail of astigmatid designs and roles? In Fig. 27, are the astigmatids of ‘speed of closing’ $<0.5$ and ‘food toughness’ $>1000$ say, the pest species? The mites plotting here are: A4, A15, A10B, C3, T6, G5, G6, R1, R2, T38, T40, T62, i.e., *Acarus gracilis*, *Acarus siro* [SW sp.], *Acarus siro*, *Sancassania berlesei*, *Tyrophagus nieswanderi*, *Glycyphagus domesticus*, *Lepidoglyphus destructor*, *Rhizogplyphus echinopus* both samples, *Tyrophagus perniciosus* [‘B’], *Tyrophagus longior*, and *Tyrolichus casei*. *Sanacassania belesei* is a large nematode feeding necrophagous mite. Perhaps Luxton (1972) was right but zoophagy/coprophagy/necrophagy should be applied to astigmatids and not to oribatids? Maybe Böttger (1970) was right, there is a scavenging carrion feeder form that these astigmatid mites represent? Taking the common, recognised agricultural pest astigmatid species to be: *Acarus siro*, *Glycyphagus domesticus*, *Lepidoglyphus destructor*, *Rhizoglyphus echinopus*, *Tyrolichus casei*, *Tyrophagus longior*, and *Tyrophagus nieswanderi*, this conclusion that pest species are differentially designed like strong ‘shredders’ biting off chunks of material (i.e., the classification of Fashing 1998) versus those acarids scraping or gleaning soft fungal fragments is supported (Tables 5, 10). The debate in the Introduction has merit. This region is where the astigmatid acaroid design facilitates concentrating on highly nutritious often proteinaceous food i.e., $\equiv$ effective ‘carnivory’ but with the ‘prey’ not moving (or it being dead). In that way, ‘non-moving’ high-protein plant tissues could also be considered as ’dead animals’ and the astigmatid mites as ‘vegan carnivores’! Hughes (1959) was on the right track. This would be also the region where specialising in egg feeding (oophagy) might be indicated. Bowman (1981) did find high levels of protease in pest astigmatids. Other species that might be considered as pests because of their seeming ability to handle tough proteinaceous food are *Sancassania berlesei* (C3), *Kuzinia laevis* (KL), *Tyrophagus pernciosus* [‘B’] (T38) and *Tyrophagus brevicrinatus* (T89) (which plot above the assumed pests). There is of course the known intolerance of acarids to dry conditions, however, *Kuzinia laevis* (KL) is a particular outlier in this group with a colossal crunch force comparable to a large oribatid—perhaps it focuses on highly dried ‘necrophagous’ material? This species certainly consumes proteinaceous pollen. Is bee-hive pollen especially hard to crack? Could it be that this astigmatid is facultatively an arthropod egg eater as the closest mesostigmatid to it is *Glyphtholaspis confusa* thought to be a hyena-like durophage specialising in cracking insect eggs (Bowman 2021)? Does *Kuzinia laevis* have particular adaptations to deal with water stress to match?

So, to summarise. Saprohagous astigmatid ‘carnivores’ have promptly closing rather delicate cheliceral chelae suitable for chewing soft food material including microbial and fungal fragments (plus perhaps small nematodes, rotifers, etc.). Most astigmatid chelae close at a slight slower rate than this and are adapted for modestly tough food (e.g. larger nematodes, nutritiously rich proteinaceous deposits like stored foodstuffs, dead bodies, etc.). These two feeding styles comprise most of the acarid species reviewed herein. Only highly structurally-derived acarid, glycyphagid and pyroglyphid species specialise into oribatid-like roles. Fig. 28 confirms that acarids mainly vary amongst themselves simply by ‘shrinkings/swellings in size’ and crunch force. The relationship of crunch force generally scaling with chelal gape or with cheliceral length similarly for all types of mite design and for both pest and non-pest species (*regression lines committed for clarity*). Those soft-food feeders with a larger gape and reach could tackle larger more active nematodes. Small reach confines such astigmatids to be gleaners. Those small gape species must be obligate microsaprophages, with those of the smallest gape (“Winterschmidtiidae sp.”) being almost ‘planktonic collectors’ (cf. Fashing 1998). Pest species may excavate deeper and cause greater damage in human foodstuffs due to their generally larger gape and reach compared to the typical acarid fragmentary feeding fungivores. The fundamental trophic shape of: pyroglyphids is distinct (and is in common with *Lepidoglyphus destructor* G6), while that of *Glycyphagus domesticus* (G5) is more in common with the larger plant feeding acarids *Rhizoglyphus echinopus* (R1) and *Tyrophagus longior* (T40) (Fig. 22c).

### Relevance to other groups

As a sub-order, astigmatids trophically *are* designed like non-armoured oribatids. They are not designed like mesostigmatid soft food specialists. The key competency in these actinotrichid groups has been the ability to increase cheliceral height (with its input effort arm) and thus ‘pack a punch’ to deal with hard/durable material, unlike the situation in anactinotrichid predators where a prey-cutting action by chelae and cheliceral extension to augment reach (i.e., a different type of ‘gnathosomisation’) is also relevant. Given idiosomal index (*IL*) values for oribatids becoming available as reference dimensions (to other mite groups) then the impact of simple change in ‘bigness’ within this latter soil-inhabiting order could be plotted in Fig. 24c with appropriate bubble sizes for comparison. One expects the regression line to steepen for oribatids and comparatively to be swept anticlockwise around the origin as the velocity ratio increases and the aspect ratio diminishes (e.g. on *L*1*U* and *CHI* increasing). Absolute size would be factored out by the use of ratios but a boundary between an upper soil-inhabiting group capable of excessive chelal ‘punch’ e.g., panphytophages, etc., and a lower oribatid group, e.g., fragmentary feeders with an orthogonal boundary between the two at about velocity ratio values of 0.6–0.65 is expected. It is this gnathosomal height differentiation that marks the sarcoptiform adaptation for different lives. Could it be confirmed amongst the opilioacarids too who are also non-predatory arachnids?

All of these above subtypes within the main astigmatid ‘Bauplan’ distinction need field validation from radioisotope (Perdomo et al. 2012) or metagenomic tracking (and hopefully detailed observations of enzymic actions and feeding behaviour in the wild) in order to confirm this synthesis.

It would be interesting to see where the nidicolous *Tyrophagus dimidiatus* might sit within this scheme, this species is also found within the leaf sheaths of mite and insect damaged grasses, cereals and other plants (Evans et al. 1961). The name *Tyrophagus dimidiatus* has been considered as a junior synonym of *Tyrophagus putrescentiae* for a long time. What the actual species that was studied by Evans et al. (1961) is unclear (OConnor *pers. comm.*), *Tyrophagus putrescentiae* can be found in nests as well as many other habitats.

Furthermore what do sap feeding algophagids (Evans et al. 1961) like *Hericia* spp. look like and where would they plot in Figs. 24c and 27, and how much are they or are they not like *Carpoglyphus*? SEM figures in Fashing and Okabe (2006) and Fashing (2008) illustrate elongate chelicerae with very different areas along the moveable digit clearly designed for different functions. *Algophagus pennsylvanicus* is both a scraper (grazer) shearing fungal hyphae from the surface of decomposing leaves as well as probably a species filtering fine particulate organic matter from the water surface film (therefore being also a “collector”; Fashing and Campbell 1992). What design do other glycyphagid genera (*Grammolichus*, or *Sclerolichus*; Krantz 1971) exhibit? Much is left for other acarologists to pursue.

## Future work

Many possibilities for follow-up work arise. Given the cosmopolitan distribution of astigmatids any of these following suggestions ought to be straightforward investigations for a budding acarologist to undertake.

### Mastication surface

Looking at the actual chelal surface that masticates food could be interesting. The active chelal mastication surface can be defined as that part of the upper region of the moveable digit able to grasp food material in some way against the fixed digit. It begins at the digit tip and stops at the rise of the basal ‘coranoid’-like process on the moveable digit. This is essentially at a distance from the condyle along the *L*2*M* axis equivalent to the size of *L*1*U*. A variety of summaries of the toothed, pocketed and bladed mastication surface of the moveable (and fixed) digits of the astigmatid cheliceral chelae could be used depending upon assumptions as to how the digits are deformed in evolutionary development. That is, the chela surface might be: stretched vertically, flexed or bent up and down by local rotations, or suffer creep or shearing at any one point along it. These are convenient artifices, like thin-plate splines they are free of the actual cellular mechanism and differential growth changes carried out to achieve them.

For all the astigmatid species considered in this review it could therefore be worth looking at their specific dentition patterns and likely adductive force at each fixed digit and moveable digit tooth along their length in a research project much as started by Akimov and Gaichenko (1976). Is the velocity ratio of ‘back teeth’ important in predicting diet as it is for molar1 teeth in mammals (Grossnickle 2020)? What sort of bite forces can be delivered at different gapes as the chela closes? Is the depth of the moveable digit under any tooth or pocket related to resisting any bending from the force being applied against food at that location (as in bone-cracking hyaenids Ferretti 2007)? Can the teeth be allocated to particular functional forms using mammalian schemata (e.g., those in Evans and Sanson 2003)? Would the occlusion patterns for the teeth on astigmatid cheale be useful as in crustacean chelipeds (Brown et al. 1979)? For sure teeth variation is variably correlated with ecomorphological guilds and phylogeny in tadpoles; Candioti and Altig (2010). Do teeth vary over the astigmatid phylogeny as in voles (Ledevin et al. 2010)? Perhaps a carefully chosen Fourier analysis of the moveable digit outline (including its teeth) as used in comparative rodent (Navarro et al. 2004; Firmat et al. 2010) or moth (Monti et al. 2001) studies would be illuminating? Could explicit tooth complexity measures as used in bat ecomorphology (Santana et al. 2011) help?

### Enzymology and mite habits

Figure 29 shows those astigmatid species where hide protease activity is available (from Bowman 1981, 1984) plotted on the notional ‘speed of closing’ versus notional ‘food toughness’ display. Hide is a structural protein, however, casein protease levels are positively correlated with hide protease activity in astigmatids (Bowman 1984). The enzymic activity for those 10 species above the dashed threshold (at $VR*PHI^{2}=1039.0$ which is the proposed boundary for morphologically described feeding types described above) are significantly different than those 11 species below (Welch’s *t*-test $p=0.0013$). Whilst the average $\mu \text {g}$ trypsin/mg BSA for the lower group is low at 0.3476, the upper group’s mean of $1.3845\,\mu \text {g}$ trypsin/mg BSA even exceeds that of the range of levels in true predatory mesostigmatids like *Pergamasus longicornis* (0.82–1.27; Bowman 1987)! This indicates these astigmatids in the upper group (which includes pest species such as *Acarus siro*, *Tyrophagus longor*, *Glycyphagus domesticus*, and *Lepidoglyphus destructor*) as protein-consuming specialists and shows consilience with their putative origin as necrophages. Examining more species would confirm this.

Other enzymic validations have already been discussed above but confirmations could also be sought using fatty acid (NLFA) analyses, amino acid analyses, and molecular gut content analyses of astigmatids (electrophoresis and iso-electric focusing of gut extracted material has already been carried out in other groups). These non-morphological investigations could be targeted to particular individual species as necessary. Different habitat cross-classifications (e.g., Akimov and Oksentyuk 2018) could be used in a sensitivity analysis. Fractionating astigmatid species further into: phyllophages, mycophages, bacteriophages, phycophages, etc., as originally proposed by Luxton (1972) for oribatids could be attempted by say more comparative enzymic studies in astigmatids.

More work in delineating a wider set of non-pyroglyphid astigmatids into ‘shredders’ who ingest material like leaves and associated microbes by biting off chunks, versus ‘scrapers’ ($=$ grazers) who crop fungal hyphae and/or other microbes and detritus from the substrate surface, versus ‘collectors’ who filter microbes and fine particulate matter from fluids as used by Fashing (1999) for the acarine inhabitants in food webs of water-filled tree holes would help. Using other such classifications (see Pande and Berthet 1973) could be done morphologically. Particularly, is there a special pollenophagous astigmatid design that would help explain florally or bees-nest associated astigmatids like *Kuzinia laevis*? Confirmation that oophagous/necrophagous sources are protein-rich would be useful, as would finding comparatively elevated protease digestive enzymes (or metabolism favouring high nitrogen sources) of a scale comparable to other mesostigmatid carnivores for all pest species versus non-pest astigmatids. Is the pearly-white idiosomal colour of most astigmatids and their lack of malpighian tubules indicative of a particular nitrogenous physiology?

### Other taxa

Other mite individual species could be examined. For instance, using this review’s approach, what could *Gohieria fusca* (Labidophorinae) also found in bagged flour (Evans et al. 1961) tell us? What about the ancestral acaroid *Glycacarus combinatus* (see Griffiths 1977)? Are the *Caloglyphus* spp. found in bat guano (Hughes 1959) distinct? Are they consilient with the results for the bat habitat astigmatid species reviewed herein (*Acarus gracilis*, *Glycyphagus domesticus*? What about *Thyreophagus corticalis* which occurs on fungus-infested tree-bark and decaying reeds (Hughes 1959)? What do other *Tyroborus* spp. look like (Fan and Zhang 2006)? Does *Carpoglyphus munroi* found in bat roosts (Hughes 1959) have feeble tweezer-like chelicerae like *Carpoglyphus lactis*? *Carpoglyphus nidicolus* is found in swallow nests apparently (Hubard and Fashing 1996), but what could it be consuming in such a potentially very dry environment? Would these astigmatids all be classed as fragment feeders using Kaneko (1988)’s logic of small size and low velocity ratio? Do other *Rhizoglyphus* spp. (Fan and Zhang 2003) match *Rhizoglyphus echinopus* R1 or R2 trophically? Are chaetodactylids found in the nests of wood-boring xylocopid and solitary osmid and megachild bees (Krantz 1971) distinct in chelal design? *Chaetodactylus* spp. are better termed kleptoparasites rather than predators of bee eggs and larvae, (OConnor 1982b). They like insect kleptoparasites, kill the hosts’ egg/larva and develop a population by feeding on the provisions of the host (Oconnor *pers. comm.*). Do they look like durophages or oophages or neither?

Many other possible free-living genera could be chosen (Krantz 1978; Krantz and Walter 2008). Observations concerning the role of astigmatid mites in ant nests are rare in the literature (Uppstrom and Klompen 2011). What do the chelicerae and chelae of myrmecophilous *Cosmoglyphus* and *Forcellinia* spp. look like? What does this mechanical framework suggest for semiaquatic hyadesids, at least some of which appear to be algophagous (OConnor *pers. comm.*)? Their cheliceral chelae look minute and elongate in illustrations (e.g., Fashing and Wiseman 1980). What about *Hericia* (Algophagidae, OConnor 1982b) which do not feed upon algae? *Hericia sanukiensis* does appear to have feeble tweezer-like chelicerae (Fashing and Okabe 2006). Other algophagids have strange carpoglyphid-like chelicerae (Fashing 1998, 1999), with one (*Algophagopsis* n.sp.) clearly adapted for crunching food. Carpoglyphidae and Algophagidae are clearly sister-groups (OConnor *pers. comm.*) and have many morphological traits in common. Algophagids evolved the peculiar “axillary organ” which is osmoregulatory and probably is useful in the aquatic/semi-aquatic habitats preferred by species in this family (OConnor *pers. comm.*). Do they have designs the opposite of those astigmatids deemed to be hard food feeders reviewed herein?

What do the chelicerae of *Tyrophagus zachvatkini* which feeds on injured and moulting arthropods together with anhydrobiotic nematodes (Walter et al. 1986), fungi and algae (Walter 1987) indicate? Does it look like other protein-rich feeder/necrophages? Is the distinct pyroglyphid design repeated in *Euroglyphus maynei*? Are all house dust mites designed to preferentially deal with protein deposits in bedding (see Colloff 2010)? What design are cavernicolous *Schwiebia* spp. which are found floating upon water (Vitzthum 1932)? What would a design solely for coprophagy or guanophilia look like? Is that what the distinctive bat-habitat form (e.g., *Acarus gracilis*, *Glycyphagus domesticus*) is indicating?

Looking at: multiple other winterschmidtiids (possibly *Winterschmidtia nataliae = Calvolia jraxini* which is a predator of the eggs and early instar larvae of the scolytid beetle *Leperisinus jraxini*; OConnor 1982b) or *Czenspinskia transversostriata* which damages apple leaves; Krantz and Lindquist 1979, or some hemisarcoptids (particularly *Hemisarcoptes malus* with its strange derived carpoglyphid-like shaped chelicerae, found on fruit trees predaceous on the eggs and adults of coccid scale insects; Hughes 1959, OConnor 1982b), or many more glycyphagoid species (OConnor 1982b perhaps including *Blomia*; Van Bronswijk et al. 1973), more *Aeroglyphus* spp. (Aeroglyphidae), or more pyroglyphid genera (e.g., *Euroglyphus*, *Pyroglyphus*, *Malayoglyphus*, etc.; Krantz 1971; Wharton 1976), plus *Melisia* and *Contramelisia* (Canestriniidae) who are insect associates of unknown feeding habits (OConnor *pers. comm.*) all would be useful to validate conclusions. Canestrinioids are very poorly known; OConnor (1982b).

More suidasiids could be interesting, not just *Suidasia pontifica* (often found in stored food products, and is a major problem in insect collections; OConnor *pers. comm.*) but *Tortonia quadridens* (*Tortonia* spp. are obligate associates of Hymenoptera, apids, sphecids and vespids; OConnor *pers. comm.*). The derived *Naiadacarus* spp. are supposed saprophages on decomposing leaves and arthropods that fall into tree-holes (Krantz 1971). However, Fashing has clearly demonstrated that they are leaf shredders (OConnor *pers. comm.*). Their chelicerae await measurement.

Nowadays NGS technology is beginning to be available to dissect out genera like *Tyrophagus* down to definitive species level; Murillo et al. (2018). What would posing this sort of mechanically based morphological review in the context of such a known astigmatid phylogeny (as done in newts for instance; Ivanović and Arntzen 2014), show? Mapping traits connected with their trophic ecology onto the molecular phylogeny of Astigmata (or better all of the Sarcoptiformes) would be a useful project for the future. Although advances have been made in placing the origin of astigmatids from within the desmonomatan oribatid lineage (Dabert et al. 2010; Pepato and Klimov 2015), for such a study a finalised modern, molecular phylogeny within the Astigmata is needed. This is being actively worked upon by Barry OConnor, Pavel Klimov and others.

### Behaviour and ecology

Is there any experimental evidence that the reviewed species denoted ‘interstitial’ actually burrow? Some parasitic astigmatids certainly do, sarcoptids and knemidocoptids burrowing in skin (Evans 1992). For sure, Robaux et al. (1977) reports the astigmatid mite *Tyrophagus putrescentiae* to be a geophage constructing pores and aerating the substrate. Spiders of several families excavate tunnels in the ground; Bristowe (1954), and oribatids too, even some burrowing into living plant tissue; Krantz and Lindquist (1979). Do the minute holes in leaves made by *Tyrophagus similis* or the lesions made by *Tyrophagus nieswanderi* on greenhouse plants result from the same digging action? It would truly be interesting to observe the behavior of acarids with obvious adaptations for burrowing, e.g., *Stereoglyphus*, *Acarotalpa*, some *Schwiebea*, etc. These have mole-like short robust anterior legs with large spine-like setae. Is this burrowing a core competency for arachnids? Was it formative in their early evolution? Could it related to any ventral strengthening as in early turtle evolution (Bowman 2021)? Do the demolition-excavation adaptations shown by the mouthparts of surface omnivore pest species facilitate such burrowing?

To some extent astigmatids represent a design zone of smaller oribatids. So at a wider level, is the trophic change and thus the selection pressure on mouthpart design engendered simply by Cope’s rule (Stanley 1973) applying to astigmatids? Predation is a trophic change in mesostigmatids (and other animals) correlated with increased size, so did a size change ‘force’ a change in foodstuff in the fragmentary feeding Sarcoptiformes first to protein-rich sources then to structurally rich (but tough) carbohydrate-rich sources, after all there are not giant fungi to eat!

Allometrically based polymorphism in male chelicerae has been demonstrated in several distinct families of Astigmata: the feather mites, Avenzoariidae (Bdellorhynchus), Falculiferidae (*Falculifer* and others) and the nidicolous Hypoderatidae (*Hypodectoides*); OConnor *pers. comm.* These, however, have been demonstrated to be related to fighting among males, at least in the two feather mite families. These males likely do not feed at all. Do free-living astigmatids show anything like this to confound the interpretation of their chelal designs?

Do astigmatid mites show chelicerae that vary between habitats within a species? For sure, skull shape and size varies in rodents with geography and environmental contexts; Fornel et al. (2010). Climate affects rodent tooth shape too; Piras et al. (2010). Fish vary in body shape and snout facies according to if they forage in simple or complex habitats; Ruehl et al. (2011). Trophic polymorphisms within a species are known in sticklebacks (e.g., Kristjánsson et al. 2002). Are mites like this too? Is there any evidence of modules of integration in the structures of the whole gnathosoma (including palps) much as found in mechanically important systems in fish; see Jamniczky et al. (2014). Would a richer geometric morphometric study with more landmarks over more structures detect ‘modularity’ (Bookstein 2018)? Can the pioneering work of Akimov and Oksentyuk (2018) be extended? Given that many astigmatids have short life-cycles and are associated with spatially-separated chance-accumulating temporary habits (Hughes 1959) embedded in a ‘sea of undesirability’ that they traverse by phoresy, could tracking cheliceral size and shape in populations over time (in the context of island biogeography theory) be useful, much as done for Galápagos finches e.g., Grant et al. (2000)? Can astigmatid community structure inferences be made like that done in lizards; Ricklefs et al. (1981)?

Despite the lack of importance of feathers and mammal association in Table 5, what about the design of chelicerae of parasitic/symbiotic astigmatids like: those on feathers e.g., *Proctophyllodes quadrisetosus*, *Bdellorhynchus polymorphus*, or those in the ears of cats and dogs e.g., *Otodectes cynotis*, or fur mites like *Myocoptes musculinus*, or mammal-associated listrophorids like *Listrophorus dozieri* or atopomelids like *Chirodiscoides caviae*? Are these designed in a certain way?

Could the highly derived chelicerae of filter-feeding histiostomatids such as species from the family Histiostomatidae found in sewage filter beds, plant pitchers, sap fluxes from damaged trees and other watery accumulations in the axils and inflorescences of tropical plants; (Hughes 1953, 1959; Fashing and OConnor 1984; Fashing 2004, 2010), be looked at mechanically using comparable methodology to that used by Zhang and Malmqvist (1996) in aquatic blackfly larvae? Thus segmenting amongst the astigmatid ‘planktonic’ feeding types. Do these chelicerae share any common functional features with the inner edges of the beaks of filter-feeding ducks? What look like fimbriate edges to chelal moveable digits have been illustrated for *Heterozercon audax* by Hirschmann (1959).

## Conclusion and field use

Is there an advantage in free-living Astigmatid chelicerae? Yes. Cheliceral structure and feeding ecology *do* appear correlated (just as Perdomo et al. 2012 found for oribatids). What an astigmatid mite ingests and what it chews on is not necessarily the same as what it really digests and assimilates, however, a clear ecomorphological link is found. Adaptive syndromes exist.

The overall conclusion for each acarine species is given in Tables 6 and 7. Then Fig. 20 overlays the stylised pictorial form of key mites upon the species loading plot of Fig. 5c showing how macro- to microsaprophagy divides the ordination vertically and Fig. 25a, b indicates the difference between surface and interstitial forms. It is stressed that macrosaprophagy versus microsaprophagy is not the same as oribatid macrophytophagy (and panphytophgy) versus microphytophagy (and fragmentary feeding). Rather the same trend in morphological distinctions of oribatid macrophytophagy to microphytophagy informs the separation of any intrinsically (oribatid classification-derived) ‘macro-micro-phytophagous/fragmentary feeding’ designed astigmatids into two differently designed distinct groups (cf. the two convex hull envelopes in Fig. 25c). The common stored product pest species (*Acarus siro*, *Glycyphagus domesticus*, *Lepidoglyphus destructor*, *Rhizoglyphus echinopus*, *Tyrolichus casei*, *Tyrophagus longior*, and *Tyrophagus nieswanderi*) are at the upper range of the lower envelope, or at the lower range of the upper envelope in Fig. 25c. Whilst microsaprophagous astigmatids almost certainly are strict consumers of fungi/microflora, some acaroid-like macrosaprophagous astigmatids (e.g., *Acarus siro* (A10b), *Glycyphagus domesticus* (G5), *Lepidoglyphus destructor* (G6), *Rhizoglyphus echinopus* (R1 & R2), etc.) can tackle much harder food material including higher plant material/leafy tissues which according to Krantz and Lindquist (1979) would make them ‘macrophytophages’. Yet these astigmatids are not exactly equivalent to oribatid macrophytophages in design (see Fig. 26a). Even macrophytophagous oribatids actually only feed on dead or dying tissue and thus technically are saprophages (Krantz and Lindquist 1979) being essentially secondary decomposers (i.e., in top right of Fig. 26c). From Fig. 26a, one can also see that the term ‘microphytophagous oribatid’ spans species which could be described as both hypocarnivorus/fragmentary feeding astigmatid-like and true secondary decomposers. Great care in labelling and use of old terms is needed.

Indeed, Perdomo et al. (2012) denotes species in the left hand convex hull of Fig. 26a as ‘carnivores’, and although this region covers the smaller mesostigmatids who may crunch nematodes, it also includes the uropodoids (Bowman 2021), many of which are not strictly predatory. This area should be labelled ‘hypocarnivores’. Microsaprophagous astigmatids (and oribatids) falling into this box may thus be facultative nematode feeders as well as grazers or gleaners. The region above the hypocarnivores might be considered a further type of ‘carnivore equivalent’ as this is where pests and those ‘shredding’ (*sensu* Fashing 1998) astigmatids favouring high protein deposits ($\equiv$ oophagy/necrophagy) preferentially plot (note $p<0.001$ for food toughness; Table 10). Could it be that this ‘vegan carnivore’ or protein-seeking behaviour is a signal for species tuned for rapid population growth (i.e., an *r*-strategy) needing high N input? The mechanical model approach and ordinations in this review are turning out to be very useful. Whilst oribatid data informs the interpretation of astigmatid differentiation (particularly those above velocity ratio $VR=0.5$, where they may be more tuned for a long-life (i.e., a *K*-strategy?), nothing in this review should be interpreted as inferring that the two sub-orders are phylogenetically related. Indeed some authors, e.g. Domes et al. (2007), using molecular markers do not even support the accepted origin of Astigmata within the Oribatida.

A nomogram for use by ecologists in the field is given in Fig. 21. Astigmatid mites vary in general size. Within that they vary almost independently in a type of ‘gnathosomisation’ (i.e., increased cheliceral/chelal height), and with that they vary approximately independently in mechanical advantage (chelal design). These patterns do not strongly match the popular cross-classifications of habitat. Including the estimated chelal adductive force between cheliceral chelal digit tips is much more useful (Figs. 24c, 27). Any apparent absence of fine tuning in mites to their competitive milieu is probably a function of the variable micro-environments in which they coexist (Rotenberry 1980) and the subtleties of how they might interact trophically (Pimm and Pimm 1982).

Notwithstanding minor deviations, astigmatids *do* show ecomorphological correlates (Fig. 27) potentially useful for indicating the likely food habits (and pest status) of a sample (*n*) of new such mites collected in the field. This prediction can be straightforwardly done by ecologists through:

- measuring each individual ($i=1\ldots n$) and calculating the mean ($\mu$) and sd ($\sigma$) for their *IL*, *CLI*, *CHI*, *L*2*M*, *L*1*U*, in $\mu$m and calculating the derived *F*2*AV* measure for their field sample,
- inserting these values into the quadratic discriminant (Eq. (1) from the “Materials and methods” above) for each specimen ($i=1\ldots n$), just as was done for the known species (in Table 2 of this review),
- estimating the location of each of the $i=1\ldots n$ individuals from the new sample calculated with the component equations on the nomogram in Fig. 21,
- choosing the nearest small set of known astigmatid species on this nomogram to the *n* unknown field specimens (and their average over the plot), and
- then, using their macro- or micro-saprophagous stylised forms in Fig. 20, together with the groups summarised in Figs. 21, 22, 23, 24 and 25, drawing interim conclusions from Figs. 15, 16, 17, 18 and 19 regarding the habits of the field species sample pending confirmatory tests inferred by their heuristic positioning (Figs. 11, 12, 13, and 14).

Figs. 27 and 28 can then inform the detail of conclusions.

If only a single specimen is available from a field sample ($x_{k},\ k=1\ldots 6)$ then an approximate prediction on a continuous scale can be made by calculating the square root of the sums of squares difference from that specimen’s *IL*, *CLI*, *CHI*, *L*2*M*, *L*1*U*, and the derived *F*2*AV* measures to each of the rows separately in Table 2, i.e.,

$$\begin{aligned} d_j=\sqrt{\sum _{k=1}^{6}(x_k-\mu _{j,k})^2}\quad (j=1\ldots 47) \end{aligned}$$

and that taxon (*j*) with the lowest $d_j$ chosen as its ‘match’. Accordingly then the trophic categorisation from Tables 6 and 7 can be allocated to the unknown specimen and conclusions drawn as before. An even more ’rough and ready’ categorisation (based on ’lookup’ only suitable for high-throughput screening) could be done by simply scoring the measurements of the single field sampled individual as “above (+ 1)” or “below ($-1$)”, the average of the reference typical mite values in Table 2. Then matching that string of +1 and − 1s to the examples as in ordered Table 11. If there was no exact match then calculating a Manhattan-style distance (of the number of matching elements) to the nearest taxon could be done. This heuristic process has the advantage that it can be carried out even if not all morphological (*M*) or design (${\hat{D}}$) measures were available for that individual field specimen.

This process is commended for field acarologists to use.

## Footnotes

1 For the distinction between functional groups and guilds; see detail and references in Walter and Proctor (2013).
